# Supplementary material for: REST: a preoperative tailored sleep intervention for patients undergoing total knee replacement – feasibility study for a randomised controlled trial
Source: BMJ Open. 2024 Mar 20;14(3):e078785. doi: 10.1136/bmjopen-2023-078785 (PMC10961562; doi:10.1136/bmjopen-2023-078785)
Supplement: Supplementary data [file bmjopen-2023-078785supp001.pdf]

Appendix 1: CONSORT 2010 checklist of information to include when reporting a pilot or feasibility randomised trial in a journal

Item	Description	Reported on line number
Title	Identification of study as randomised pilot or feasibility trial	1
Authors *	Contact details for the corresponding author	9
Trial design	Description of pilot trial design (eg, parallel, cluster)	90
Methods		
Participants	Eligibility criteria for participants and the settings where the pilot trial was conducted	103
Interventions	Interventions intended for each group	122
Objective	Specific objectives of the pilot trial	160
Outcome	Prespecified assessment or measurement to address the pilot trial objectives	169, Table 1
Randomisation	How participants were allocated to interventions	118
Blinding (masking)	Whether or not participants, care givers, and those assessing the outcomes were blinded to group assignment	119
Results		
Numbers randomised	Number of participants screened and randomised to each group for the pilot trial objectives	228
Recruitment	Trial status	N/A
Numbers analysed	Number of participants analysed in each group for the pilot objectives	324, 368
Outcome	Results for the pilot objectives, including any expressions of uncertainty	Objective 1: 228<br>2: 250<br>3: 256<br>4: 271<br>5: 300<br>6: 309<br>7: 315<br>8: 342<br>9: 366
Harms	Important adverse events or side effects	292
Conclusions	General interpretation of the results of pilot trial and their implications for the future definitive trial	448
Trial registration	Registration number for pilot trial and name of trial register	92
Funding	Source of funding for pilot trial	456

Citation: Eldridge SM, Chan CL, Campbell MJ, Bond CM, Hopewell S, Thabane L, et al. CONSORT 2010 statement: extension to randomised pilot and feasibility trials. BMJ. 2016;355.

Appendix 1: CONSORT 2010 checklist of information to include when reporting a pilot or feasibility trial

Section/Topic	Item No	Checklist item
**Title and abstract**		
	1a	Identification as a pilot or feasibility randomised trial in the title
	1b	Structured summary of pilot trial design, methods, results, and conclusions (for specifi CONSORT abstract extension for pilot trials)
**Introduction**		
Background and objectives	2a	Scientific background and explanation of rationale for future definitive trial, and reasor trial
	2b	Specific objectives or research questions for pilot trial
**Methods**		
Trial design	3a	Description of pilot trial design (such as parallel, factorial) including allocation ratio
	3b	Important changes to methods after pilot trial commencement (such as eligibility criter
Participants	4a	Eligibility criteria for participants
	4b	Settings and locations where the data were collected
	4c	How participants were identified and consented
Interventions	5	The interventions for each group with sufficient details to allow replication, including h actually administered
Outcomes	6a	Completely defined prespecified assessments or measurements to address each pilot 2b, including how and when they were assessed
	6b	Any changes to pilot trial assessments or measurements after the pilot trial commenc
	6c	If applicable, prespecified criteria used to judge whether, or how, to proceed with futur
Sample size	7a	Rationale for numbers in the pilot trial
	7b	When applicable, explanation of any interim analyses and stopping guidelines
Randomisation:		
Sequence generation	8a	Method used to generate the random allocation sequence
	8b	Type of randomisation(s); details of any restriction (such as blocking and block size)
Allocation concealment mechanism	9	Mechanism used to implement the random allocation sequence (such as sequentially describing any steps taken to conceal the sequence until interventions were assigned
Implementation	10	Who generated the random allocation sequence, who enrolled participants, and who a interventions
Blinding	11a	If done, who was blinded after assignment to interventions (for example, participants, assessing outcomes) and how
	11b	If relevant, description of the similarity of interventions
Statistical methods	12	Methods used to address each pilot trial objective whether qualitative or quantitative

2

**Results**		
Participant flow (a diagram is strongly recommended)	13a	For each group, the numbers of participants who were approached and/or assessed f assigned, received intended treatment, and were assessed for each objective
	13b	For each group, losses and exclusions after randomisation, together with reasons
Recruitment	14a	Dates defining the periods of recruitment and follow-up
	14b	Why the pilot trial ended or was stopped
Baseline data	15	A table showing baseline demographic and clinical characteristics for each group
Numbers analysed	16	For each objective, number of participants (denominator) included in each analysis. If should be by randomised group
Outcomes and estimation	17	For each objective, results including expressions of uncertainty (such as 95% confide estimates. If relevant, these results should be by randomised group
Ancillary analyses	18	Results of any other analyses performed that could be used to inform the future defini
Harms	19	All important harms or unintended effects in each group (for specific guidance see CONS
	19a	If relevant, other important unintended consequences
**Discussion**		
Limitations	20	Pilot trial limitations, addressing sources of potential bias and remaining uncertainty a
Generalisability	21	Generalisability (applicability) of pilot trial methods and findings to future definitive trial
Interpretation	22	Interpretation consistent with pilot trial objectives and findings, balancing potential ber considering other relevant evidence
	22a	Implications for progression from pilot to future definitive trial, including any proposed
**Other information**		
Registration	23	Registration number for pilot trial and name of trial registry
Protocol	24	Where the pilot trial protocol can be accessed, if available
Funding	25	Sources of funding and other support (such as supply of drugs), role of funders
	26	Ethical approval or approval by research review committee, confirmed with reference

Citation: Eldridge SM, Chan CL, Campbell MJ, Bond CM, Hopewell S, Thabane L, et al. CONSORT 2010 statement: extension to randomised pilot and feasibility trials. BMJ. 2016;355.

Appendix 2: The TIDieR (Template for Intervention Description and Replication) Checklist

TIDieR
Template for Intervention Description and Replication

The TIDieR (Template for Intervention Description and Replication) Checklist*:

Information to include when describing an intervention and the location of the information

REST: A pre-operative tailored sleep intervention for patients undergoing total knee replacement: feasibility study for a randomised controlled trial

Item number	Item	Where located ** Primary paper (page or appendix number)	Other † (details)
	**BRIEF NAME**		
**1.**	Provide the name or a phrase that describes the intervention.	__page 4__	______________
	**WHY**		
**2.**	Describe any rationale, theory, or goal of the elements essential to the intervention.	__page 3_____	______________
	**WHAT**		
**3.**	Materials: Describe any physical or informational materials used in the intervention, including those provided to participants or used in intervention delivery or in training of intervention providers. Provide information on where the materials can be accessed (e.g. online appendix, URL).	__page 4-5__	______________
**4.**	Procedures: Describe each of the procedures, activities, and/or processes used in the intervention, including any enabling or support activities.	__page 4-5___	______________
	**WHO PROVIDED**		
**5.**	For each category of intervention provider (e.g. psychologist, nursing assistant), describe their expertise, background and any specific training given.	__ page 4-5__	______________
	**HOW**		
**6.**	Describe the modes of delivery (e.g. face-to-face or by some other mechanism, such as internet or telephone) of the intervention and whether it was provided individually or in a group.	__ page 4-5__	______________
	**WHERE**		

**7.**	Describe the type(s) of location(s) where the intervention occurred, including any necessary infrastructure or relevant features.	___ page 4-5__	______________
	**WHEN and HOW MUCH**		
**8.**	Describe the number of times the intervention was delivered and over what period of time including the number of sessions, their schedule, and their duration, intensity or dose.	__ page 4-5__	______________
	**TAILORING**		
**9.**	If the intervention was planned to be personalised, titrated or adapted, then describe what, why, when, and how.	___ page 4-5__	______________
	**MODIFICATIONS**		
**10.‡**	If the intervention was modified during the course of the study, describe the changes (what, why, when, and how).	___ N/A____	______________
	**HOW WELL**		
**11.**	Planned: If intervention adherence or fidelity was assessed, describe how and by whom, and if any strategies were used to maintain or improve fidelity, describe them.	___page 8-9__	______________
**12.‡**	Actual: If intervention adherence or fidelity was assessed, describe the extent to which the intervention was delivered as planned.	___page 8-9__	______________

** **Authors** - use N/A if an item is not applicable for the intervention being described. **Reviewers** – use '?' if information about the element is not reported/not sufficiently reported.

† If the information is not provided in the primary paper, give details of where this information is available. This may include locations such as a published protocol or other published papers (provide citation details) or a website (provide the URL).
‡ If completing the TIDieR checklist for a protocol, these items are not relevant to the protocol and cannot be described until the study is complete.

* We strongly recommend using this checklist in conjunction with the TIDieR guide (see *BMJ* 2014;348:g1687) which contains an explanation and elaboration for each item.

* The focus of TIDieR is on reporting details of the intervention elements (and where relevant, comparison elements) of a study. Other elements and methodological features of studies are covered by other reporting statements and checklists and have not been duplicated as part of the TIDieR checklist. When a **randomised trial** is being reported, the TIDieR checklist should be used in conjunction with the CONSORT statement (see www.consort-statement.org) as an extension of **Item 5 of the CONSORT 2010 Statement.** When a **clinical trial protocol** is being reported, the TIDieR checklist should be used in conjunction with the SPIRIT statement as an extension of **Item 11 of the SPIRIT 2013 Statement** (see www.spirit-statement.org). For alternate study designs, TIDieR can be used in conjunction with the appropriate checklist for that study design (see www.equator-network.org).

Appendix 3A: Patient interview topic guide

REST patient topic guide

Topic guide to be used flexibly. Interviewer will use probes and follow-up questions where appropriate.

Introduction: Discussion of how the interview will be recorded, issues of confidentiality, anonymisation. **Aim of the study:** To understand patient's perspectives of participating in the study, recruitment, and intervention experiences. **Verbal consent procedure**: Consent for interview, **Any questions?**

Introduction / icebreaker

- Thank you for taking part in the interview. To start us off, could you tell me a little about your **knee pain in the lead up towards your surgery** and the **impact that it has had on your sleep?** PROMPT: What problems have you had? Effect on sleep quality/quantity? Impact on daily activities/wellbeing?
- Have you **taken part in any research studies before?** PROMPT: What? When? What was it like taking part?

Design and conduct of the trial (<u>all participants</u>)

- I'd like to talk to you about **when you first heard** about the REST study. Can you tell me how you found out about it?
- What were your **first thoughts** about taking part?
- Can you tell me ***why*** **you decided to take part**? PROMPT: Did you discuss your decision with anyone else, for example friends/family member?
- Did you have **any initial concerns** about being involved in the REST study? PROMPT: Could you tell me about them? What helped you to resolve/overcome these concerns?
- Do you still have **any concerns now**, about either the sleep intervention or your participation in the study itself? PROMPT: Expectations versus reality.
- After you expressed your interest in taking part in the REST study, one of the team would have **given** you **a call to discuss the study** with you in more detail. What was having that conversation like for you? PROMPT: Can you recall what information they told you?
- Overall, **what do you think about the information that you have received about the study**: (i) during the initial telephone call, (ii) in the information booklet. PROMPT: Was there enough information / right kind of information? Was it understandable? Was anything missing?
- **How do you feel about being put randomly into a group** to receive either the sleep treatment or usual care? PROMPT: How acceptable do you feel this is?
- **What do you think about the questionnaires** that you have completed, about your knee pain and sleep? PROMPT: Amount of questions, have the questions been relevant, any sections difficult to complete?

Experiences of the sleep intervention (for <u>intervention group only</u>)

- Now I'd like to talk to you about your experiences of the sleep appointment with [NAME] and the advice you were given during it. Overall, **how did you find the sleep appointment**?
- **What did you think about having a telephone or remote / video appointment**? (If relevant) PROMPT: What was it like for you? Pros and cons, did you have any concerns/problems, what device did you use, familiarity with video conferencing, any support needed (e.g., from family member/friend) with setting it up?
- **How did you find the length of the appointment?** PROMPT: Too long/too short. Too rushed or okay. Enough time to ask questions? Did appointment run to time?

- What **advice did the practitioner give** to you about your sleep during the appointment? PROMPT: What was most/least helpful? What do you think about the way in which they gave this information to you? Was it understandable?
- How did you find **talking about/opening up about your sleep** (problems) with the practitioner?
- Which **sleep treatment option did you choose**? PROMPT: CBT-I, relaxation, mindfulness
- **How involved** did you **feel in choosing** this option? PROMPT: How did the practitioner support you in making this decision? Were you given enough information to help you understand the different options?
- Before taking part in the REST study, did you have any **experience or knowledge of CBT, relaxation or mindfulness** techniques? PROMPT: How do you feel this has influenced your experience in the study?
- **What do you think about the sleep treatment so far**? PROMPT: How are you finding doing it, barriers to engagement, facilitating factors (e.g., partner support), what have you enjoyed/not enjoyed, are you finding it helpful, digital and non-digital options (including ease of use and if any support needed to access digital options), (if appropriate) changing experience over time.
- Did you **make any other changes to your sleep** after the appointment? PROMPT: Sleep aids, apps, sleep hygiene.
- How did you find the **4-week follow up phone call** with the practitioner? PROMPT: What was useful/not useful about it. As a result of the phone call, did you make any changes? If appropriate, use participant's intervention uptake questionnaire to guide questioning.
- Overall, **do you feel the appointment and treatment option has had an impact on your sleep**? PROMPT: What impact has it had? What has had the most impact (e.g. chatting to a professional, increasing knowledge/understanding, engaging with the techniques)?
- **Do you think the sleep appointment and treatment option** you chose has had an **impact on your knee pain**? PROMPT: What impact has it had?
- Would you have liked **any further information or additional support** about your sleep or the treatment you chose? PROMPT: When/how would you have liked to receive this?

For usual care group only

- **Since being recruited** into the REST study, has your **sleep changed**? PROMPT: how has it changed, why, when?
- Have you **tried anything to improve your sleep**? PROMPT: what have you tried, how helpful has it been, when did you try this (i.e. prior to or during the REST study)?
- **Since being recruited** into the REST study, has your **knee pain changed**? PROMPT: how has it changed?

Conclusion (all participants)

- Thinking now about your whole experience of taking part in the REST study, **how could we have improved the way in which the study was organised and run**?
- Do you feel that there are **any ways in which your sleep appointment,** or the **follow up phone call, could have been improved**?
- Is there **anything else** you would like to add/talk about that we haven't covered already?
- **Thank participant for their time**. If appropriate, **signpost** participant **to the 'Useful Contacts' sheet.**
- Ask participant if they would like to receive a **brief report** containing the **key findings** from the interview study.

Appendix 3B: ESP intervention training interview topic guide

REST ESP topic guide – intervention training

Topic guide to be used flexibly. Interviewer will use probes and follow-up questions where appropriate.

Introduction: Discussion of how the interview will be recorded, issues of confidentiality, anonymisation.
Aim of the study: To understand experiences of the training session.
Consent procedure: Check written consent, complete consent form if not already done.

Participant information

- **Year of qualification/Years in practice**
- **Role** at the hospital
- **Experience** of participating in **other trials or research** (PROMPT: any experience with sleep interventions, personal experience)

Background information and general sleep education

- I would like to start by asking you about your **experience/overall impression** of the remote training session. **What did you think about the session in general?** (PROMPT: What did you like? What did you not like? What did you enjoy/not enjoy? One long session vs two shorter over two days?)
- Did you **prepare** in any way for the session? (PROMPT: background reading)
- Thinking now about the **information** that you were given **on sleep** and **why sleep is important**:
  - **How understandable** was it?
  - **How useful** did you find it?
- Is there any **additional background information** that you would have **liked to have received** during the training?
- **How confident are you feeling now** about **talking about the background information** with the trial participants? (PROMPT: if not, what additional support / information would you like? How would you like to receive this?)

Appointment delivery

- How do you **plan to structure** your **sessions**? (PROMPT: Recommended timings)

Assessment

- What is your **understanding of the assessment process**?
- Do you **feel clear** / **confident** about **how to carry out the assessment**? (PROMPT: Using the assessment tool)
- **How do you feel** about **eliciting this information** from the participant?
- What did you think about the **assessment role-play exercise**? (PROMPT: What did you learn from it? How helpful/useful did you find it? Any suggestions about how it could have been done differently? Did you find time to practice the role play exercise after the training?)
- What do you think will be the **main challenges with the assessment process**?

Intervention delivery

- **What did you think** about the **sleep hygiene and education information**? (PROMPT: What did you like/What didn't you like?)
- Is it **clear how to tailor advice** to each participant? (PROMPT: Using the assessment table)
- **What did you think** about the **information** you received **on the specific sleep interventions** you will be recommending?
- **How will you choose** which sleep **intervention** is **most appropriate** for a participant?
- What is your **understanding of the behavioural contract**? (PROMPT: Confident using it, how to choose which areas to highlight, purpose of contract, setting SMART goals)
- What do you think will be **the main challenges with** the **intervention delivery**?

Conclusion

- **How are you feeling about** your **first appointment**? (PROMPT: Do you feel prepared? Is there anything that you will do between now and then to feel more prepared? Is there anything that you are still not sure about/want to know?)
- Is there **any information that you would have liked** in the training that **wasn't provided**?
- Was there **any information** in the training, which you felt was **not useful** / **needed**?
- Do you have any **suggestions on how to improve the training**?
- Overall, what did you find **most useful** about the training?
- What do you **think about the training manual**? Any **suggestions for improvement**?
- What were **the advantages and disadvantages** of doing the **training over Zoom**?
- Is there **anything else** that you would like to add, or anything you wish to talk about that we haven't covered already?
- **Thank you** for talking to me/your time/**when will be in contact again**.

END

*** Ask ESP about possibility of observing some of their intervention appointments with patients who have not consented to take part in an interview – in order to learn more about the process/patient experience***

Appendix 3C: ESP intervention delivery interview topic guide

REST ESP topic guide – intervention delivery

Introduction: *Discussion of how the interview will be recorded, issues of confidentiality, anonymisation.*
Aim of the study: *To understand experiences of delivering the intervention and any additional training needs.*
Verbal consent procedure: *Reaffirm consent for interview (written consent already given).*

Preparation for delivery

When we first spoke, you told me that before your first intervention appointment, you planned to do [XX]. Did you do this? Did you do anything else to prepare for the appointments? How did undertaking these activities help you to feel more prepared/confident?

Intervention appointment

- **How many sessions** delivered/mode of delivery and **overall experience of delivering** the **sessions: main challenges - for them & patients** (e.g., pragmatic challenges - timing/contacting patients/accessing Zoom/meeting remotely) - & **how overcome**
- **How have you found** undertaking the **assessment process and challenges**? (e.g., Know what questions to ask, eliciting the right information, following the assessment table, drawing out info from patients about sleep issues, patient engagement)
- **How** do you **go about choosing** which **sleep intervention** is best for a participant and any **challenges**? (e.g., selecting most appropriate intervention; shared decision making; patients choosing alternative option)
- **Experience of agreeing the behavioural contract**? (How do you decide on what to include, do participants engage, do you think it is helpful)
- How has the way in which **you deliver the intervention appointment changed over time**? (Refinements made. Increased confidence over time?)
- Impact of mode of delivery on patient engagement/disclosure
- What kind of **questions** have **participants asked** you during their assessment appointments? (Have you felt that you have had the appropriate knowledge/skills to address their questions?)
- **What outcomes would define a successful appointment** – for you and for the participant?

Sleep intervention set up and referral procedure

- **Experience of setting up the interventions** (e.g. clear what to do/what information needed) **& challenges** (e.g. free Headspace trial already used? how was this managed/what did they recommend)
- **Sleepstation referral process** (Any challenges, how long has it taken)
- **Information and support participants want** about **getting started with the sleep interventions**? (Able to give patients the support/info they needed? Paper versions of the documents requested?)

Follow-up phone call

- **How have you found** doing the **4-week follow-up phone calls**? (Challenges getting hold of patients, any

Conclusion

- **Additional training or information needs**? (How/When/Why)
- Thoughts on how **training itself, manual** and **documents could be improved**? (i.e. changes needed)
- **Recommendations for refinements** needed to improve way in which the intervention is delivered? (What do you think has worked well? What hasn't worked so well?)
- How has being part of REST/your REST role **benefited you either personally or professionally**?

***Explore confidence around setting the SMART goals

***Do they feel they understand the theory behind the different interventions and are they able to communicate it to patients?

***Do they feel that they have a good understanding of what SleepStation involves for a patient and are they able to/do they communicate this to patients?

***Have they kept a reflective diary?

Appendix 4: Participant outcomes and health economic data

Comparison of SCI between patients eligible at screening and randomized trial participants

Characteristic	In trial, N = 57[1]	Not in trial, N = 201[1]	p-value[2]
**SCI_Score**	13 (8, 17)	17 (10, 26)	0.004
Unknown	0	49	
[1]Median (IQR)			
[2]Wilcoxon rank sum test			

Pain outcomes at 3 months postoperative (Oxford Knee Score)

Characteristic	Intervention, N = 6[1]	95% CI[2]	Control, N = 9[1]	95% CI[2]
**On-going pain (OKS<14)**				
No on-going pain	5 (83%)	36%, 99%	8 (100%)	60%, 100%
On-going pain	1 (17%)	0.88%, 64%	0 (0%)	0.00%, 40%
Unknown	0		1	
[1]n (%)				
[2]CI = Confidence Interval				

Neuropathic pain outcomes at 3 months postoperative

	**Baseline**		**Pre-operative**		**Post-operative**	
**Characteristic**	Intervention, N = 28[1]	Control, N = 29[1]	Intervention, N = 15[1]	Control, N = 25[1]	Intervention, N = 6[1]	Control, N = 9[1]
**PainDETECT score**	18 (11, 21)	13 (9, 19)	11 (10, 17)	14 (10, 24)	7 (6, 12)	10 (6, 16)
	4	4	6	5	2	3
**PainDETECT category**						
Ambiguous	6 (25%)	5 (20%)	3 (33%)	5 (25%)	0 (0%)	1 (17%)
Neuropathic likely	9 (38%)	8 (32%)	1 (11%)	7 (35%)	1 (25%)	1 (17%)
Nociceptive	9 (38%)	12 (48%)	5 (56%)	8 (40%)	3 (75%)	4 (67%)
Unknown	4	4	6	5	2	3
[1]Median (IQR); n (%)						

EQ-5D-5L and ICECAP scores

Measure	Baseline			Pre-operation			Post-operation		
	Intervention (SD)	Usual care (SD)	Response	Intervention (SD)	Usual care (SD)	Response	Intervention (SD)	Usual care (SD)	Response
EQ-5D-5L	**0.34** (0.30)	**0.47** (0.21)	57/57	**0.35** (0.22)	**0.40** (0.23)	40/57	**0.63** (0.24)	**0.69** (0.13)	14/27*
ICECAP	**0.71** (0.26)	**0.78** (0.14)	56/57	**0.71** (0.23)	**0.80** (0.14)	40/57	**0.83** (0.25)	**0.91** (0.021)	15/27*

*Only collected for those who had TKR within trial.

Intervention costs

**Treatment Cost per person**	**Mean**	**Max**	**Min**
Staff	£103.73	£214.50	£33.00
NHS Treatment cost[§]	30.72	£61.45	£12.29
Societal treatment cost[¥]	£6.59	£19.78	£0
NHS Total (per person)	£134.45	£275.95	£45.29
Societal Total (per person)	£141.04	£295.73	£45.29

[§] Mean - 50% discount given to NHS, Max -no discount, Min - 80% discount offered to NHS.
[¥] Mean -1/3 of participants paid for Headspace and Calm app subscriptions, Max all pay for subscriptions, Min- no one pays for subscription.
*https://www.nice.org.uk/process/pmg36/chapter/economic-evaluation

Appendix 5: Demographics of interviewed participants

Pseudonym	Gender	Age at interview	Intervention or Usual Care	Mode of intervention delivery	Chosen sleep intervention
Florence	Woman	90	Intervention	Telephone	Relaxation
George	Man	64	Usual	N/A	N/A
Joyce	Woman	73	Usual	N/A	N/A
Gail	Woman	64	Usual	N/A	N/A
Charles	Man	79	Intervention	Video	CBT-I
Patricia	Woman	69	Intervention	Video	CBT-I
Gloria	Woman	73	Intervention	Video	Mindfulness
Ruth	Woman	72	Intervention	Video	CBT-I
Arthur	Man	72	Usual	N/A	N/A
Edward	Man	69	Intervention	Telephone	Mindfulness
Jerry	Man	64	Intervention	Video	Relaxation
Steven	Man	58	Usual	N/A	N/A
Rose	Woman	68	Intervention	Video	Mindfulness then CBT-I

Appendix 6: Qualitative themes and quotations

Theme	Subtheme	Quotations
**3. Explore engagement with the REST intervention and adherence to the agreed sleep plan**	Practitioner manner and communication style	*Well I think I was speaking to a professional and when I feel that, that gives me a little confidence in talking to her […] and I found that with [the practitioner] she was really very patient and in discussing her ideas […] she was very encouraging actually …[Florence]*<br>*It's been good. Just talk about things and get it into the open, don't bottle everything up [...] it suits me fine. [Jerry]*<br><br>*Well she was quite firm I felt in telling me about relaxing and how I would relax and she really wanted me to make up my mind then and there and tell her what I was planning to do and that made me feel more positive about it [...] she made me discuss it and having done that I was able to make my own mind up… she was suggesting breathing exercises as well […] there was no insistence at all from her, but on the other hand she was very firm in her suggestions [yes] which made me feel that I must do those things [Florence]*
**1. Evaluate intervention acceptability to patients and health professionals**	Patient acceptability: delivery mode	*I'm using Zoom with family etcetera, so I've used it for over a year now and I'm happy with it […] I'm confident using it [Patricia]*<br><br>*I'm not good on this Wi-Fi stuff, I'm really quite awful on it and my poor son was trying to train me [...] I don't know how to cope or how to use anything with Wi-Fi, I find it very irksome, I really do, very overwhelmingly worrying, I just worry about it. [Florence]*<br><br>*I think this sort of appointment you don't need to be face-to-face. [...] There's no point doing face-to-face. Yes, it would be a 50-mile round trip for me to come to you […] It's fine seeing each other like this to be honest. [...] Travel time, parking, petrol. It's saving a fair bit of money from my point of view. [Patricia]*<br><br>*I don't see a disadvantage. Sitting in my own home rather than going to a hospital where I could pick up more diseases or COVID again. [Gloria]*
	Patient acceptability: appointment length and structure	*There was somebody there who could give me suggestions on the way whilst waiting for my operation, do you know what I mean? It's almost as though she was giving me something to do and think about as I approached the operation … [Florence]*

		The phone call took place not at the right time. But that was fine by me because I wasn't doing anything else. [Patricia]<br><br>*I'm not pleased, I've taken time off work to do this, but I was happy to take time off work to do it but not to be able to do it… [Ruth]*
	Practitioner acceptability: mode of delivery	*I'd be the first to admit that my IT is not as good as it should. I don't do much in the way of IT. […] I'm not the slickest at getting it all up on the computer. […] I find the IT thing a bit of a challenge sometimes [ESP03].*<br><br>*They've all been there ready and waiting and a few times, I've been late finishing the morning clinic because I normally do them on a Monday afternoon. They've all been ready and waiting at their phone or their computer. [ESP03]*
	Practitioner acceptability: intervention handbook and paperwork	*The form was very easy to follow for all the questions [ESP01]*<br><br>*The actual booklet that you're given yes, that's easy. I think maybe I probably need to introduce it a little bit better and what the study is, but then the actual questions are easy to follow, and then it follows onto the SMART goals and things, yes. [ESP01]*<br><br>*I think overall fine, I think sometimes you do feel like you're duplicating quite a lot I think, and when I was actually working through paperwork too quickly and not really sitting down and really going in depth about what the question's asking. Sometimes it feels like, well see above, kind of thing. Maybe that's because I'm classically the kind of person that will write everything down in the first box [RE 4-week call paperwork] [ESP04]*
**6. Evaluate the acceptability of randomisation**		*I understand that that's the only way you can gauge whether what you're doing is of any benefit if it's all randomised. You're not picking out a group of people that are better than another group of people in terms of their symptoms. [Joyce]*<br><br>*I would have particularly liked it if I was one of the people that was offered sort of help with sleep and so on, because the ideal for me would be to find an alternative to running through the highs and lows of my life at three o'clock in the morning would be good. So I was a bit disappointed. [Arthur]*
**9. Collect data on patient-reported outcomes measures to assess data**		*Things affect you differently at different times […] some days, if you're feeling really well, I think you fill it in through rose-tinted glasses. That's the only way I can describe it. If you were having a really good day*

**completion rates and inform the selection of the primary outcome measure and sample size for a full trial**		*and things were going well, you'd fill it in a little bit differently … [Joyce]*
