# Supplementary material for: REST: a preoperative tailored sleep intervention for patients undergoing total knee replacement – feasibility study for a randomised controlled trial
Source: BMJ Open. 2024 Mar 20;14(3):e078785. doi: 10.1136/bmjopen-2023-078785 (PMC10961562; doi:10.1136/bmjopen-2023-078785)
Supplement: Author's manuscript [file bmjopen-2023-078785.draft_revisions.pdf]

BMJ Open is committed to open peer review. As part of this commitment we make the peer review history of every article we publish publicly available.

When an article is published we post the peer reviewers' comments and the authors' responses online. We also post the versions of the paper that were used during peer review. These are the versions that the peer review comments apply to.

The versions of the paper that follow are the versions that were submitted during the peer review process. They are not the versions of record or the final published versions. They should not be cited or distributed as the published version of this manuscript.

BMJ Open is an open access journal and the full, final, typeset and author-corrected version of record of the manuscript is available on our site with no access controls, subscription charges or pay-per-view fees (http://bmjopen.bmj.com).

If you have any questions on BMJ Open's open peer review process please email info.bmjopen@bmj.com

BMJ Open

REST: A pre-operative tailored sleep intervention for patients undergoing total knee replacement: feasibility study for a randomised controlled trial

Journal:	*BMJ Open*
Manuscript ID	bmjopen-2023-078785
Article Type:	Original research
Date Submitted by the Author:	11-Aug-2023

Complete List of Authors:	Bertram, Wendy; University of Bristol Medical School, Musculoskeletal Research Unit; NIHR Bristol Biomedical Research Centre<br>Penfold, Chris; University of Bristol Medical School, Musculoskeletal Research Unit<br>Rayment, Dane; North Bristol NHS Trust<br>White, Simon; Cardiff and Vale University Health Board<br>Wylde, Vikki; University of Bristol Medical School, Musculoskeletal Research Unit; NIHR Bristol Biomedical Research Centre<br>Glynn, Joel; University of Bristol Medical School<br>Johnson, Emma; University of Bristol Medical School<br>Burston, Amanda; University of Bristol Medical School, Musculoskeletal Research Unit<br>Howells, Nicholas; North Bristol NHS Trust<br>Gooberman-Hill, Rachael; University of Bristol Medical School; NIHR Bristol Biomedical Research Centre<br>Whale, Katie; University of Bristol Medical School, Musculoskeletal Research Unit; NIHR Bristol Biomedical Research Centre<br>Blom, Ashley; The University of Sheffield

Keywords:	Knee < ORTHOPAEDIC & TRAUMA SURGERY, SLEEP MEDICINE, Feasibility Studies

SCHOLARONE™
Manuscripts

I, the Submitting Author has the right to grant and does grant on behalf of all authors of the Work (as defined in the below author licence), an exclusive licence and/or a non-exclusive licence for contributions from authors who are: i) UK Crown employees; ii) where BMJ has agreed a CC-BY licence shall apply, and/or iii) in accordance with the terms applicable for US Federal Government officers or employees acting as part of their official duties; on a worldwide, perpetual, irrevocable, royalty-free basis to BMJ Publishing Group Ltd ("BMJ") its licensees and where the relevant Journal is co-owned by BMJ to the co-owners of the Journal, to publish the Work in this journal and any other BMJ products and to exploit all rights, as set out in our licence.

The Submitting Author accepts and understands that any supply made under these terms is made by BMJ to the Submitting Author unless you are acting as an employee on behalf of your employer or a postgraduate student of an affiliated institution which is paying any applicable article publishing charge ("APC") for Open Access articles. Where the Submitting Author wishes to make the Work available on an Open Access basis (and intends to pay the relevant APC), the terms of reuse of such Open Access shall be governed by a Creative Commons licence – details of these licences and which Creative Commons licence will apply to this Work are set out in our licence referred to above.

Other than as permitted in any relevant BMJ Author's Self Archiving Policies, I confirm this Work has not been accepted for publication elsewhere, is not being considered for publication elsewhere and does not duplicate material already published. I confirm all authors consent to publication of this Work and authorise the granting of this licence.

For peer review only

Title

REST: A pre-operative tailored sleep intervention for patients undergoing total knee replacement: feasibility study for a randomised controlled trial

Target Journal: BMJ Open BMJ Open submission guidelines

Authors

Wendy Bertram[1,2], Chris Penfold[1], Joel Glynn[1], Emma Johnson[1], Amanda Burston[1], Dane Rayment[3], Nick Howells[3], Simon White[4], Vikki Wylde[1,2], Rachael Gooberman-Hill[1,2], Ashley Blom[5], Katie Whale[1,2].

Corresponding Author: Wendy Bertram, Wendy.Bertram@bristol.ac.uk

Affiliations

1. University of Bristol
2. NIHR Bristol Biomedical Research Centre, University Hospitals Bristol and Weston NHS Foundation Trust and University of Bristol
3. North Bristol NHS Trust
4. Cardiff and Vale University Health Board
5. University of Sheffield

Abstract

Objectives

To test the feasibility of a randomised controlled trial of a novel pre-operative tailored sleep intervention for patients undergoing total knee replacement.

Methods

This study was a feasibility two-arm two-centre randomised controlled trial using 1:1 randomisation with an embedded qualitative study. Pre-operative patients identified from waiting lists at two NHS hospitals were screened by postal questionnaire for disturbed sleep, which was defined as a score of 0-28 on the Sleep Condition Indicator questionnaire. Participants allocated to the intervention received the REST intervention: a pre-operative tailored sleep assessment and behavioural intervention package delivered by an Extended Scope Practitioner (ESP), with a follow-up phone call four-weeks post-intervention. All participants received usual care as provided by the participating NHS hospitals. Patient reported outcomes were assessed at baseline, one-week pre-surgery, and 3-months post-surgery. Data collected to determine feasibility included the number of eligible patients, recruitment rates, and intervention adherence. Qualitative work explored the acceptability of the study processes and intervention delivery through interviews with ESPs and patients.

Results

Screening packs were posted to 378 patients and 57 patients were randomised. Of those randomised, 20 had surgery within the study timelines. An appointment was attended by 25/28 (89%) of participants randomised to the intervention. Follow-up outcomes measures were completed by 40/57 (70%) of participants pre-surgery and 15/57 (26%) post-surgery. Where outcome measures were completed, data completion rates were 80% or higher for outcomes at all timepoints, apart from the painDETECT: 86% complete at baseline, 72% at pre-surgery and 67% post-surgery. Interviews indicated that most participants found the study processes and intervention acceptable.

Conclusions

This feasibility study has demonstrated that with some amendments to processes and design, an RCT to evaluate the clinical and cost-effectiveness of the REST intervention is feasible.

For peer review only - http://bmjopen.bmj.com/site/about/guidelines.xhtml

Article Summary: Strengths and limitations of this study

- COVID restrictions in place during delivery required study procedures to be redesigned to enable remote data collection. These restrictions also influenced the volume of knee replacement operations performed, which is reflected in the number of participants who received an operation within the study period. Despite this the trial was feasible, which is encouraging.
- Data collection at the 3-month post-surgery timepoint was limited due to operation dates and volume of operations performed during the study.
- This study was undertaken in two National Health Services hospitals, which demonstrates that it is feasible to undertake a full trial in these settings, however the findings may not necessarily be generalisable to other settings.
- Embedded qualitative work explored acceptability of the intervention and of the trial processes. These results provide important insight into final study design to support acceptability and participant engagement with a full trial.

For peer review only

Background

Over 100,000 total knee replacements (TKR) are performed yearly in the UK.[1,2] The primary reason for surgery is severe chronic pain and functional limitation due to osteoarthritis. Sleep issues are a substantial problem for people awaiting joint replacement; 60-75% of people with osteoarthritis and 70% of patients awaiting joint replacement experience sleep problems, which increase with condition severity.[3-6] Patients report issues with sleep onset and maintenance, and middle of the night waking.[7]

Poor sleep causes worsening joint pain, depressive symptoms, lower physical activity, and increased risk of cardiovascular and pulmonary disease in patients with osteoarthritis.[8,9] Poor sleep can negatively impact surgical recovery causing slower wound healing, impaired immune function, increased risk of infection, and longer hospital stays.[10] Poor sleep before TKR is associated with increased acute and chronic post-surgical pain, increased analgesic use, reduced joint function and range of motion, lower satisfaction, and longer inpatient stays.[6,11,12]

Previous trials on sleep and joint replacement have predominantly focused on peri- and post-operative pharmacological interventions.[13-15] A recent systematic review identified that improved pre-operative sleep reduced pain levels and analgesic consumption after TKR.[16] Guidance from NICE and EULAR advises avoidance of pharmacological therapy for long-term management of sleep issues and recommends behavioural approaches as first-line treatment.[17,18] Non-pharmacological sleep interventions are potentially more sustainable and cost-effective, with lower risk of side-effects.[19] Our recent systematic review identified no RCTs evaluating a non-pharmacological intervention targeting sleep in patients waiting for TKR.[20]

This study aimed to evaluate the feasibility of conducting an RCT to evaluate the clinical and cost-effectiveness of REST, a non-pharmacological complex sleep intervention for patients undergoing TKR.

Methods

Study design

REST is a two-centred randomised controlled feasibility trial with 1:1 randomisation and an embedded qualitative study. Participants were recruited from two secondary care NHS hospitals in England and Wales. Ethics approval was obtained from the South West Cornwall & Plymouth Research Ethics Committee (20/SW/0189). The trial was prospectively registered (ISRCTN14233189). The CONSORT checklist is provided in appendix 1.

Patient and public involvement (PPI)

The study was developed in collaboration with a musculoskeletal PPI group working in partnership with the University of Bristol and North Bristol NHS Trust. The study benefited from the active involvement and contributions from a group of experienced patient partners. The group comprised five patients with lived experience of knee replacement. They met four times to co-design patient-facing study materials, monitor study progress, provide input into study process, and review the study results and dissemination plans. A patient partner was a member of the Steering committee.

For peer review only - http://bmjopen.bmj.com/site/about/guidelines.xhtml

Participant identification, recruitment and randomisation

Patients waiting for a primary TKR for osteoarthritis were identified from surgical waiting lists. Those likely to have surgery within three months were sent a pre-notification card by post followed by a screening questionnaire. The three-month preoperative timepoint was selected to allow sufficient time for intervention delivery and engagement to affect behaviour change.

Eligibility criteria were: adults on the TKR waiting list, experiencing disturbed sleep (defined as a score of 0-28 on the Sleep Condition Indicator (SCI) questionnaire[21]), and access to a device with internet connection. Exclusion criteria were: diagnosed with or receiving treatment for a clinical sleep disorder, taking prescription medication to help with sleep, having taken part in an interventional sleep study in the past six months, and unable or unwilling to attend an intervention appointment, provide informed consent or complete questionnaires in English. Patients who returned an eligible screening questionnaire were telephoned for an eligibility assessment. Eligible patients were invited to a recruitment visit. Patients not eligible at screening were sent a thank you letter.

After written informed consent was provided and the baseline questionnaire completed, participants were randomly allocated to the intervention plus usual care or usual care alone. Randomisation was conducted on a 1:1 intervention:control basis by the co-ordinating centre using computer generated randomisation. Participants, practitioners, and research staff were not blinded.

Intervention

The REST intervention was developed following Medical Research Council guidance for complex intervention development.[22,23] REST consists of an appointment with an Extended Scope Practitioner (ESP) delivered via videoconference or telephone three months before surgery. The appointment comprised a comprehensive sleep assessment to identify individual sleep issues and needs, and an assessment of sleep apnoea risk. Participants scoring high risk for sleep apnoea were referred to their GP in addition to the intervention. Participants were then provided with tailored sleep education and sleep hygiene advice. One of three existing evidence-based sleep interventions (ESIs) were allocated through a shared decision-making process: cognitive-behavioural therapy for insomnia (delivered via online platform Sleepstation), relaxation (delivered via the Calm app, workbook, or guided audio/video), and mindfulness (delivered via the Headspace app, workbook, or guided audio/video).

Participants were provided with a personalised sleep plan including SMART goals based on the sleep hygiene recommendations, a detailed overview of their chosen ESI, instructions for use and digital access (if applicable) and any materials. Participants received a follow-up call four weeks after the appointment to review progress and engagement with their sleep plan. This included addressing any barriers experienced, review of the sleep goals, and adjustments to the sleep plan if needed.

All participants received usual care as provided by the participating NHS hospitals. Safety reporting was exclusively for adverse reactions directly attributable to the intervention.

Intervention delivery training

All practitioners took part in a one-day online intervention delivery training session. This covered the study background, evidence on the relationship between sleep and pain, an overview of each ESI, and practical guidance on delivery. The Chief Investigator communicated regularly with practitioners to provide further support and training

For peer review only - http://bmjopen.bmj.com/site/about/guidelines.xhtml

if required. ESPs were provided with a detailed intervention manual, which provided guidance and proformas for conducting the sleep assessment, sleep hygiene and education advice, information on each ESI, participant sleep plan, and post-appointment tasks.

Intervention timing

REST was designed to be delivered 3-months pre-surgery. This timepoint was chosen to optimise the effect of the sleep interventions, because of the duration of the sleep interventions (Sleepstation is delivered over 6-8 weeks), and theories of behaviour change maintenance[24].

Intervention delivery fidelity

Non-participatory observations were conducted to assess the degree to which the intervention was delivered as intended as per the intervention manual. One clinic appointment and follow-up call were observed for each ESP. Observations were conducted independently by two members of the research team. Participants were asked to provide verbal consent for the researcher to be present during their clinic appointment.

Feasibility outcomes

Feasibility outcomes included recruitment rate, intervention uptake and adherence, outcome data completion, and intervention acceptability. A full list of outcomes and measurements are outlined in Table 1.

Patient-reported outcomes

Patient-reported outcomes were assessed prior to randomisation (approximately 3 months pre-operative), one week prior to surgery and three months after surgery. Participants who did not have their operation by six-months post-randomisation completed pre-surgery outcomes.

Outcomes included joint pain (Oxford Knee Score (OKS)[25]), neuropathic pain (painDETECT[26]), sleep quality and beliefs about sleep (Sleep Condition Indicator (SCI[21]), Pittsburgh Sleep Quality Index (PSQI)[27]), mental wellbeing (Hospital Anxiety and Depression Scale (HADS)[28]), and general health and wellbeing (EQ-5D-5L[29],ICECAP-A[30]). Health resource use data included health care interactions in the community and secondary care, including medication use, and were collected in the three-month pre-operative and post-operative questionnaires only. Intervention participants completed sleep treatment engagement questions at the 3-month post-surgery timepoint.

Qualitative study

Embedded qualitative work explored the acceptability of the intervention and study processes. Interviews were conducted with participants in both arms and with ESPs delivering the intervention. Participants who expressed an interest at enrolment in being interviewed were sent an invitation letter, reply slip, and pre-paid envelope. Participants who returned the reply slip were contacted a researcher to discuss participation and arrange an interview for those interested. Informed consent was provided by participants before interview. ESPs were invited to participate in two interviews: one following the intervention training day and one after delivery of intervention appointments. Informed written or recorded verbal consent was provided by all ESPs.

Qualitative data collection

All interviews were conducted via videoconference or telephone depending on preference. Face-to-face interviews were not possible due to COVID-19 restrictions. Participant interviews were guided by a semi-structured topic guide

For peer review only - http://bmjopen.bmj.com/site/about/guidelines.xhtml

covering design and conduct of the trial (all participants), experiences of the intervention and views on impact (intervention group), and changes to made to sleep (all participants). ESP interviews at both timepoints explored acceptability of training and intervention delivery.

Progression criteria

Progression criteria for demonstrating the feasibility of a RCT were proposed as ≥60 patients randomised (75% of target) and 75% uptake of the intervention. Uptake was defined as the number of participants who attended an intervention appointment. Criteria for progression based on acceptability were:

- All participants: expressed comfort with study processes including recruitment, randomisation, outcome measures and follow-up.
- Intervention participants: level of engagement with clinic processes, adherence to and engagement with the intervention.

Sample size

The target sample was 80 participants (40 intervention, 40 usual care) to estimate 75% randomisation rate (RCT progression criteria) with 95% confidence interval from 65% to 85%, and to estimate 75% intervention uptake with 95% confidence interval from 60% to 90%.

Statistical analyses

Baseline characteristics of each group were tabulated using means and standard deviations for normally distributed data, medians and interquartile ranges for non-normally distributed data, and percentages and counts for categorical data. Patient-reported outcome measures were summarised descriptively. The proportion of people without complete responses for the outcome questionnaires were reported at each timepoint with commentary on any patterns of missing data between timepoints. Outcome data tables are provided in appendix 2.

Economic analyses

The feasibility of collecting data for an economic evaluation alongside a full trial was assessed, including intervention costs for appointments and tailored intervention. Economic analysis tables are provided in appendix 2.

Qualitative analysis

Data collection and analysis were conducted in parallel after the first three interviews. Audio files were transcribed, then transcripts were anonymised and imported into the qualitative software package NVivo10. Participant and ESP data were analysed separately using Framework Analysis, a thematic approach that enables structured comparison and contrast of data across cases.[31] Data were organised using the topic guide as a starting framework. Five transcripts were independently double coded and discussed within the team to offer further insight into interpretation and to enhance rigour through different approaches and knowledge.[32] All participants were assigned pseudonyms to ensure anonymity. Participant demographics and supporting quotes are included in appendices 3 and 4.

For peer review only

Results

1. Eligibility and recruitment rates

Between March and December 2021, 378 patients were invited to take part in screening. Of these, 258 (68%) returned completed screening questionnaires: 146 were willing to take part and met screening eligibility criteria, 58 declined to take part and 54 were not eligible. Reasons for ineligibility included: SCI score ≥29 (28/54, 52%), having a sleep disorder or taking medication to help with sleep (18/54, 33%), operation dates allocated in the near future or surgery being postponed (4/54, 7%), questionnaire returned after study closure (3/54, 6%), and no internet access (1/54, 2%).

Telephone calls were made to the 146 patients who returned an eligible screening questionnaire: 8 were not contactable, 32 were ineligible, 47 declined to take part and 59 consented to take part. Reasons for ineligibility at telephone screening included operation dates allocated in the near future or surgery being postponed (13/32, 41%), having a sleep disorder or taking medication to help with sleep (9/32, 28%), no internet access (7/32, 22%), already had surgery (2/32, 6%), and did not speak English (1/32, 3%). Most did not give a reason for declining taking part. Where given, common reasons for declining were time commitments or personal circumstances (17/47, 36%) and did not feel they had a sleep problem or did not think treatment would help (6/47, 13%).

Two participants withdrew prior to randomisation; therefore 57 participants were randomised.

A CONSORT diagram outlining participant flow is provided in Figure 1. Baseline participant characteristics are provided in Table 2. Patients who were eligible but did not participate had higher (better) preoperative sleep as measured using the SCI than randomised participants (appendices).

2. Attendance at the intervention clinic appointment

Of the 28 participants assigned to the intervention group, 3 withdrew before receiving the intervention due to operation dates being allocated in the near future (10.7%), 25 attended the clinic appointment (89.3%), and 15 completed the 4-week follow-up call (53.6%).

3. Engagement with the REST intervention and adherence to the agreed sleep plan

Qualitative study: participant interviews

38 trial participants were invited to take part in an interview (N=18 Bristol/20 Cardiff; 22 intervention, 16 usual care). Sixteen expressions of interest were received, and 13 participants were interviewed (N= 10 Bristol/3 Cardiff; N= 8 intervention/5 usual care..

Participants were willing to discuss their sleep issues with the ESPs. Factors influencing readiness to engage in a conversation with the practitioner included a belief that they were meeting with a knowledgeable and skilled professional. The practitioner’s manner and communication style helped to create a safe space that enabled participants to feel both at ease and comfortable to open up about their experiences. Participants who recalled the shared decision-making process of choosing a sleep intervention said that they felt involved and informed.

4. Intervention acceptability

For peer review only - http://bmjopen.bmj.com/site/about/guidelines.xhtml

Patient acceptability

COVID-19 restrictions at the time of delivery required all recruitment and intervention appointments to be conducted remotely by videoconference or telephone. Participants understood the need for remote appointments, but confidence and familiarity with this approach varied. For many participants confidence in using video calls had grown during the pandemic. One participant struggled with confidence and familiarity with online appointments and needed additional support. Remote delivery was generally seen as acceptable. Participants highlighted benefits of removed travel and cost, and reduced risk of COVID-19 and other infections.

Appointment length and structure were considered appropriate. Participants felt they had enough time to ask questions, discuss what was being asked of them, and to address concerns. The time to attend the appointment was seen as worthwhile, as it gave access a practitioner who provided the chance to talk about their problems and a focus during their wait for surgery.

Due to clinic delays and competing demands of the practitioners, some appointments started later than planned. Although some participants were accepting of this, one participant reported feeling frustrated and angry at the inconvenience caused.

No adverse events were reported.

Practitioner acceptability

ESP acceptability of intervention delivery was high. ESPs were able to deliver most appointments using videoconference which supported better communication with participants, however one ESP expressed a preference for telephone appointments as this required less set up and had greater flexibility. Some technical issues were raised due to ESPs using different NHS computers for appointments depending on their schedule. This caused problems with webcam connectivity and added additional time to appointment set up. Overall paperwork was straightforward to complete with the questions and proformas clear and easy to use. Some aspects of the assessment were viewed as repetitive and could be shortened to give more time for discussion with participants.

5. Intervention delivery

Intervention fidelity assessments included observation of at least one clinic appointment and one follow-up appointment for each practitioner. Practitioners fully or partially met all areas of adherence (fidelity to the intervention as described in the manual) and compliance (proficiency of delivery) during the intervention appointment. Three areas for improvement in training and delivery were identified: educating the participant about sleep and TKR, setting SMART sleep goals, and shared-decision-making discussions around recommended sleep interventions.

6. Acceptability of randomisation

Qualitative interviews with participants found that most participants found randomisation acceptable and understood the need for this design. Some expressed disappointment on receiving usual care. Disappointment stemmed from their desire to benefit from the intervention because of struggles with sleep.

7. Feasibility and acceptability of collecting health economic data

For peer review only - http://bmjopen.bmj.com/site/about/guidelines.xhtml

Quality of life measures

EQ-5D-5L and ICECAP mean scores and standard deviations with response rates at each of the time point are presented in the appendices. EQ-5D-5L utility scores were calculated using Hernández Alava et al. 2020 method as recommended by NICE PMG36.[33] No evidence was found of ceiling of floor effects for either quality of life measure, and the measures seem to be responsive to quality of life changes in this population.

Resource use

Responses to the bespoke resource use questionnaire were 40/57 (70%) pre-operatively and 14/27 (52%) post-operatively. Changes and clarification of questions will be made based on responses to individual questions. For example, more options are needed for physiotherapy appointments as many responses were selected “other”. Most resource use and cost fell on NHS services including GP, outpatient, and physiotherapy appointments. Overall, the estimated cost (NHS perspective, excluding intervention costs) was £507 and £688 for intervention and usual care arms respectively. However, no interpretation of this difference can be made given the small number of participants in this feasibility study.

Intervention costs

Given we do not know the value or mechanism of payments from NHS to Sleepstation for use of the app, nor the number of patients purchasing app subscriptions to Calm and Headspace, we costed the intervention based on several assumptions (appendices). We have generated a minimum, mean, and maximum expected intervention cost. The mean cost of the intervention was estimated at £134.45 per person (£141.04 including patient out of pocket costs) based on the mean clinic, preparation, and post-clinic time captured in the feasibility study, an NHS cost of £147 per patient for the use of Sleepstation (based on a 50% discount on publicly advertised cost), and 1/3 of those using Calm or Headspace upgrading to paid subscriptions for additional access. The max estimated at £295.73 and minimum cost £45.29.

8. Optimisation of the intervention delivery training package

Four ESPs took part in an interview at timepoint one (post-training) and three at time-point two (post-delivery).

Training was delivered as a one-day online course. Practitioners spoke positively about their experience of the training, praising the organisation and focus. Practitioners appreciated receiving information about the rationale behind the intervention. Some felt the level of detail around sleep science could be reduced, but still found this interesting. They were given sufficient time to ask questions, however the remote format made this slightly harder compared to face-to-face. Following experience of intervention delivery, ESPs reported challenges in setting SMART goals including what areas to focus on and the level of detail needed. They suggested more training and knowledge of each ESI would be beneficial and facilitate better shared decision-making discussions with participants. A small number of participants attending appointments felt they did not have a sleep issue. This made it challenging for ESPs to follow the intervention handbook.

Areas for improving practitioner training were identified as:

- Increased time for role-play and practical exercises
- Additional information on sleep interventions to increase understanding and familiarity
- Further training and practical exercises on setting SMART goals

For peer review only - http://bmjopen.bmj.com/site/about/guidelines.xhtml

- Advice and guidance on how to support participants who do not believe they have a sleep issue or are not motivated to make changes
- Additional supervision meetings throughout intervention delivery to provide further support, answer questions, and address challenges

9. Data completion rates, selection of the primary outcome measure and sample size for a full trial

Data completion rates

Data completion rates are provided in Table 3. All participants completed the baseline OKS and EQ5D-5L. Completion rates were consistently lower for the PSQI (86%-93%) at baseline and pre-operative timepoints. The painDETECT questionnaire had the lowest completion rates at each timepoint, ranging from 60-86%.

Qualitative interviews demonstrated that questionnaire completion was acceptable overall.

Patient-reported outcome measures

Baseline, pre-operative, and post-operative outcome measures are presented Table 4. The purpose of this study was to evaluate the feasibility of conducting an RCT, therefore statistical tests to compare outcomes between treatment arms were not performed.

Participants randomised to the intervention group reported an improvement in average Pittsburgh Sleep Quality Index score from 12.0 (95% CI 8.8, 14.2) at baseline to 8.0 (95% CI 6, 11) at the end of the intervention (12 weeks after randomisation), compared to no change in the usual care group (baseline score of 11 (95% CI 8, 13.5) and 12-week score of 11 (95% CI 7, 13)).

A proposed primary outcome was pain after surgery as measured by the OKS pain component[34]. The target timing for randomisation was three months pre-operative. The mean number of days from randomisation to operation was 118 days, with 35% (n=20) of participants having surgery during the six-month participation window. Outside of the participation window, a further 18 participants were allocated an operation date. The remaining 21 had not been allocated a surgery date at study closure.

<u>Discussion</u>

This feasibility study has demonstrated that the REST intervention is acceptable to patients and clinicians. With modifications, a full trial is feasible. Criteria for progression to a full trial are ≥60 patients randomised (75% of target) and 75% uptake of the intervention. More than 75% of participants allocated the intervention attended the clinic appointment (89%, n = 25/29). We randomised 57 patients during a period of COVID-19 restrictions when many studies were unable to recruit. In addition, removing the need to screen patients who are three months prior to surgery would facilitate increased recruitment.

Strengths

Despite COVID-19 restrictions at the time of the study, screening and recruitment procedures were successful and 57 participants were randomised. Close working with waiting list staff and surgeons was essential to understanding which patients were most likely to be allocated a surgery date in three months. Once identified, the return rate for

For peer review only - http://bmjopen.bmj.com/site/about/guidelines.xhtml

screening questionnaires was 68%. Evidence-based methods to increase the return of postal questionnaires were used, including pre-notification cards and non-monetary incentives (individually wrapped tea bags).

Most participants randomised to the intervention group attended an appointment (89%, n = 25/29) and engaged with treatment. Remote delivery of the intervention was viewed positively by participants. Those who had a video call who appreciated being able to see the practitioner, welcoming the human connection and chance to build rapport.

Limitations

Although intervention uptake and engagement with treatment was good, inequalities in access to the internet and electronic devices are an issue in studies that use remote delivery. Some participants experienced delays in obtaining appointment times, which varied by practitioner and site; this may be solved by centralised intervention delivery and offering options such as telephone delivery for those without internet access.

Patients who were eligible at screening but who chose not to take part in the study had better preoperative sleep as measured using the SCI than randomised participants. A common reason for not taking part was not having a sleep problem or feeling that treatment would not help. This indicates the SCI eligibility score cutoff would benefit from being lowered.

Study delivery was redesigned to be conducted entirely remotely to meet COVID-19 restrictions, which also influenced the volume of knee replacement operations performed, affecting the number of participants undergoing surgery within the study.

Generally, NHS operation dates cannot be reliably predicted three months in advance, therefore identifying patients at this timepoint proved challenging. Completion of the primary outcome at three months post-surgery was also difficult because many participants did not have their operation within the study timelines. In addition, variations in length of time from randomisation to three months post-surgery would result in high heterogeneity. To address this, the primary outcome assessment for a full trial should not be the proposed outcome of pain three months post-operative, but sleep quality at 14 weeks post-randomisation timepoint for generalisability.

Modifications

There are several key areas to adapt and improve for a future full trial. These include changes to the clinician training programme, including more detailed training on existing sleep interventions and setting SMART goals, streamlined delivery of the intervention by provision of an online portal, and lowering to the screening cut-off for the SCI score.

A review of equality, diversity, and inclusion strategies will ensure a full trial supports inclusivity and engagement from a wide range of communities.

Conclusions

We have demonstrated that a full RCT is feasible based on the predefined progression criteria and have identified areas for improvement to optimise trial design. Recruitment is achievable, engagement with and adherence to the intervention is high and, importantly, the intervention is acceptable to patients and clinicians.

For peer review only - http://bmjopen.bmj.com/site/about/guidelines.xhtml

Footnotes

Funding

This study is funded by the National Institute for Health and Care Research (NIHR) (Research for Patient Benefit; grant reference number NIHR201036). The research team acknowledges the support of the NIHR, through the Clinical Research Network. This work was also supported by the NIHR Biomedical Research Centre at University Hospitals Bristol and Weston NHS Foundation Trust and the University of Bristol. The views expressed are those of the authors and not necessarily those of the NIHR or the Department of Health and Social Care.

Competing interests: None declared.

Author contributions

KW was Chief Investigator. KW, RGH, VW, CP, AB, AWB, DR, NH, SW and JG were co-applicants on the grant application to NIHR. All authors were involved in the design, delivery and interpretation of the study. WB was the Trial Manager. JG was responsible for the economic analysis. CP was responsible for the statistical analysis. EJ and KW were responsible for the qualitative analysis. WB, KW, CP and JG have access and/or verified the underlying data. WB drafted the manuscript; all authors revised if for important content and approve the final manuscript.

Data sharing

Participants were asked on the consent form if they were willing for their information to be shared anonymously with other researchers to support other research in the future. Anonymised data will be stored on the University of Bristol Research Data Storage Facility (https://data.bris.ac.uk) and will be shared via the University of Bristol Research Data Repository within six months of the publication of the study results. Access to the data will be restricted to ensure that data is only made available to bona fide researchers after a Data Access Agreement has been signed by an institutional signatory.

Ethics

Patient consent for publication: Not applicable

Ethics approval

This study received a favourable opinion from the South West – Cornwall & Plymouth Research Ethics Committee, reference 20/SW/0189, and approval from the Health Research Authority and Health and Care Research Wales (IRAS 289761). Participants gave informed consent to participate in the study before taking part.

Acknowledgements

For peer review only

We would like to thank our patient partners for their help and input, and the research team members and health care professionals who delivered the study including: Gemma Munkenbeck, Leigh Morrison, Paolo Buscemi, Christine Hobson, Giles Head, Lucy Young, Jessica Falatoori, Mat Williams, Chris Dobson and Mark Brand.

The research team acknowledges the support of the NIHR, through the Clinical Research Network. This work was also supported by the NIHR Biomedical Research Centre at University Hospitals Bristol and Weston NHS Foundation Trust and the University of Bristol. The views expressed are those of the authors and not necessarily those of the NIHR or the Department of Health and Social Care.

For peer review only

For peer review only - http://bmjopen.bmj.com/site/about/guidelines.xhtml

References

1. Registry NJ. National Joint Registry 15th Annual Report 2018.
2. Project SA. Annual Report 2018. NHS National Services Scotland; 2018 2018.
3. Hawker GA, et al. The multidimensionality of sleep quality and its relationship to fatigue in older adults with painful osteoarthritis. Osteoarthritis Cartilage. 2010;18(11):1365-71.
4. Wilcox S, et al. Factors related to sleep disturbance in older adults experiencing knee pain or knee pain with radiographic evidence of knee osteoarthritis. J Am Geriatr Soc. 2000;48(10):1241-51.
5. Manning BT, et al. Prospective Assessment of Sleep Quality Before and After Primary Total Joint Replacement. Orthopedics. 2017;40(4):e636-e40.
6. Bjurstrom MF, et al. Preoperative sleep quality and adverse pain outcomes after total hip arthroplasty. Eur J Pain. 2021;25(7):1482-92.
7. Smith TO, et al. Changes in social isolation and loneliness following total hip and knee arthroplasty: longitudinal analysis of the English Longitudinal Study of Ageing (ELSA) cohort. Osteoarthritis Cartilage. 2017;25(9):1414-9.
8. Dai Z, et al. Sleep Quality Is Related to Worsening Knee Pain in Those with Widespread Pain: The Multicenter Osteoarthritis Study. J Rheumatol. 2020;47(7):1019-25.
9. Song J, et al. Sleep Disturbance Trajectories in Osteoarthritis. J Clin Rheumatol. 2021;27(8):e440-e5.
10. Wright KP, Jr., et al. Influence of sleep deprivation and circadian misalignment on cortisol, inflammatory markers, and cytokine balance. Brain Behav Immun. 2015;47:24-34.
11. Van Meirhaeghe JP, et al. Improvement in Sleep Patterns After Hip and Knee Arthroplasty: A Prospective Study in 780 Patients. J Arthroplasty. 2021;36(2):442-8.
12. Ding Z, et al. Preoperative High Sleep Quality Predicts Further Decrease in Length of Stay after Total Joint Arthroplasty under Enhanced Recovery Short-stay Program: Experience in 604 Patients from a Single Team. Orthop Surg. 2022;14(9):1989-97.
13. Gong L, et al. Sleep Quality Effects Recovery After Total Knee Arthroplasty (TKA)--A Randomized, Double-Blind, Controlled Study. J Arthroplasty. 2015;30(11):1897-901.
14. Kirksey MA, et al. Impact of Melatonin on Sleep and Pain After Total Knee Arthroplasty Under Regional Anesthesia With Sedation: A Double-Blind, Randomized, Placebo-Controlled Pilot Study. J Arthroplasty. 2015;30(12):2370-5.
15. Krenk L, et al. Postoperative sleep disturbances after zolpidem treatment in fast-track hip and knee replacement. J Clin Sleep Med. 2014;10(3):321-6.
16. Shen SP, et al. Improved Perioperative Sleep Quality or Quantity Reduces Pain after Total Hip or Knee Arthroplasty: A Systematic Review and Meta-Analysis. Orthop Surg. 2021;13(4):1389-97.
17. Excellence NIfHCa. Insomnia: Scenario: Managing long-term insomnia (more than 3 months duration). 2022.
18. Geenen R, et al. EULAR recommendations for the health professional's approach to pain management in inflammatory arthritis and osteoarthritis. Ann Rheum Dis. 2018;77(6):797-807.
19. Koffel E, et al. Improving pain and sleep in middle-aged and older adults: the promise of behavioral sleep interventions. Pain. 2019;160(3):529-34.
20. Whale K, et al. The effectiveness of non-pharmacological sleep interventions for people with chronic pain: a systematic review and meta-analysis. BMC Musculoskeletal Disorders. 2022;In Press.
21. Espie CA, et al. The Sleep Condition Indicator: reference values derived from a sample of 200 000 adults. J Sleep Res. 2018;27(3):e12643.
22. Craig P, et al. Developing and evaluating complex interventions: the new Medical Research Council guidance. Int J Nurs Stud. 2013;50(5):587-92.
23. Whale K, Gooberman-Hill R. Development of a novel intervention to improve sleep and pain in patients undergoing total knee replacement. Trials. 2022;23(1):625.
24. Kwasnicka D, et al. Theoretical explanations for maintenance of behaviour change: a systematic review of behaviour theories. Health Psychol Rev. 2016;10(3):277-96.
25. Dawson J, et al. Questionnaire on the perceptions of patients about total knee replacement. J Bone Joint Surg Br. 1998;80(1):63-9.
26. Freynhagen R, et al. painDETECT: a new screening questionnaire to identify neuropathic components in patients with back pain. Current medical research and opinion. 2006;22(10):1911-20.
27. Buysse DJ, et al. The Pittsburgh Sleep Quality Index: a new instrument for psychiatric practice and research. Psychiatry Res. 1989;28(2):193-213.

For peer review only - http://bmjopen.bmj.com/site/about/guidelines.xhtml

28. Zigmond AS, Snaith RP. The hospital anxiety and depression scale. Acta Psychiatr Scand. 1983;67(6):361-70.
29. Herdman M, et al. Development and preliminary testing of the new five-level version of EQ-5D (EQ-5D-5L). Qual Life Res. 2011;20(10):1727-36.
30. Al-Janabi H, et al. Development of a self-report measure of capability wellbeing for adults: the ICECAP-A. Qual Life Res. 2012;21(1):167-76.
31. Gale NK, et al. Using the framework method for the analysis of qualitative data in multi-disciplinary health research. BMC Med Res Methodol. 2013;13:117.
32. Tracy SJ. Qualitative Quality: Eight “Big-Tent” Criteria for Excellent Qualitative Research. Qualitative Inquiry. 2010;16(10):837-51.
33. Hernandez Alava M, et al. The EQ-5D-5L Value Set for England: Findings of a Quality Assurance Program. Value Health. 2020;23(5):642-8.
34. Pinedo-Villanueva R, et al. Identifying individuals with chronic pain after knee replacement: a population-cohort, cluster-analysis of Oxford knee scores in 128,145 patients from the English National Health Service. BMC Musculoskelet Disord. 2018;19(1):354.

For peer review only

Figure 1: CONSORT participant flow diagram

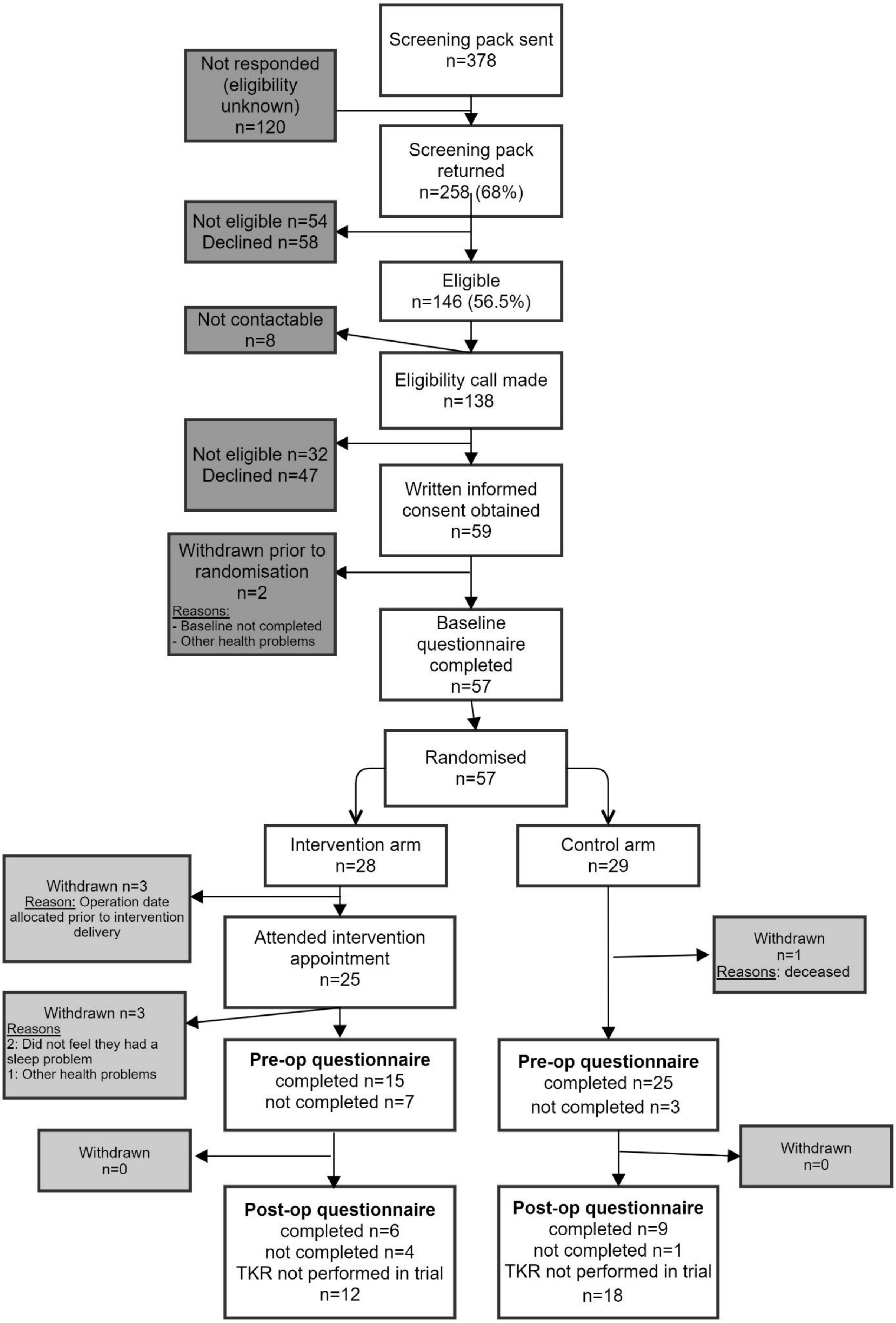

Table 1: Feasibility outcomes

Objective(s)	Outcome	Measurement
1	Eligibility and recruitment rates	Number of patients invited, returning screening questionnaires, eligible, consented and randomised. Retention rates.
2	Intervention uptake	Number of participants who attend the clinic appointment
3	Intervention adherence	Open ended questions at 4-week follow-up telephone call: • Changes made as a result of sleep hygiene and education advice • Engagement in the assigned sleep intervention • Any additional changes made to sleep or sleep routine
4 & 6	Participant interviews: acceptability of the intervention and randomisation	Semi-structured qualitative interviews with participants in the intervention group (n=20, 10 per site), and the control group (n=5).
4 & 8	Extended scope practitioner interviews: acceptability of the intervention and training optimisation	Semi-structured qualitative interviews with ESPs (n=4) at two timepoints: 1) after completion of training and 2) after intervention delivery
5	Intervention delivery fidelity	Observation of one clinic appointment and follow-up call for each ESP to assess adherence and compliance.
7	Health economics data	Quality of life measures (EQ-5D-5L, ICECAP-A) and health care resource use (community and secondary care) as documented in the patient completed outcome measure booklets at one week pre-operative and three months post-operative.
8	Optimisation of intervention training	Non-participatory observations of the ESP training. Semi-structured interviews with all ESPs (n=4) at two time points, after completion of training and after delivering the intervention.
9	Inform the primary outcome measure for a full trial	Quantitative data analysis, proportion of participants in ongoing pain in each treatment arm at three months after surgery.

For peer review only

Table 2: Characteristics of study participants at baseline

Characteristic	N = 57[1]
**Gender**	
Man	23 (40%)
Woman	34 (60%)
**Ethnicity (cleaned)**	
Non white/non British	1 (1.8%)
Other/not answered	4 (7.0%)
White/British	52 (91%)
**Do you consume any alcohol?**	
No	26 (46%)
Yes	30 (54%)
Unknown	1
**Alcohol units/week (excl. non-drinkers)**	9 (3, 14)
Non-drinker/unknown	27
**Do you consume any coffee?**	
No	16 (28%)
Yes	41 (72%)
**Cups of coffee/week (excl. non-drinkers)**	12 (7, 20)
Non-drinker/unknown	16
**Smoking status**	
Current	2 (3.5%)
Former	24 (42%)
Never	31 (54%)
**Employment status**	
Employed	11 (19%)
Other	2 (3.5%)
Retired	44 (77%)
**Marital Status**	
Divorced	9 (16%)
Married/partner	36 (63%)
Single	2 (3.5%)
Widowed	10 (18%)
**Other conditions**	
No	18 (34%)
Yes (please state)	35 (66%)
Unknown	4
**Other condition(s): Other joint replacement**	
Yes	4 (100%)
Unknown	53
**Other condition(s): Pain in other joints**	
Yes	4 (100%)
Unknown	53
**Other condition(s): Arthritis (any)**	
Yes	29 (100%)
Unknown	28
**Other condition(s): Injury**	
Yes	3 (100%)
Unknown	54

[1]n (%)

only

Table 3: Data completion rates

	Baseline		Pre-operative		Post-operative	
Characteristic	Intervention, N = 28[1]	Control, N = 29[1]	Intervention, N = 15[1]	Control, N = 25[1]	Intervention, N = 6[1]	Control, N = 9[1]
**OKS**						
Complete	28 (100%)	29 (100%)	13 (87%)	25 (100%)	6 (100%)	8 (89%)
Missing			2 (13%)	0 (0%)	0 (0%)	1 (11%)
**PainDETECT**						
Complete	24 (86%)	25 (86%)	9 (60%)	20 (80%)	4 (67%)	6 (67%)
Missing	4 (14%)	4 (14%)	6 (40%)	5 (20%)	2 (33%)	3 (33%)
**SCI**						
Complete	27 (96%)	27 (93%)	15 (100%)	24 (96%)	6 (100%)	9 (100%)
Missing	1 (3.6%)	2 (6.9%)	0 (0%)	1 (4.0%)		
**HADS**						
Complete	27 (96%)	29 (100%)	12 (80%)	25 (100%)	6 (100%)	9 (100%)
Missing	1 (3.6%)	0 (0%)	3 (20%)	0 (0%)		
**PSQI**						
Complete	24 (86%)	27 (93%)	13 (87%)	21 (84%)	6 (100%)	9 (100%)
Missing	4 (14%)	2 (6.9%)	2 (13%)	4 (16%)		
**EQ5D-5L**						
Complete	28 (100%)	29 (100%)	15 (100%)	25 (100%)	6 (100%)	8 (89%)
Missing					0 (0%)	1 (11%)
**ICECAP**						
Complete	25 (89%)	29 (100%)	15 (100%)	25 (100%)	6 (100%)	9 (100%)
Missing	3 (11%)	0 (0%)				

[1]n (%)

For peer review only

Table 4: Patient reported outcome measures

	Baseline		Pre-operative		Post-operative	
	Intervention, N = 28[1]	Usual Care, N = 29[1]	Intervention, N = 15[1]	Usual Care, N = 25[1]	Intervention, N = 6[1]	Usual Care, N = 9[1]
**OKS pain subscale**	8.5 (6.0, 10.0)	9.0 (6.0, 11.0)	7.0 (6.0, 10.0)	8.0 (5.0, 12.0)	13.50 (11.50, 15.50)	14.50 (12.50, 16.00)
**OKS function subscale**	7.5 (3.8, 12.0)	9.0 (7.0, 12.0)	7.0 (5.0, 9.8)	8.0 (5.0, 11.0)	21.5 (19.5, 22.8)	22.5 (21.0, 24.0)
**OKS total score**	16 (12, 21)	18 (13, 22)	15 (11, 19)	16 (9, 22)	34 (32, 40)	36 (35, 39)
**PainDetect score**	18 (11, 21)	13 (9, 19)	11 (10, 17)	14 (10, 24)	7 (6, 12)	10 (6, 16)
**PainDetect score(categorised)**						
Ambiguous	6 (25%)	5 (20%)	3 (33%)	5 (25%)	0 (0%)	1 (17%)
Neuropathic likely	9 (38%)	8 (32%)	1 (11%)	7 (35%)	1 (25%)	1 (17%)
Nociceptive	9 (38%)	12 (48%)	5 (56%)	8 (40%)	3 (75%)	4 (67%)
**Sleep conditions indicator**	11 (8, 14)	13 (10, 16)	14.0 (11.0, 21.0)	15.0 (9.5, 18.0)	22 (16, 24)	24 (18, 27)
**HADS score**	13 (10, 22)	16 (12, 19)	12.5 (9.0, 19.0)	14.0 (9.0, 20.0)	9.0 (4.2, 13.0)	8.0 (6.0, 14.0)
**HADS score (categorised)**						
Abnormal	19 (70%)	22 (76%)	7 (58%)	17 (68%)	3 (50%)	3 (33%)
Borderline abnormal	5 (19%)	5 (17%)	3 (25%)	6 (24%)	0 (0%)	2 (22%)
Normal	3 (11%)	2 (6.9%)	2 (17%)	2 (8.0%)	3 (50%)	4 (44%)
**ICECAP**	0.85 (0.66, 0.92)	0.84 (0.70, 0.89)	0.84 (0.55, 0.90)	0.84 (0.67, 0.91)	0.92 (0.87, 0.98)	0.91 (0.89, 0.92)
**EQ-5D**	0.33 (0.16, 0.57)	0.54 (0.30, 0.70)	0.39 (0.22, 0.54)	0.45 (0.22, 0.60)	0.67 (0.60, 0.73)	0.76 (0.73, 0.77)
**PSQI global score**	12.0 (8.8, 14.2)	11.0 (8.0, 13.5)	8.0 (6.0, 11.0)	11.0 (7.0, 13.0)	9.5 (5.2, 11.5)	7.0 (5.0, 9.0)

[1]Median (IQR); n (%)

Appendix 1: CONSORT 2010 checklist of information to include when reporting a pilot or feasibility randomised trial in a journal

Item	Description	Reported on line number
Title	Identification of study as randomised pilot or feasibility trial	1
Authors *	Contact details for the corresponding author	9
Trial design	Description of pilot trial design (eg, parallel, cluster)	90
Methods		
Participants	Eligibility criteria for participants and the settings where the pilot trial was conducted	103
Interventions	Interventions intended for each group	122
Objective	Specific objectives of the pilot trial	160
Outcome	Prespecified assessment or measurement to address the pilot trial objectives	169, Table 1
Randomisation	How participants were allocated to interventions	118
Blinding (masking)	Whether or not participants, care givers, and those assessing the outcomes were blinded to group assignment	119
Results		
Numbers randomised	Number of participants screened and randomised to each group for the pilot trial objectives	228
Recruitment	Trial status	N/A
Numbers analysed	Number of participants analysed in each group for the pilot objectives	324, 368
Outcome	Results for the pilot objectives, including any expressions of uncertainty	Objective 1: 228<br>2: 250<br>3: 256<br>4: 271<br>5: 300<br>6: 309<br>7: 315<br>8: 342<br>9: 366
Harms	Important adverse events or side effects	292
Conclusions	General interpretation of the results of pilot trial and their implications for the future definitive trial	448
Trial registration	Registration number for pilot trial and name of trial register	92
Funding	Source of funding for pilot trial	456

Citation: Eldridge SM, Chan CL, Campbell MJ, Bond CM, Hopewell S, Thabane L, et al. CONSORT 2010 statement: extension to randomised pilot and feasibility trials. BMJ. 2016;355.

For peer review only

Appendix 1: CONSORT 2010 checklist of information to include when reporting a pilot or feasibility trial

Section/Topic	Item No	Checklist item	Reported on page No
**Title and abstract**			
	1a	Identification as a pilot or feasibility randomised trial in the title	1
	1b	Structured summary of pilot trial design, methods, results, and conclusions (for specific guidance see CONSORT abstract extension for pilot trials)	2
**Introduction**			
Background and objectives	2a	Scientific background and explanation of rationale for future definitive trial, and reasons for randomised pilot trial	3
	2b	Specific objectives or research questions for pilot trial	5, Table 1
**Methods**			
Trial design	3a	Description of pilot trial design (such as parallel, factorial) including allocation ratio	3
	3b	Important changes to methods after pilot trial commencement (such as eligibility criteria), with reasons	N/A
Participants	4a	Eligibility criteria for participants	4
	4b	Settings and locations where the data were collected	3, 4
	4c	How participants were identified and consented	4
Interventions	5	The interventions for each group with sufficient details to allow replication, including how and when they were actually administered	4
Outcomes	6a	Completely defined prespecified assessments or measurements to address each pilot trial objective specified in 2b, including how and when they were assessed	5
	6b	Any changes to pilot trial assessments or measurements after the pilot trial commenced, with reasons	N/A
	6c	If applicable, prespecified criteria used to judge whether, or how, to proceed with future definitive trial	5, 6, Table 1
Sample size	7a	Rationale for numbers in the pilot trial	6
	7b	When applicable, explanation of any interim analyses and stopping guidelines	N/A

For peer review only

Randomisation:			
Sequence generation	8a	Method used to generate the random allocation sequence	4
	8b	Type of randomisation(s); details of any restriction (such as blocking and block size)	4
Allocation concealment mechanism	9	Mechanism used to implement the random allocation sequence (such as sequentially numbered containers), describing any steps taken to conceal the sequence until interventions were assigned	4
Implementation	10	Who generated the random allocation sequence, who enrolled participants, and who assigned participants to interventions	4
Blinding	11a	If done, who was blinded after assignment to interventions (for example, participants, care providers, those assessing outcomes) and how	4
	11b	If relevant, description of the similarity of interventions	N/A
Statistical methods	12	Methods used to address each pilot trial objective whether qualitative or quantitative	6
**Results**			
Participant flow (a diagram is strongly recommended)	13a	For each group, the numbers of participants who were approached and/or assessed for eligibility, randomly assigned, received intended treatment, and were assessed for each objective	6, 7
	13b	For each group, losses and exclusions after randomisation, together with reasons	7, 10, Figure 1
Recruitment	14a	Dates defining the periods of recruitment and follow-up	
	14b	Why the pilot trial ended or was stopped	N/A
Baseline data	15	A table showing baseline demographic and clinical characteristics for each group	Table 2
Numbers analysed	16	For each objective, number of participants (denominator) included in each analysis. If relevant, these numbers should be by randomised group	6-10, Table 4
Outcomes and estimation	17	For each objective, results including expressions of uncertainty (such as 95% confidence interval) for any estimates. If relevant, these results should be by randomised group	10, Table 3, 4

For peer review only

Ancillary analyses	18	Results of any other analyses performed that could be used to inform the future definitive trial	
Harms	19	All important harms or unintended effects in each group (for specific guidance see CONSORT for harms)	8
	19a	If relevant, other important unintended consequences	N/A
**Discussion**			
Limitations	20	Pilot trial limitations, addressing sources of potential bias and remaining uncertainty about feasibility	10
Generalisability	21	Generalisability (applicability) of pilot trial methods and findings to future definitive trial and other studies	10-12
Interpretation	22	Interpretation consistent with pilot trial objectives and findings, balancing potential benefits and harms, and considering other relevant evidence	10-12
	22a	Implications for progression from pilot to future definitive trial, including any proposed amendments	10-12
**Other information**			
Registration	23	Registration number for pilot trial and name of trial registry	3
Protocol	24	Where the pilot trial protocol can be accessed, if available	N/A
Funding	25	Sources of funding and other support (such as supply of drugs), role of funders	13
	26	Ethical approval or approval by research review committee, confirmed with reference number	13

Citation: Eldridge SM, Chan CL, Campbell MJ, Bond CM, Hopewell S, Thabane L, et al. CONSORT 2010 statement: extension to randomised pilot and feasibility trials. BMJ. 2016;355.

For peer review only

Appendix 2: Participant outcomes and health economic data

Comparison of SCI between patients eligible at screening and randomized trial participants

Characteristic	In trial, N = 57[1]	Not in trial, N = 201[1]	p-value[2]
**SCI_Score**	13 (8, 17)	17 (10, 26)	0.004
Unknown	0	49	
[1]Median (IQR)			
[2]Wilcoxon rank sum test			

Pain outcomes at 3 months postoperative (Oxford Knee Score)

Characteristic	Intervention, N = 6[1]	95% CI[2]	Control, N = 9[1]	95% CI[2]
**On-going pain (OKS<14)**				
No on-going pain	5 (83%)	36%, 99%	8 (100%)	60%, 100%
On-going pain	1 (17%)	0.88%, 64%	0 (0%)	0.00%, 40%
Unknown	0		1	
[1]n (%)				
[2]CI = Confidence Interval				

Neuropathic pain outcomes at 3 months postoperative

	**Baseline**		**Pre-operative**		**Post-operative**	
**Characteristic**	Intervention, N = 28[1]	Control, N = 29[1]	Intervention, N = 15[1]	Control, N = 25[1]	Intervention, N = 6[1]	Control, N = 9[1]
**PainDETECT score**	18 (11, 21)	13 (9, 19)	11 (10, 17)	14 (10, 24)	7 (6, 12)	10 (6, 16)
	4	4	6	5	2	3
**PainDETECT category**						
Ambiguous	6 (25%)	5 (20%)	3 (33%)	5 (25%)	0 (0%)	1 (17%)
Neuropathic likely	9 (38%)	8 (32%)	1 (11%)	7 (35%)	1 (25%)	1 (17%)
Nociceptive	9 (38%)	12 (48%)	5 (56%)	8 (40%)	3 (75%)	4 (67%)
Unknown	4	4	6	5	2	3
[1]Median (IQR); n (%)						

EQ-5D-5L and ICECAP scores

Measure	Baseline			Pre-operation			Post-operation		
	Intervention (SD)	Usual care (SD)	Response	Intervention (SD)	Usual care (SD)	Response	Intervention (SD)	Usual care (SD)	Response
EQ-5D-5L	**0.34** (0.30)	**0.47** (0.21)	57/57	**0.35** (0.22)	**0.40** (0.23)	40/57	**0.63** (0.24)	**0.69** (0.13)	14/27*
ICECAP	**0.71** (0.26)	**0.78** (0.14)	56/57	**0.71** (0.23)	**0.80** (0.14)	40/57	**0.83** (0.25)	**0.91** (0.021)	15/27*

*Only collected for those who had TKR within trial.

Intervention costs

**Treatment Cost per person**	**Mean**	**Max**	**Min**
Staff	£103.73	£214.50	£33.00
NHS Treatment cost[§]	30.72	£61.45	£12.29
Societal treatment cost[¥]	£6.59	£19.78	£0
NHS Total (per person)	£134.45	£275.95	£45.29
Societal Total (per person)	£141.04	£295.73	£45.29

[§] Mean - 50% discount given to NHS, Max -no discount, Min - 80% discount offered to NHS.
[¥] Mean -1/3 of participants paid for Headspace and Calm app subscriptions, Max all pay for subscriptions, Min- no one pays for subscription.
*https://www.nice.org.uk/process/pmg36/chapter/economic-evaluation

For peer review only

Appendix 3: Demographics of interviewed participants

Pseudonym	Gender	Age at interview	Ethnicity	Marital status	Intervention or Usual Care	Mode of intervention delivery	Chosen sleep intervention
Florence	Woman	90	Welsh	Widowed	Intervention	Telephone	Relaxation
George	Man	64	White British	Married/Partner	Usual	N/A	N/A
Joyce	Woman	73	White British	Divorced	Usual	N/A	N/A
Gail	Woman	64	White British	Single	Usual	N/A	N/A
Charles	Man	79	English	Widowed	Intervention	Video	CBT-I
Patricia	Woman	69	White English	Single	Intervention	Video	CBT-I
Gloria	Woman	73	White British	Married/Partner	Intervention	Video	Mindfulness
Ruth	Woman	72	English	Married/Partner	Intervention	Video	CBT-I
Arthur	Man	72	White British	Married/Partner	Usual	N/A	N/A
Edward	Man	69	Welsh/British White	Married/Partner	Intervention	Telephone	Mindfulness
Jerry	Man	64	English	Married/Partner	Intervention	Video	Relaxation
Steven	Man	58	White	Married/Partner	Usual	N/A	N/A
Rose	Woman	68	White British	Married/Partner	Intervention	Video	Mindfulness then CBT-I

For peer review only

Appendix 4: Qualitative themes and quotations

Theme	Subtheme	Quotations
**3. Explore engagement with the REST intervention and adherence to the agreed sleep plan**	Practitioner manner and communication style	*Well I think I was speaking to a professional and when I feel that, that gives me a little confidence in talking to her […] and I found that with [the practitioner] she was really very patient and in discussing her ideas […] she was very encouraging actually …[Florence]*<br>*It's been good. Just talk about things and get it into the open, don't bottle everything up [...] it suits me fine. [Jerry]*<br><br>*Well she was quite firm I felt in telling me about relaxing and how I would relax and she really wanted me to make up my mind then and there and tell her what I was planning to do and that made me feel more positive about it [...] she made me discuss it and having done that I was able to make my own mind up… she was suggesting breathing exercises as well […] there was no insistence at all from her, but on the other hand she was very firm in her suggestions [yes] which made me feel that I must do those things [Florence]*
**1. Evaluate intervention acceptability to patients and health professionals**	Patient acceptability: delivery mode	*I'm using Zoom with family etcetera, so I've used it for over a year now and I'm happy with it […] I'm confident using it [Patricia]*<br><br>*I'm not good on this Wi-Fi stuff, I'm really quite awful on it and my poor son was trying to train me [...] I don't know how to cope or how to use anything with Wi-Fi, I find it very irksome, I really do, very overwhelmingly worrying, I just worry about it. [Florence]*<br><br>*I think this sort of appointment you don't need to be face-to-face. […] There's no point doing face-to-face. Yes, it would be a 50-mile round trip for me to come to you […] It's fine seeing each other like this to be honest. [...] Travel time, parking, petrol. It's saving a fair bit of money from my point of view. [Patricia]*<br><br>*I don't see a disadvantage. Sitting in my own home rather than going to a hospital where I could pick up more diseases or COVID again. [Gloria]*
	Patient acceptability: appointment length and structure	*There was somebody there who could give me suggestions on the way whilst waiting for my operation, do you know what I mean? It's almost as though she was giving me something to do and think about as I approached the operation … [Florence]*

For peer review only

		The phone call took place not at the right time. But that was fine by me because I wasn't doing anything else. [Patricia]<br><br>*I'm not pleased, I've taken time off work to do this, but I was happy to take time off work to do it but not to be able to do it… [Ruth]*
	Practitioner acceptability: mode of delivery	*I'd be the first to admit that my IT is not as good as it should. I don't do much in the way of IT. […] I'm not the slickest at getting it all up on the computer. […] I find the IT thing a bit of a challenge sometimes [ESP03].*<br><br>*They've all been there ready and waiting and a few times, I've been late finishing the morning clinic because I normally do them on a Monday afternoon. They've all been ready and waiting at their phone or their computer. [ESP03]*
	Practitioner acceptability: intervention handbook and paperwork	*The form was very easy to follow for all the questions [ESP01]*<br><br>*The actual booklet that you're given yes, that's easy. I think maybe I probably need to introduce it a little bit better and what the study is, but then the actual questions are easy to follow, and then it follows onto the SMART goals and things, yes. [ESP01]*<br><br>*I think overall fine, I think sometimes you do feel like you're duplicating quite a lot I think, and when I was actually working through paperwork too quickly and not really sitting down and really going in depth about what the question's asking. Sometimes it feels like, well see above, kind of thing. Maybe that's because I'm classically the kind of person that will write everything down in the first box [RE 4-week call paperwork] [ESP04]*
**6. Evaluate the acceptability of randomisation**		*I understand that that's the only way you can gauge whether what you're doing is of any benefit if it's all randomised. You're not picking out a group of people that are better than another group of people in terms of their symptoms. [Joyce]*<br><br>*I would have particularly liked it if I was one of the people that was offered sort of help with sleep and so on, because the ideal for me would be to find an alternative to running through the highs and lows of my life at three o'clock in the morning would be good. So I was a bit disappointed. [Arthur]*
**9. Collect data on patient-reported outcomes measures to assess data**		*Things affect you differently at different times […] some days, if you're feeling really well, I think you fill it in through rose-tinted glasses. That's the only way I can describe it. If you were having a really good day*

For peer review only

**completion rates and inform the selection of the primary outcome measure and sample size for a full trial**		*and things were going well, you'd fill it in a little bit differently … [Joyce]*

For peer review only

BMJ Open

REST: A pre-operative tailored sleep intervention for patients undergoing total knee replacement: feasibility study for a randomised controlled trial

Journal:	*BMJ Open*
Manuscript ID	bmjopen-2023-078785.R1
Article Type:	Original research
Date Submitted by the Author:	12-Dec-2023
Complete List of Authors:	Bertram, Wendy; University of Bristol Medical School, Musculoskeletal Research Unit; NIHR Bristol Biomedical Research Centre<br>Penfold, Chris; University of Bristol Medical School, Musculoskeletal Research Unit<br>Glynn, Joel; University of Bristol Medical School<br>Johnson, Emma; University of Bristol Medical School<br>Burston, Amanda; University of Bristol Medical School, Musculoskeletal Research Unit<br>Rayment, Dane; North Bristol NHS Trust<br>Howells, Nicholas; North Bristol NHS Trust<br>White, Simon; Cardiff and Vale University Health Board<br>Wylde, Vikki; University of Bristol Medical School, Musculoskeletal Research Unit; NIHR Bristol Biomedical Research Centre<br>Gooberman-Hill, Rachael; University of Bristol Medical School; NIHR Bristol Biomedical Research Centre<br>Blom, Ashley; The University of Sheffield<br>Whale, Katie; University of Bristol Medical School, Musculoskeletal Research Unit; NIHR Bristol Biomedical Research Centre
**Primary Subject Heading**:	Health services research
Secondary Subject Heading:	Qualitative research, Surgery
Keywords:	Knee < ORTHOPAEDIC & TRAUMA SURGERY, SLEEP MEDICINE, Feasibility Studies

SCHOLARONE™
Manuscripts

I, the Submitting Author has the right to grant and does grant on behalf of all authors of the Work (as defined in the below author licence), an exclusive licence and/or a non-exclusive licence for contributions from authors who are: i) UK Crown employees; ii) where BMJ has agreed a CC-BY licence shall apply, and/or iii) in accordance with the terms applicable for US Federal Government officers or employees acting as part of their official duties; on a worldwide, perpetual, irrevocable, royalty-free basis to BMJ Publishing Group Ltd ("BMJ") its licensees and where the relevant Journal is co-owned by BMJ to the co-owners of the Journal, to publish the Work in this journal and any other BMJ products and to exploit all rights, as set out in our licence.

The Submitting Author accepts and understands that any supply made under these terms is made by BMJ to the Submitting Author unless you are acting as an employee on behalf of your employer or a postgraduate student of an affiliated institution which is paying any applicable article publishing charge ("APC") for Open Access articles. Where the Submitting Author wishes to make the Work available on an Open Access basis (and intends to pay the relevant APC), the terms of reuse of such Open Access shall be governed by a Creative Commons licence – details of these licences and which Creative Commons licence will apply to this Work are set out in our licence referred to above.

Other than as permitted in any relevant BMJ Author's Self Archiving Policies, I confirm this Work has not been accepted for publication elsewhere, is not being considered for publication elsewhere and does not duplicate material already published. I confirm all authors consent to publication of this Work and authorise the granting of this licence.

For peer review only

Title

REST: A pre-operative tailored sleep intervention for patients undergoing total knee replacement: feasibility study for a randomised controlled trial

Target Journal: BMJ Open BMJ Open submission guidelines

Authors

Wendy Bertram[1,2], Chris Penfold[1], Joel Glynn[1], Emma Johnson[1], Amanda Burston[1], Dane Rayment[3], Nick Howells[3], Simon White[4], Vikki Wylde[1,2], Rachael Gooberman-Hill[1,2], Ashley Blom[5], Katie Whale[1,2].

Corresponding Author: Wendy Bertram, Wendy.Bertram@bristol.ac.uk

Affiliations

1. University of Bristol
2. NIHR Bristol Biomedical Research Centre, University Hospitals Bristol and Weston NHS Foundation Trust and University of Bristol
3. North Bristol NHS Trust
4. Cardiff and Vale University Health Board
5. University of Sheffield

Abstract

Objectives

To test the feasibility of a randomised controlled trial of a novel pre-operative tailored sleep intervention for patients undergoing total knee replacement.

Design

Feasibility two-arm two-centre randomised controlled trial using 1:1 randomisation with an embedded qualitative study.

Setting

Two NHS secondary care hospitals in England and Wales.

Participants

Pre-operative adult patients identified from total knee replacement waiting lists with disturbed sleep, defined as a score of 0-28 on the Sleep Condition Indicator questionnaire.

Intervention

The REST intervention is a pre-operative tailored sleep assessment and behavioural intervention package delivered by an Extended Scope Practitioner (ESP), with a follow-up phone call four-weeks post-intervention. All participants received usual care as provided by the participating NHS hospitals.

Outcome measures

The primary aim was to assess the feasibility of conducting a full trial. Patient reported outcomes were assessed at baseline, one-week pre-surgery, and 3-months post-surgery. Data collected to determine feasibility included the number of eligible patients, recruitment rates, and intervention adherence. Qualitative work explored the acceptability of the study processes and intervention delivery through interviews with ESPs and patients.

Results

Screening packs were posted to 378 patients and 57 patients were randomised. Of those randomised, 20 had surgery within the study timelines. An appointment was attended by 25/28 (89%) of participants randomised to the

For peer review only - http://bmjopen.bmj.com/site/about/guidelines.xhtml

intervention. Follow-up outcomes measures were completed by 40/57 (70%) of participants pre-surgery and 15/57 (26%) post-surgery. Where outcome measures were completed, data completion rates were 80% or higher for outcomes at all timepoints, apart from the painDETECT: 86% complete at baseline, 72% at pre-surgery and 67% post-surgery. Interviews indicated that most participants found the study processes and intervention acceptable.

Conclusions

This feasibility study has demonstrated that with some amendments to processes and design, an RCT to evaluate the clinical and cost-effectiveness of the REST intervention is feasible.

Trial Registration: ISRCTN14233189

Article Summary: Strengths and limitations of this study

- COVID-19 restrictions in place during delivery required study procedures to be redesigned to enable remote data collection.
- Data collection at the 3-month post-surgery timepoint was limited due to the volume of operations performed during the study.
- This study was undertaken in two National Health Services hospitals, which demonstrates that it is feasible to undertake a full trial in these settings, however the findings may not necessarily be generalisable to other settings.
- Embedded qualitative work provided important insight into final study design to support acceptability and participant engagement with a full trial.

For peer review only

Background

Over 100,000 total knee replacements (TKR) are performed yearly in the UK.[1, 2] The primary reason for surgery is severe chronic pain and functional limitation due to end-stage osteoarthritis. The aim of TKR is long-term pain relief and improved function. Outcomes after knee replacement surgery are good, and surgical complications are rare, however approximately 20% of patients report dissatisfaction due to ongoing pain and functional limitations[3, 4].

Sleep issues are a substantial problem for people awaiting joint replacement; 60-75% of people with osteoarthritis and 70% of patients awaiting joint replacement experience sleep problems, which increase with condition severity.[5-8] Patients report issues with sleep onset and maintenance, and middle of the night waking.[9]

Poor sleep causes worsening joint pain, depressive symptoms, lower physical activity, and increased risk of cardiovascular and pulmonary disease in patients with osteoarthritis.[10, 11] Poor sleep can negatively impact surgical recovery causing slower wound healing, impaired immune function, increased risk of infection, and longer hospital stays.[12-15] Surgical patients with pre-operative sleep disturbances are at greater risk of developing post-operative delirium and surgical complications.[16] Sleep is bidirectionally linked with pain with poor sleep increasing pain sensitivity and inflammatory markers associated with pain[15, 17]. Poor sleep before TKR is associated with increased acute and chronic post-surgical pain, increased analgesic use, reduced joint function and range of motion, lower satisfaction, and longer inpatient stays.[8, 18, 19]

Previous trials on sleep and joint replacement have predominantly focused on peri- and post-operative pharmacological interventions.[20-22] A recent systematic review identified that improved pre-operative sleep reduced pain levels and analgesic consumption after TKR.[23] Guidance from the National Institute for Health and Care Excellence (NICE) and European Alliance of Associations for Rheumatology (EULAR) advises avoidance of pharmacological therapy for long-term management of sleep issues and recommends behavioural approaches as first-line treatment.[24, 25] Non-pharmacological sleep interventions are potentially more sustainable and cost-effective, with lower risk of side-effects.[26] Our recent systematic review identified no RCTs evaluating a non-pharmacological intervention targeting sleep in patients waiting for TKR.[27]

This study aimed to evaluate the feasibility of conducting an RCT to evaluate the clinical and cost-effectiveness of REST, a non-pharmacological complex sleep intervention for patients undergoing TKR.

Methods

Study design

REST is a two-centred randomised controlled feasibility trial with 1:1 randomisation and an embedded qualitative study. Participants were recruited from two secondary care NHS hospitals in England and Wales. Ethics approval was obtained from the South West Cornwall & Plymouth Research Ethics Committee (20/SW/0189). The trial was prospectively registered (ISRCTN14233189). The CONSORT checklist is provided in appendix 1.

Patient and public involvement (PPI)

For peer review only - http://bmjopen.bmj.com/site/about/guidelines.xhtml

The study was developed in collaboration with a musculoskeletal PPI group working in partnership with the University of Bristol and North Bristol NHS Trust. The study benefited from the active involvement and contributions from a group of experienced patient partners. The group comprised five patients with lived experience of knee replacement. They met four times to co-design patient-facing study materials, monitor study progress, provide input into study process, and review the study results and dissemination plans. A patient partner was a member of the Steering committee.

Participant identification, recruitment and randomisation

Patients waiting for a primary TKR for osteoarthritis were identified from surgical waiting lists. Those likely to have surgery within three months were sent a pre-notification card by post followed by a screening questionnaire. The three-month preoperative timepoint was selected to allow sufficient time for intervention delivery and engagement to affect behaviour change.

Eligibility criteria were: adults on the TKR waiting list, experiencing disturbed sleep (defined as a score of 0-28 on the Sleep Condition Indicator (SCI) questionnaire, a validated screening tool for insomnia[28]), and access to a device with internet connection. Exclusion criteria were: diagnosed with or receiving treatment for a clinical sleep disorder, taking prescription medication to help with sleep, having taken part in an interventional sleep study in the past six months, and unable or unwilling to attend an intervention appointment, provide informed consent or complete questionnaires in English. Patients who returned an eligible screening questionnaire were telephoned for an eligibility assessment. Eligible patients were invited to a recruitment visit. Patients not eligible at screening were sent a thank you letter.

After written informed consent was provided and the baseline questionnaire completed, participants were randomly allocated to the intervention plus usual care or usual care alone. Randomisation was conducted on a 1:1 intervention:control basis by the co-ordinating centre using computer generated randomisation. Participants, practitioners, and research staff were not blinded.

Intervention

The REST intervention was developed following Medical Research Council guidance for complex intervention development.[29, 30] The TIDieR checklist is provided in the appendix 2. REST consists of an appointment with an Extended Scope Practitioner (ESP) delivered via videoconference or telephone three months before surgery. The one-hour appointment comprised a comprehensive sleep assessment to identify individual sleep issues and needs, and an assessment of sleep apnoea risk. Participants scoring high risk for sleep apnoea were referred to their GP in addition to the intervention. Participants were then provided with tailored sleep education and sleep hygiene advice. One of three existing evidence-based sleep interventions (ESIs) was recommended through a shared decision-making process: cognitive-behavioural therapy for insomnia (delivered via online platform Sleepstation), relaxation (delivered via the Calm app, workbook, or guided audio/video), and mindfulness (delivered via the Headspace app, workbook, or guided audio/video).

Participants were provided with a personalised sleep plan, which included SMART goals based on the sleep hygiene recommendations (for example reducing coffee intake, removing electronics from the bedroom, starting a bedtime routine / sleep schedule), a detailed overview of their chosen ESI, instructions for use and digital access (if applicable) and any materials. Participants received a follow-up telephone call four weeks after the appointment to

For peer review only - http://bmjopen.bmj.com/site/about/guidelines.xhtml

review progress and engagement with their sleep plan, calls lasted approximately 30-45 minutes. This included addressing any barriers experienced, review of the sleep goals, and adjustments to the sleep plan if needed.

All participants received usual care as provided by the participating NHS hospitals. Safety reporting was exclusively for adverse reactions directly attributable to the intervention.

Intervention delivery training

All practitioners took part in a one-day online intervention delivery training session. This covered the study background, evidence on the relationship between sleep and pain, an overview of each ESI, and practical guidance on delivery. The Chief Investigator communicated regularly with practitioners to provide further support and training if required. ESPs were provided with a detailed intervention manual, which provided guidance and proformas for conducting the sleep assessment, sleep hygiene and education advice, information on each ESI, participant sleep plan, and post-appointment tasks.

Intervention timing

REST was designed to be delivered 3-months pre-surgery. This timepoint was chosen to optimise the effect of the sleep interventions, because of the duration of the sleep interventions (Sleepstation is delivered over 6-8 weeks), and theories of behaviour change maintenance[31].

Intervention delivery fidelity

Non-participatory observations were conducted to assess the degree to which the intervention was delivered as intended as per the intervention manual. One clinic appointment and follow-up call were observed for each ESP. Observations were conducted independently by two members of the research team. Participants were asked to provide verbal consent for the researcher to be present during their clinic appointment.

Feasibility outcomes

Feasibility outcomes included recruitment rate, intervention uptake and adherence, outcome data completion, and intervention acceptability[32]. A full list of outcomes and measurements are outlined in Table 1.

Table 1: Feasibility outcomes

Objective(s)	Outcome	Measurement
1	Eligibility and recruitment rates	Number of patients invited, returning screening questionnaires, eligible, consented and randomised. Retention rates.
2	Intervention uptake	Number of participants who attend the clinic appointment
3	Intervention adherence	Open ended questions at 4-week follow-up telephone call:<br>• Changes made as a result of sleep hygiene and education advice<br>• Engagement in the assigned sleep intervention<br>• Any additional changes made to sleep or sleep routine
4 & 6	Participant interviews: acceptability of the	Semi-structured qualitative interviews with participants in the intervention group (n=20, 10 per site), and the control group (n=5).

For peer review only - http://bmjopen.bmj.com/site/about/guidelines.xhtml

	intervention and randomisation	
4 & 8	Extended scope practitioner interviews: acceptability of the intervention and training optimisation	Semi-structured qualitative interviews with ESPs (n=4) at two timepoints: 1) after completion of training and 2) after intervention delivery
5	Intervention delivery fidelity	Observation of one clinic appointment and follow-up call for each ESP to assess adherence and compliance.
7	Health economics data	Quality of life measures (EQ-5D-5L, ICECAP-A) and health care resource use (community and secondary care) as documented in the patient completed outcome measure booklets at one week pre-operative and three months post-operative.
8	Optimisation of intervention training	Non-participatory observations of the ESP training.<br>Semi-structured interviews with all ESPs (n=4) at two time points, after completion of training and after delivering the intervention.
9	Inform the primary outcome measure for a full trial	Quantitative data analysis, proportion of participants in ongoing pain in each treatment arm at three months after surgery.

For peer review only - http://bmjopen.bmj.com/site/about/guidelines.xhtml

Patient-reported outcomes

Patient-reported outcomes were assessed using paper questionnaires prior to randomisation (approximately 3 months pre-operative), one week prior to surgery and three months after surgery. Participants who did not have their operation by six-months post-randomisation completed pre-surgery outcomes.

Outcomes included joint pain (Oxford Knee Score (OKS)[33]), neuropathic pain (painDETECT[34]), sleep quality and beliefs about sleep (Sleep Condition Indicator (SCI[28]), Pittsburgh Sleep Quality Index (PSQI)[35]), mental wellbeing (Hospital Anxiety and Depression Scale (HADS)[36]), and general health and wellbeing (EQ-5D-5L[37],ICECAP-A[38]). Health resource use data included health care interactions in the community and secondary care, including medication use, and were collected in the three-month pre-operative and post-operative questionnaires only. Intervention participants completed sleep treatment engagement questions at the 3-month post-surgery timepoint.

Qualitative study

Embedded qualitative work explored the acceptability of the intervention and study processes. Interviews were conducted with participants in both arms and with ESPs delivering the intervention. Participants who expressed an interest at enrolment in being interviewed were sent an invitation letter, reply slip, and pre-paid envelope. Participants who returned the reply slip were contacted a researcher to discuss participation and arrange an interview for those interested. Informed consent was provided by participants before interview. ESPs were invited to participate in two interviews: one following the intervention training day and one after delivery of intervention appointments. Informed written or recorded verbal consent was provided by all ESPs.

Qualitative data collection

All interviews were conducted via videoconference or telephone depending on preference. Face-to-face interviews were not possible due to COVID-19 restrictions. Participant interviews were guided by semi-structured topic guides (Appendix 3) covering design and conduct of the trial (all participants), experiences of the intervention and views on impact (intervention group), and changes to made to sleep (all participants). ESP interviews at both timepoints explored acceptability of training and intervention delivery.

Progression criteria

Progression criteria for demonstrating the feasibility of a RCT were proposed as ≥60 patients randomised (75% of target) and 75% uptake of the intervention. Uptake was defined as the number of participants who attended an intervention appointment. Criteria for progression based on acceptability were:

- All participants: expressed comfort with study processes including recruitment, randomisation, outcome measures and follow-up.
- Intervention participants: level of engagement with clinic processes, adherence to and engagement with the intervention.

Sample size

The target sample was 80 participants (40 intervention, 40 usual care) to estimate 75% randomisation rate (RCT progression criteria) with 95% confidence interval from 65% to 85%, and to estimate 75% intervention uptake with 95% confidence interval from 60% to 90%.

Statistical analyses

For peer review only - http://bmjopen.bmj.com/site/about/guidelines.xhtml

Baseline characteristics of each group were tabulated using means and standard deviations for normally distributed data, medians and interquartile ranges for non-normally distributed data, and percentages and counts for categorical data. Patient-reported outcome measures were summarised descriptively. The proportion of people without complete responses for the outcome questionnaires were reported at each timepoint with commentary on any patterns of missing data between timepoints. Outcome data tables are provided in appendix 4.

Economic analyses

The feasibility of collecting data for an economic evaluation alongside a full trial was assessed, including intervention costs for appointments and tailored intervention. Economic analysis tables are provided in appendix 4.

Qualitative analysis

Data collection and analysis were conducted in parallel after the first three interviews. Audio files were transcribed, then transcripts were anonymised and imported into the qualitative software package NVivo10. Participant and ESP data were analysed separately using Framework Analysis, a thematic approach that enables structured comparison and contrast of data across cases.[39] Data were organised using the topic guide as a starting framework. Five transcripts were independently double coded and discussed within the team to offer further insight into interpretation and to enhance rigour through different approaches and knowledge.[40] All participants were assigned pseudonyms to ensure anonymity. Participant demographics and supporting quotes are included in appendices 5 and 6.

Results

1. Eligibility and recruitment rates

Between March and December 2021, 378 patients were invited to take part in screening. Of these, 258 (68%) returned completed screening questionnaires: 146 were willing to take part and met screening eligibility criteria, 58 declined to take part and 54 were not eligible. Reasons for ineligibility included: SCI score ≥29 (28/54, 52%), having a sleep disorder or taking medication to help with sleep (18/54, 33%), operation dates allocated in the near future or surgery being postponed (4/54, 7%), questionnaire returned after study closure (3/54, 6%), and no internet access (1/54, 2%).

Telephone calls were made to the 146 patients who returned an eligible screening questionnaire: 8 were not contactable, 32 were ineligible, 47 declined to take part and 59 consented to take part. Reasons for ineligibility at telephone screening included operation dates allocated in the near future or surgery being postponed (13/32, 41%), having a sleep disorder or taking medication to help with sleep (9/32, 28%), no internet access (7/32, 22%), already had surgery (2/32, 6%), and did not speak English (1/32, 3%). Most did not give a reason for declining taking part. Where given, common reasons for declining were time commitments or personal circumstances (17/47, 36%) and did not feel they had a sleep problem or did not think treatment would help (6/47, 13%).

Two participants withdrew prior to randomisation; therefore 57 participants were randomised.

A CONSORT diagram outlining participant flow is provided in Figure 1. Baseline participant characteristics are provided in Table 2. Patients who were eligible but did not participate had higher (better) preoperative sleep as measured using the SCI than randomised participants (appendices).

For peer review only - http://bmjopen.bmj.com/site/about/guidelines.xhtml

Table 2: Characteristics of study participants at baseline

Characteristic	N = 57[1]
**Gender**	
Man	23 (40%)
Woman	34 (60%)
**Ethnicity (cleaned)**	
Non white/non British	1 (1.8%)
Other/not answered	4 (7.0%)
White/British	52 (91%)
**Do you consume any alcohol?**	
No	26 (46%)
Yes	30 (54%)
Unknown	1
**Alcohol units/week (excl. non-drinkers)**	9 (3, 14)
Non-drinker/unknown	27
**Do you consume any coffee?**	
No	16 (28%)
Yes	41 (72%)
**Cups of coffee/week (excl. non-drinkers)**	12 (7, 20)
Non-drinker/unknown	16
**Smoking status**	
Current	2 (3.5%)
Former	24 (42%)
Never	31 (54%)
**Employment status**	
Employed	11 (19%)
Other	2 (3.5%)
Retired	44 (77%)
**Marital Status**	
Divorced	9 (16%)

For peer review only - http://bmjopen.bmj.com/site/about/guidelines.xhtml

Characteristic	N = 57[1]
Married/partner	36 (63%)
Single	2 (3.5%)
Widowed	10 (18%)
**Other conditions**	
No	18 (34%)
Yes (please state)	35 (66%)
Unknown	4
**Other condition(s): Other joint replacement**	
Yes	4 (100%)
Unknown	53
**Other condition(s): Pain in other joints**	
Yes	4 (100%)
Unknown	53
**Other condition(s): Arthritis (any)**	
Yes	29 (100%)
Unknown	28
**Other condition(s): Injury**	
Yes	3 (100%)
Unknown	54
[1]n (%)	

2. Attendance at the intervention clinic appointment

Of the 28 participants assigned to the intervention group, 3 withdrew before receiving the intervention due to operation dates being allocated in the near future (10.7%), 25 attended the clinic appointment (89.3%), and 15 completed the 4-week follow-up call (53.6%).

3. Engagement with the REST intervention and adherence to the agreed sleep plan

Qualitative study: participant interviews

38 trial participants were invited to take part in an interview (N=18 Bristol/20 Cardiff; 22 intervention, 16 usual care). Sixteen expressions of interest were received, and 13 participants were interviewed (N= 10 Bristol/3 Cardiff; N= 8 intervention/5 usual care. Ethnicity was reported as White British (n=7), English (3), Welsh (n=2), and White (n=1).

For peer review only - http://bmjopen.bmj.com/site/about/guidelines.xhtml

Marital status was reported as married/partner (n=8), widowed (n=2), single (n=2), and divorced (n=1). Further participant characteristics are described in Appendix 5.

Participants were willing to discuss their sleep issues with the ESPs. Factors influencing readiness to engage in a conversation with the practitioner included a belief that they were meeting with a knowledgeable and skilled professional. The practitioner's manner and communication style helped to create a safe space that enabled participants to feel both at ease and comfortable to open up about their experiences. Participants who recalled the shared decision-making process of choosing a sleep intervention said that they felt involved and informed.

4. Intervention acceptability

Patient acceptability

COVID-19 restrictions at the time of delivery required all recruitment and intervention appointments to be conducted remotely by videoconference or telephone. Participants understood the need for remote appointments, but confidence and familiarity with this approach varied. For many participants confidence in using video calls had grown during the pandemic. One participant struggled with confidence and familiarity with online appointments and needed additional support. Remote delivery was generally seen as acceptable. Participants highlighted benefits of removed travel and cost, and reduced risk of COVID-19 and other infections.

Appointment length and structure were considered appropriate. Participants felt they had enough time to ask questions, discuss what was being asked of them, and to address concerns. The time to attend the appointment was seen as worthwhile, as it gave access a practitioner who provided the chance to talk about their problems and a focus during their wait for surgery.

Due to clinic delays and competing demands of the practitioners, some appointments started later than planned. Although some participants were accepting of this, one participant reported feeling frustrated and angry at the inconvenience caused.

No adverse events were reported.

Practitioner acceptability

ESP acceptability of intervention delivery was high. ESPs were able to deliver most appointments using videoconference which supported better communication with participants, however one ESP expressed a preference for telephone appointments as this required less set up and had greater flexibility. Some technical issues were raised due to ESPs using different NHS computers for appointments depending on their schedule. This caused problems with webcam connectivity and added additional time to appointment set up. Overall paperwork was straightforward to complete with the questions and proformas clear and easy to use. Some aspects of the assessment were viewed as repetitive and could be shortened to give more time for discussion with participants.

5. Intervention delivery

Intervention fidelity assessments included observation of at least one clinic appointment and one follow-up appointment for each practitioner. Practitioners fully or partially met all areas of adherence (fidelity to the intervention as described in the manual) and compliance (proficiency of delivery) during the intervention

For peer review only - http://bmjopen.bmj.com/site/about/guidelines.xhtml

appointment. Three areas for improvement in training and delivery were identified: educating the participant about sleep and TKR, setting SMART sleep goals, and shared-decision-making discussions around recommended sleep interventions.

6. Acceptability of randomisation

Qualitative interviews with participants found that most participants found randomisation acceptable and understood the need for this design. Some expressed disappointment on receiving usual care. Disappointment stemmed from their desire to benefit from the intervention because of struggles with sleep.

7. Feasibility and acceptability of collecting health economic data

Quality of life measures

EQ-5D-5L and ICECAP mean scores and standard deviations with response rates at each of the time point are presented in the appendices. EQ-5D-5L utility scores were calculated using Hernández Alava et al. 2020 method as recommended by NICE PMG36.[41] No evidence was found of ceiling of floor effects for either quality of life measure, and the measures seem to be responsive to quality of life changes in this population.

Resource use

Responses to the bespoke resource use questionnaire were 40/57 (70%) pre-operatively and 14/27 (52%) post-operatively. Changes and clarification of questions will be made based on responses to individual questions. For example, more options are needed for physiotherapy appointments as many responses were selected "other". Most resource use and cost fell on NHS services including GP, outpatient, and physiotherapy appointments. Overall, the estimated cost (NHS perspective, excluding intervention costs) was £507 and £688 for intervention and usual care arms respectively. However, no interpretation of this difference can be made given the small number of participants in this feasibility study.

Intervention costs

Given we do not know the value or mechanism of payments from NHS to Sleepstation for use of the app, nor the number of patients purchasing app subscriptions to Calm and Headspace, we costed the intervention based on several assumptions (appendices). We have generated a minimum, mean, and maximum expected intervention cost. The mean cost of the intervention was estimated at £134.45 per person (£141.04 including patient out of pocket costs) based on the mean clinic, preparation, and post-clinic time captured in the feasibility study, an NHS cost of £147 per patient for the use of Sleepstation (based on a 50% discount on publicly advertised cost), and 1/3 of those using Calm or Headspace upgrading to paid subscriptions for additional access. The max estimated at £295.73 and minimum cost £45.29.

8. Optimisation of the intervention delivery training package

Four ESPs took part in an interview at timepoint one (post-training) and three at time-point two (post-delivery).

Training was delivered as a one-day online course. Practitioners spoke positively about their experience of the training, praising the organisation and focus. Practitioners appreciated receiving information about the rationale behind the intervention. Some felt the level of detail around sleep science could be reduced, but still found this interesting. They were given sufficient time to ask questions, however the remote format made this slightly harder

For peer review only - http://bmjopen.bmj.com/site/about/guidelines.xhtml

compared to face-to-face. Following experience of intervention delivery, ESPs reported challenges in setting SMART goals including what areas to focus on and the level of detail needed. They suggested more training and knowledge of each ESI would be beneficial and facilitate better shared decision-making discussions with participants. A small number of participants attending appointments felt they did not have a sleep issue. This made it challenging for ESPs to follow the intervention handbook.

Areas for improving practitioner training were identified as:

- Increased time for role-play and practical exercises
- Additional information on sleep interventions to increase understanding and familiarity
- Further training and practical exercises on setting SMART goals
- Advice and guidance on how to support participants who do not believe they have a sleep issue or are not motivated to make changes
- Additional supervision meetings throughout intervention delivery to provide further support, answer questions, and address challenges

9. Data completion rates, selection of the primary outcome measure and sample size for a full trial

Data completion rates

Data completion rates are provided in Table 3. All participants completed the baseline OKS and EQ5D-5L. Completion rates were consistently lower for the PSQI (86%-93%) at baseline and pre-operative timepoints. The painDETECT questionnaire had the lowest completion rates at each timepoint, ranging from 60-86%.

Table 3: Data completion rates

	Baseline		Pre-operative		Post-operative	
Characteristic	Intervention, N = 28[1]	Control, N = 29[1]	Intervention, N = 15[1]	Control, N = 25[1]	Intervention, N = 6[1]	Control, N = 9[1]
**OKS**						
Complete	28 (100%)	29 (100%)	13 (87%)	25 (100%)	6 (100%)	8 (89%)
Missing			2 (13%)	0 (0%)	0 (0%)	1 (11%)
**PainDETECT**						
Complete	24 (86%)	25 (86%)	9 (60%)	20 (80%)	4 (67%)	6 (67%)
Missing	4 (14%)	4 (14%)	6 (40%)	5 (20%)	2 (33%)	3 (33%)
**SCI**						
Complete	27 (96%)	27 (93%)	15 (100%)	24 (96%)	6 (100%)	9 (100%)
Missing	1 (3.6%)	2 (6.9%)	0 (0%)	1 (4.0%)		
**HADS**						
Complete	27 (96%)	29 (100%)	12 (80%)	25 (100%)	6 (100%)	9 (100%)

For peer review only - http://bmjopen.bmj.com/site/about/guidelines.xhtml

Missing	1 (3.6%)	0 (0%)	3 (20%)	0 (0%)		
**PSQI**						
Complete	24 (86%)	27 (93%)	13 (87%)	21 (84%)	6 (100%)	9 (100%)
Missing	4 (14%)	2 (6.9%)	2 (13%)	4 (16%)		
**EQ5D-5L**						
Complete	28 (100%)	29 (100%)	15 (100%)	25 (100%)	6 (100%)	8 (89%)
Missing					0 (0%)	1 (11%)
**ICECAP**						
Complete	25 (89%)	29 (100%)	15 (100%)	25 (100%)	6 (100%)	9 (100%)
Missing	3 (11%)	0 (0%)				
[1]n (%)						

Qualitative interviews demonstrated that questionnaire completion was acceptable overall.

Patient-reported outcome measures

Baseline, pre-operative, and post-operative outcome measures are presented Table 4. The purpose of this study was to evaluate the feasibility of conducting an RCT, therefore statistical tests to compare outcomes between treatment arms were not performed.

peer review only

427 Table 4: Patient reported outcome measures

	Baseline		Pre-operative		Post-operative	
	Intervention, N = 28[1]	Usual Care, N = 29[1]	Intervention, N = 15[1]	Usual Care, N = 25[1]	Intervention, N = 6[1]	Usual Care, N = 9[1]
**OKS pain subscale**	8.5 (6.0, 10.0)	9.0 (6.0, 11.0)	7.0 (6.0, 10.0)	8.0 (5.0, 12.0)	13.50 (11.50, 15.50)	14.50 (12.50, 16.00)
**OKS function subscale**	7.5 (3.8, 12.0)	9.0 (7.0, 12.0)	7.0 (5.0, 9.8)	8.0 (5.0, 11.0)	21.5 (19.5, 22.8)	22.5 (21.0, 24.0)
**OKS total score**	16 (12, 21)	18 (13, 22)	15 (11, 19)	16 (9, 22)	34 (32, 40)	36 (35, 39)
**PainDetect score**	18 (11, 21)	13 (9, 19)	11 (10, 17)	14 (10, 24)	7 (6, 12)	10 (6, 16)
**PainDetect score(categorised)**						
Ambiguous	6 (25%)	5 (20%)	3 (33%)	5 (25%)	0 (0%)	1 (17%)
Neuropathic likely	9 (38%)	8 (32%)	1 (11%)	7 (35%)	1 (25%)	1 (17%)
Nociceptive	9 (38%)	12 (48%)	5 (56%)	8 (40%)	3 (75%)	4 (67%)
**Sleep conditions indicator**	11 (8, 14)	13 (10, 16)	14.0 (11.0, 21.0)	15.0 (9.5, 18.0)	22 (16, 24)	24 (18, 27)
**HADS score**	13 (10, 22)	16 (12, 19)	12.5 (9.0, 19.0)	14.0 (9.0, 20.0)	9.0 (4.2, 13.0)	8.0 (6.0, 14.0)
**HADS score (categorised)**						
Abnormal	19 (70%)	22 (76%)	7 (58%)	17 (68%)	3 (50%)	3 (33%)
Borderline abnormal	5 (19%)	5 (17%)	3 (25%)	6 (24%)	0 (0%)	2 (22%)
Normal	3 (11%)	2 (6.9%)	2 (17%)	2 (8.0%)	3 (50%)	4 (44%)
**ICECAP**	0.85 (0.66, 0.92)	0.84 (0.70, 0.89)	0.84 (0.55, 0.90)	0.84 (0.67, 0.91)	0.92 (0.87, 0.98)	0.91 (0.89, 0.92)
**EQ-5D**	0.33 (0.16, 0.57)	0.54 (0.30, 0.70)	0.39 (0.22, 0.54)	0.45 (0.22, 0.60)	0.67 (0.60, 0.73)	0.76 (0.73, 0.77)
**PSQI global score**	12.0 (8.8, 14.2)	11.0 (8.0, 13.5)	8.0 (6.0, 11.0)	11.0 (7.0, 13.0)	9.5 (5.2, 11.5)	7.0 (5.0, 9.0)

[1]Median (IQR); n (%)

Participants randomised to the intervention group reported an improvement in average Pittsburgh Sleep Quality Index score from 12.0 (95% CI 8.8, 14.2) at baseline to 8.0 (95% CI 6, 11) at the end of the intervention (12 weeks after randomisation), compared to no change in the usual care group (baseline score of 11 (95% CI 8, 13.5) and 12-week score of 11 (95% CI 7, 13)).

A proposed primary outcome was pain after surgery as measured by the OKS pain component[42]. The target timing for randomisation was three months pre-operative. The mean number of days from randomisation to operation was 118 days, with 35% (n=20) of participants having surgery during the six-month participation window. Outside of the participation window, a further 18 participants were allocated an operation date. The remaining 21 had not been allocated a surgery date at study closure.

Discussion

This feasibility study has demonstrated that the REST intervention is acceptable to patients and clinicians. With modifications, a full trial is feasible. Criteria for progression to a full trial are ≥60 patients randomised (75% of target) and 75% uptake of the intervention. More than 75% of participants allocated the intervention attended the clinic appointment (89%, n = 25/29). We randomised 57 patients during a period of COVID-19 restrictions when many studies were unable to recruit. In addition, removing the need to screen patients who are three months prior to surgery would facilitate increased recruitment.

Strengths

Despite COVID-19 restrictions at the time of the study, screening and recruitment procedures were successful and 57 participants were randomised. Close working with waiting list staff and surgeons was essential to understanding which patients were most likely to be allocated a surgery date in three months. Once identified, the return rate for screening questionnaires was 68%. Evidence-based methods to increase the return of postal questionnaires were used, including pre-notification cards and non-monetary incentives (individually wrapped tea bags).

Most participants randomised to the intervention group attended an appointment (89%, n = 25/29) and engaged with treatment. Remote delivery of the intervention was viewed positively by participants. Those who had a video call who appreciated being able to see the practitioner, welcoming the human connection and chance to build rapport.

Limitations

Although intervention uptake and engagement with treatment was good, inequalities in access to the internet and electronic devices are an issue in studies that use remote delivery. Some participants experienced delays in obtaining appointment times, which varied by practitioner and site; this may be solved by centralised intervention delivery and offering options such as telephone delivery for those without internet access.

Patients who were eligible at screening but who chose not to take part in the study had better preoperative sleep as measured using the SCI than randomised participants. A common reason for not taking part was not having a sleep

For peer review only - http://bmjopen.bmj.com/site/about/guidelines.xhtml

problem or feeling that treatment would not help. This indicates the SCI eligibility score cutoff would benefit from being lowered.

Study delivery was redesigned to be conducted entirely remotely to meet COVID-19 restrictions, which also influenced the volume of knee replacement operations performed, affecting the number of participants undergoing surgery within the study.

Generally, NHS operation dates cannot be reliably predicted three months in advance, therefore identifying patients at this timepoint proved challenging. Completion of the primary outcome at three months post-surgery was also difficult because many participants did not have their operation within the study timelines. In addition, variations in length of time from randomisation to three months post-surgery would result in high heterogeneity. To address this, the primary outcome assessment for a full trial should not be the proposed outcome of pain three months post-operative, but sleep quality at 14 weeks post-randomisation timepoint for generalisability.

Modifications

There are several key areas to adapt and improve for a future full trial. These include changes to the clinician training programme, including more detailed training on existing sleep interventions and setting SMART goals, streamlined delivery of the intervention by provision of an online portal, and lowering to the screening cut-off for the SCI score.

A review of equality, diversity, and inclusion strategies will ensure a full trial supports inclusivity and engagement from a wide range of communities.

Conclusions

We have demonstrated that a full RCT is feasible based on the predefined progression criteria and have identified areas for improvement to optimise trial design. Recruitment is achievable, engagement with and adherence to the intervention is high and, importantly, the intervention is acceptable to patients and clinicians.

For peer review only - http://bmjopen.bmj.com/site/about/guidelines.xhtml

List of figures and tables

Figure 1: CONSORT participant flow diagram

Table 1: Feasibility outcomes

Table 2: Characteristics of study participants at baseline

Table 3: Data completion rates

Table 4: Patient reported outcome measures

Appendices

Appendix 1: CONSORT 2010 checklist of information to include when reporting a pilot or feasibility randomised trial in a journal

Appendix 2: The TIDieR (Template for Intervention Description and Replication) Checklist

Appendix 3A: Patient interview topic guide

Appendix 3B: ESP intervention training interview topic guide

Appendix 3C: ESP intervention delivery interview topic guide

Appendix 4: Participant outcomes and health economic data

Appendix 5: Demographics of interviewed participants

Appendix 6: Qualitative themes and quotations

Footnotes

Funding

This study is funded by the National Institute for Health and Care Research (NIHR) (Research for Patient Benefit; grant reference number NIHR201036). The research team acknowledges the support of the NIHR, through the Clinical Research Network. This work was also supported by the NIHR Biomedical Research Centre at University Hospitals Bristol and Weston NHS Foundation Trust and the University of Bristol. The views expressed are those of the authors and not necessarily those of the NIHR or the Department of Health and Social Care.

Competing interests: None declared.

Author contributions

KW was Chief Investigator. KW, RGH, VW, CP, AB, AWB, DR, NH, SW and JG were co-applicants on the grant application to NIHR. All authors were involved in the design, delivery and interpretation of the study. WB was the Trial Manager. JG was responsible for the economic analysis. CP was responsible for the statistical analysis. EJ and KW were responsible for the qualitative analysis. WB, KW, CP and JG have access and/or verified the underlying data. WB drafted the manuscript; all authors revised if for important content and approve the final manuscript.

For peer review only

Data sharing

Participants were asked on the consent form if they were willing for their information to be shared anonymously with other researchers to support other research in the future. Anonymised data will be stored on the University of Bristol Research Data Storage Facility (https://data.bris.ac.uk) and will be shared via the University of Bristol Research Data Repository within six months of the publication of the study results. Access to the data will be restricted to ensure that data is only made available to bona fide researchers after a Data Access Agreement has been signed by an institutional signatory.

Ethics

Patient consent for publication: Not applicable

Ethics approval

This study received a favourable opinion from the South West – Cornwall & Plymouth Research Ethics Committee, reference 20/SW/0189, and approval from the Health Research Authority and Health and Care Research Wales (IRAS 289761). Participants gave informed consent to participate in the study before taking part.

Acknowledgements

We would like to thank our patient partners for their help and input, and the research team members and health care professionals who delivered the study including: Gemma Munkenbeck, Leigh Morrison, Paolo Buscemi, Christine Hobson, Giles Head, Lucy Young, Jessica Falatoori, Mat Williams, Chris Dobson and Mark Brand.

The research team acknowledges the support of the NIHR, through the Clinical Research Network. This work was also supported by the NIHR Biomedical Research Centre at University Hospitals Bristol and Weston NHS Foundation Trust and the University of Bristol. The views expressed are those of the authors and not necessarily those of the NIHR or the Department of Health and Social Care.

For peer review only

References

1. Registry, N.J., *National Joint Registry 15th Annual Report 2018.*
2. Project, S.A., *Annual Report 2018*. 2018, NHS National Services Scotland.
3. Gunaratne, R., et al., *Patient Dissatisfaction Following Total Knee Arthroplasty: A Systematic Review of the Literature.* J Arthroplasty, 2017. **32**(12): p. 3854-3860.
4. Beswick, A.D., et al., *What proportion of patients report long-term pain after total hip or knee replacement for osteoarthritis? A systematic review of prospective studies in unselected patients.* BMJ Open, 2012. **2**(1): p. e000435.
5. Hawker, G.A., et al., *The multidimensionality of sleep quality and its relationship to fatigue in older adults with painful osteoarthritis.* Osteoarthritis Cartilage, 2010. **18**(11): p. 1365-71.
6. Wilcox, S., et al., *Factors related to sleep disturbance in older adults experiencing knee pain or knee pain with radiographic evidence of knee osteoarthritis.* J Am Geriatr Soc, 2000. **48**(10): p. 1241-51.
7. Manning, B.T., et al., *Prospective Assessment of Sleep Quality Before and After Primary Total Joint Replacement.* Orthopedics, 2017. **40**(4): p. e636-e640.
8. Bjurstrom, M.F., et al., *Preoperative sleep quality and adverse pain outcomes after total hip arthroplasty.* Eur J Pain, 2021. **25**(7): p. 1482-1492.
9. Smith, T.O., J.R. Dainty, and A.J. MacGregor, *Changes in social isolation and loneliness following total hip and knee arthroplasty: longitudinal analysis of the English Longitudinal Study of Ageing (ELSA) cohort.* Osteoarthritis Cartilage, 2017. **25**(9): p. 1414-1419.
10. Dai, Z., et al., *Sleep Quality Is Related to Worsening Knee Pain in Those with Widespread Pain: The Multicenter Osteoarthritis Study.* J Rheumatol, 2020. **47**(7): p. 1019-1025.
11. Song, J., et al., *Sleep Disturbance Trajectories in Osteoarthritis.* J Clin Rheumatol, 2021. **27**(8): p. e440-e445.
12. Kjolhede, P., et al., *The impact of quality of sleep on recovery from fast-track abdominal hysterectomy.* J Clin Sleep Med, 2012. **8**(4): p. 395-402.
13. Kamdar, B.B., D.M. Needham, and N.A. Collop, *Sleep deprivation in critical illness: its role in physical and psychological recovery.* J Intensive Care Med, 2012. **27**(2): p. 97-111.
14. Hoyle, N.P., et al., *Circadian actin dynamics drive rhythmic fibroblast mobilization during wound healing.* Sci Transl Med, 2017. **9**(415).
15. Wright, K.P., Jr., et al., *Influence of sleep deprivation and circadian misalignment on cortisol, inflammatory markers, and cytokine balance.* Brain Behav Immun, 2015. **47**: p. 24-34.
16. Fadayomi, A.B., et al., *A Systematic Review and Meta-Analysis Examining the Impact of Sleep Disturbance on Postoperative Delirium.* Crit Care Med, 2018. **46**(12): p. e1204-e1212.
17. Roehrs, T.A., et al., *Pain sensitivity and recovery from mild chronic sleep loss.* Sleep, 2012. **35**(12): p. 1667-72.
18. Van Meirhaeghe, J.P., et al., *Improvement in Sleep Patterns After Hip and Knee Arthroplasty: A Prospective Study in 780 Patients.* J Arthroplasty, 2021. **36**(2): p. 442-448.
19. Ding, Z., et al., *Preoperative High Sleep Quality Predicts Further Decrease in Length of Stay after Total Joint Arthroplasty under Enhanced Recovery Short-stay Program: Experience in 604 Patients from a Single Team.* Orthop Surg, 2022. **14**(9): p. 1989-1997.
20. Gong, L., Z. Wang, and D. Fan, *Sleep Quality Effects Recovery After Total Knee Arthroplasty (TKA)--A Randomized, Double-Blind, Controlled Study.* J Arthroplasty, 2015. **30**(11): p. 1897-901.
21. Kirksey, M.A., et al., *Impact of Melatonin on Sleep and Pain After Total Knee Arthroplasty Under Regional Anesthesia With Sedation: A Double-Blind, Randomized, Placebo-Controlled Pilot Study.* J Arthroplasty, 2015. **30**(12): p. 2370-5.
22. Krenk, L., P. Jennum, and H. Kehlet, *Postoperative sleep disturbances after zolpidem treatment in fast-track hip and knee replacement.* J Clin Sleep Med, 2014. **10**(3): p. 321-6.
23. Shen, S.P., et al., *Improved Perioperative Sleep Quality or Quantity Reduces Pain after Total Hip or Knee Arthroplasty: A Systematic Review and Meta-Analysis.* Orthop Surg, 2021. **13**(4): p. 1389-1397.
24. Excellence, N.I.f.H.C.a., *Insomnia: Scenario: Managing long-term insomnia (more than 3 months duration).* 2022.
25. Geenen, R., et al., *EULAR recommendations for the health professional's approach to pain management in inflammatory arthritis and osteoarthritis.* Ann Rheum Dis, 2018. **77**(6): p. 797-807.
26. Koffel, E., et al., *Improving pain and sleep in middle-aged and older adults: the promise of behavioral sleep interventions.* Pain, 2019. **160**(3): p. 529-534.

For peer review only - http://bmjopen.bmj.com/site/about/guidelines.xhtml

27. Whale, K., et al., *The effectiveness of non-pharmacological sleep interventions for people with chronic pain: a systematic review and meta-analysis.* BMC Musculoskeletal Disorders, 2022. **In Press**.
28. Espie, C.A., et al., *The Sleep Condition Indicator: reference values derived from a sample of 200 000 adults.* J Sleep Res, 2018. **27**(3): p. e12643.
29. Craig, P., et al., *Developing and evaluating complex interventions: the new Medical Research Council guidance.* Int J Nurs Stud, 2013. **50**(5): p. 587-92.
30. Whale, K. and R. Gooberman-Hill, *Development of a novel intervention to improve sleep and pain in patients undergoing total knee replacement.* Trials, 2022. **23**(1): p. 625.
31. Kwasnicka, D., et al., *Theoretical explanations for maintenance of behaviour change: a systematic review of behaviour theories.* Health Psychol Rev, 2016. **10**(3): p. 277-96.
32. Orsmond, G.I. and E.S. Cohn, *The Distinctive Features of a Feasibility Study: Objectives and Guiding Questions.* OTJR (Thorofare N J), 2015. **35**(3): p. 169-77.
33. Dawson, J., et al., *Questionnaire on the perceptions of patients about total knee replacement.* J Bone Joint Surg Br, 1998. **80**(1): p. 63-9.
34. Freynhagen, R., et al., *painDETECT: a new screening questionnaire to identify neuropathic components in patients with back pain.* Curr Med Res Opin, 2006. **22**(10): p. 1911-20.
35. Buysse, D.J., et al., *The Pittsburgh Sleep Quality Index: a new instrument for psychiatric practice and research.* Psychiatry Res, 1989. **28**(2): p. 193-213.
36. Zigmond, A.S. and R.P. Snaith, *The hospital anxiety and depression scale.* Acta Psychiatr Scand, 1983. **67**(6): p. 361-70.
37. Herdman, M., et al., *Development and preliminary testing of the new five-level version of EQ-5D (EQ-5D-5L).* Qual Life Res, 2011. **20**(10): p. 1727-36.
38. Al-Janabi, H., T.N. Flynn, and J. Coast, *Development of a self-report measure of capability wellbeing for adults: the ICECAP-A.* Qual Life Res, 2012. **21**(1): p. 167-76.
39. Gale, N.K., et al., *Using the framework method for the analysis of qualitative data in multi-disciplinary health research.* BMC Med Res Methodol, 2013. **13**: p. 117.
40. Tracy, S.J., *Qualitative Quality: Eight "Big-Tent" Criteria for Excellent Qualitative Research.* Qualitative Inquiry, 2010. **16**(10): p. 837-851.
41. Hernandez Alava, M., S. Pudney, and A. Wailoo, *The EQ-5D-5L Value Set for England: Findings of a Quality Assurance Program.* Value Health, 2020. **23**(5): p. 642-648.
42. Pinedo-Villanueva, R., et al., *Identifying individuals with chronic pain after knee replacement: a population-cohort, cluster-analysis of Oxford knee scores in 128,145 patients from the English National Health Service.* BMC Musculoskelet Disord, 2018. **19**(1): p. 354.

For peer review only

Figure 1: CONSORT participant flow diagram

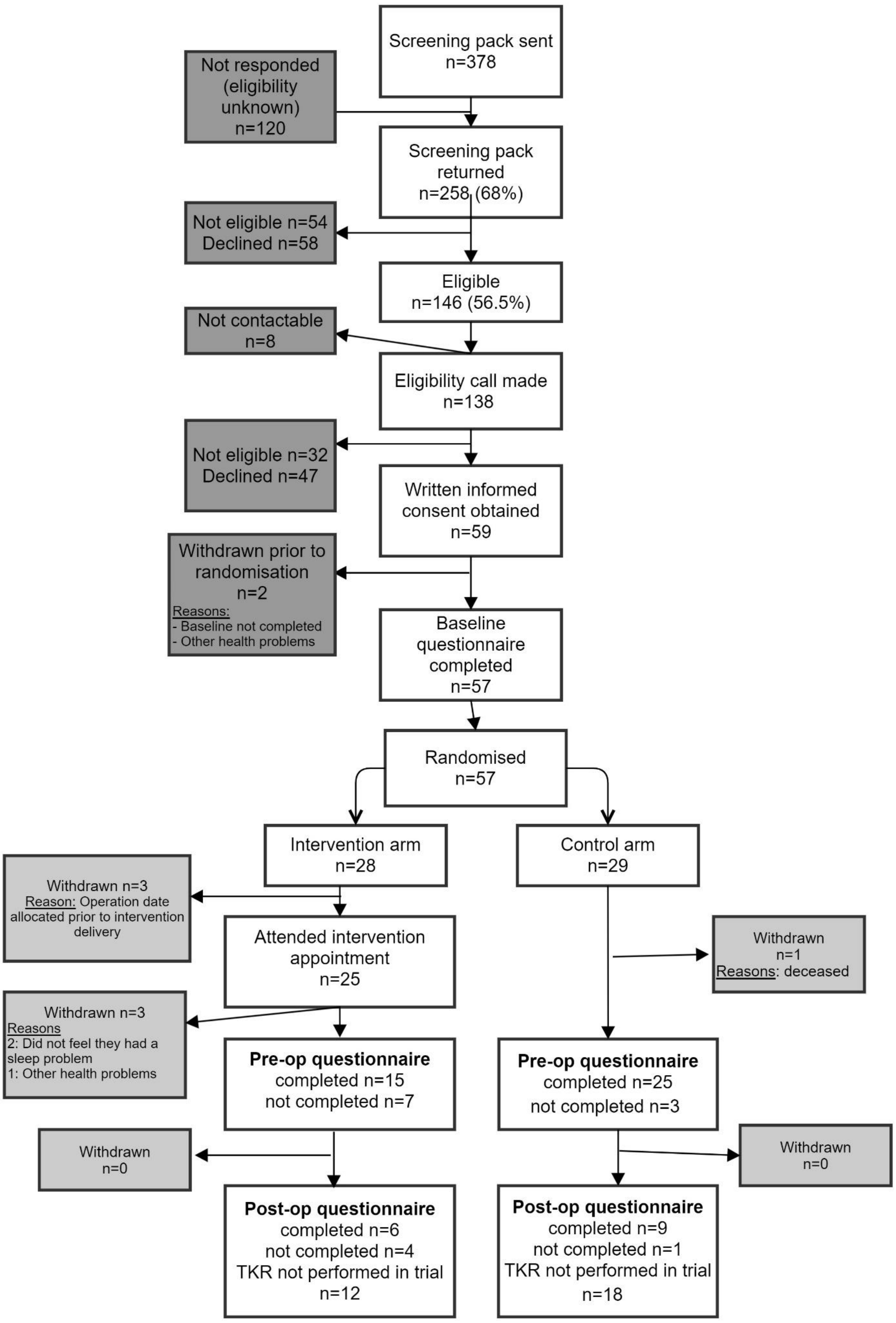

Appendix 1: CONSORT 2010 checklist of information to include when reporting a pilot or feasibility randomised trial in a journal

**Item**	**Description**	**Reported on line number**
Title	Identification of study as randomised pilot or feasibility trial	1
Authors *	Contact details for the corresponding author	9
Trial design	Description of pilot trial design (eg, parallel, cluster)	90
Methods		
Participants	Eligibility criteria for participants and the settings where the pilot trial was conducted	103
Interventions	Interventions intended for each group	122
Objective	Specific objectives of the pilot trial	160
Outcome	Prespecified assessment or measurement to address the pilot trial objectives	169, Table 1
Randomisation	How participants were allocated to interventions	118
Blinding (masking)	Whether or not participants, care givers, and those assessing the outcomes were blinded to group assignment	119
Results		
Numbers randomised	Number of participants screened and randomised to each group for the pilot trial objectives	228
Recruitment	Trial status	N/A
Numbers analysed	Number of participants analysed in each group for the pilot objectives	324, 368
Outcome	Results for the pilot objectives, including any expressions of uncertainty	Objective 1: 228<br>2: 250<br>3: 256<br>4: 271<br>5: 300<br>6: 309<br>7: 315<br>8: 342<br>9: 366
Harms	Important adverse events or side effects	292
Conclusions	General interpretation of the results of pilot trial and their implications for the future definitive trial	448
Trial registration	Registration number for pilot trial and name of trial register	92
Funding	Source of funding for pilot trial	456

Citation: Eldridge SM, Chan CL, Campbell MJ, Bond CM, Hopewell S, Thabane L, et al. CONSORT 2010 statement: extension to randomised pilot and feasibility trials. BMJ. 2016;355.

For peer review only - http://bmjopen.bmj.com/site/about/guidelines.xhtml

Appendix 1: CONSORT 2010 checklist of information to include when reporting a pilot or feasibility trial

Section/Topic	Item No	Checklist item
**Title and abstract**		
	1a	Identification as a pilot or feasibility randomised trial in the title
	1b	Structured summary of pilot trial design, methods, results, and conclusions (for specifi CONSORT abstract extension for pilot trials)
**Introduction**		
Background and objectives	2a	Scientific background and explanation of rationale for future definitive trial, and reasor trial
	2b	Specific objectives or research questions for pilot trial
**Methods**		
Trial design	3a	Description of pilot trial design (such as parallel, factorial) including allocation ratio
	3b	Important changes to methods after pilot trial commencement (such as eligibility criter
Participants	4a	Eligibility criteria for participants
	4b	Settings and locations where the data were collected
	4c	How participants were identified and consented
Interventions	5	The interventions for each group with sufficient details to allow replication, including h actually administered
Outcomes	6a	Completely defined prespecified assessments or measurements to address each pilot 2b, including how and when they were assessed
	6b	Any changes to pilot trial assessments or measurements after the pilot trial commence
	6c	If applicable, prespecified criteria used to judge whether, or how, to proceed with futur
Sample size	7a	Rationale for numbers in the pilot trial
	7b	When applicable, explanation of any interim analyses and stopping guidelines
Randomisation:		
Sequence generation	8a	Method used to generate the random allocation sequence
	8b	Type of randomisation(s); details of any restriction (such as blocking and block size)
Allocation concealment mechanism	9	Mechanism used to implement the random allocation sequence (such as sequentially describing any steps taken to conceal the sequence until interventions were assigned
Implementation	10	Who generated the random allocation sequence, who enrolled participants, and who a interventions
Blinding	11a	If done, who was blinded after assignment to interventions (for example, participants, assessing outcomes) and how
	11b	If relevant, description of the similarity of interventions
Statistical methods	12	Methods used to address each pilot trial objective whether qualitative or quantitative

For peer review only - http://bmjopen.bmj.com/site/about/guidelines.xhtml

For peer review only

**Results**		
Participant flow (a diagram is strongly recommended)	13a	For each group, the numbers of participants who were approached and/or assessed f assigned, received intended treatment, and were assessed for each objective
	13b	For each group, losses and exclusions after randomisation, together with reasons
Recruitment	14a	Dates defining the periods of recruitment and follow-up
	14b	Why the pilot trial ended or was stopped
Baseline data	15	A table showing baseline demographic and clinical characteristics for each group
Numbers analysed	16	For each objective, number of participants (denominator) included in each analysis. If should be by randomised group
Outcomes and estimation	17	For each objective, results including expressions of uncertainty (such as 95% confide estimates. If relevant, these results should be by randomised group
Ancillary analyses	18	Results of any other analyses performed that could be used to inform the future defini
Harms	19	All important harms or unintended effects in each group (for specific guidance see CONS
	19a	If relevant, other important unintended consequences
**Discussion**		
Limitations	20	Pilot trial limitations, addressing sources of potential bias and remaining uncertainty a
Generalisability	21	Generalisability (applicability) of pilot trial methods and findings to future definitive tria
Interpretation	22	Interpretation consistent with pilot trial objectives and findings, balancing potential ben considering other relevant evidence
	22a	Implications for progression from pilot to future definitive trial, including any proposed
**Other information**		
Registration	23	Registration number for pilot trial and name of trial registry
Protocol	24	Where the pilot trial protocol can be accessed, if available
Funding	25	Sources of funding and other support (such as supply of drugs), role of funders
	26	Ethical approval or approval by research review committee, confirmed with reference

Citation: Eldridge SM, Chan CL, Campbell MJ, Bond CM, Hopewell S, Thabane L, et al. CONSORT 2010 statement: extension to randomised pilot and feasibility trials. BMJ. 2016;355.

For peer review only

For peer review only - http://bmjopen.bmj.com/site/about/guidelines.xhtml

Appendix 2: The TIDieR (Template for Intervention Description and Replication) Checklist

TIDieR
Template for Intervention Description and Replication

The TIDieR (Template for Intervention Description and Replication) Checklist*:

Information to include when describing an intervention and the location of the information

REST: A pre-operative tailored sleep intervention for patients undergoing total knee replacement: feasibility study for a randomised controlled trial

Item number	Item	Where located ** Primary paper (page or appendix number)	Other † (details)
	**BRIEF NAME**		
**1.**	Provide the name or a phrase that describes the intervention.	__page 4__	______________
	**WHY**		
**2.**	Describe any rationale, theory, or goal of the elements essential to the intervention.	__page 3_____	______________
	**WHAT**		
**3.**	Materials: Describe any physical or informational materials used in the intervention, including those provided to participants or used in intervention delivery or in training of intervention providers. Provide information on where the materials can be accessed (e.g. online appendix, URL).	__page 4-5__	______________
**4.**	Procedures: Describe each of the procedures, activities, and/or processes used in the intervention, including any enabling or support activities.	__page 4-5___	______________
	**WHO PROVIDED**		
**5.**	For each category of intervention provider (e.g. psychologist, nursing assistant), describe their expertise, background and any specific training given.	__ page 4-5__	______________
	**HOW**		
**6.**	Describe the modes of delivery (e.g. face-to-face or by some other mechanism, such as internet or telephone) of the intervention and whether it was provided individually or in a group.	__ page 4-5__	______________
	**WHERE**		

For peer review only

**7.**	Describe the type(s) of location(s) where the intervention occurred, including any necessary infrastructure or relevant features.	___ page 4-5__	____________
	**WHEN and HOW MUCH**		
**8.**	Describe the number of times the intervention was delivered and over what period of time including the number of sessions, their schedule, and their duration, intensity or dose.	__ page 4-5__	____________
	**TAILORING**		
**9.**	If the intervention was planned to be personalised, titrated or adapted, then describe what, why, when, and how.	___ page 4-5__	____________
	**MODIFICATIONS**		
**10.‡**	If the intervention was modified during the course of the study, describe the changes (what, why, when, and how).	___ N/A____	____________
	**HOW WELL**		
**11.**	Planned: If intervention adherence or fidelity was assessed, describe how and by whom, and if any strategies were used to maintain or improve fidelity, describe them.	___page 8-9__	____________
**12.‡**	Actual: If intervention adherence or fidelity was assessed, describe the extent to which the intervention was delivered as planned.	___page 8-9__	____________

** **Authors** - use N/A if an item is not applicable for the intervention being described. **Reviewers** – use '?' if information about the element is not reported/not sufficiently reported.

† If the information is not provided in the primary paper, give details of where this information is available. This may include locations such as a published protocol or other published papers (provide citation details) or a website (provide the URL).

‡ If completing the TIDieR checklist for a protocol, these items are not relevant to the protocol and cannot be described until the study is complete.

* We strongly recommend using this checklist in conjunction with the TIDieR guide (see *BMJ* 2014;348:g1687) which contains an explanation and elaboration for each item.

* The focus of TIDieR is on reporting details of the intervention elements (and where relevant, comparison elements) of a study. Other elements and methodological features of studies are covered by other reporting statements and checklists and have not been duplicated as part of the TIDieR checklist. When a **randomised trial** is being reported, the TIDieR checklist should be used in conjunction with the CONSORT statement (see www.consort-statement.org) as an extension of **Item 5 of the CONSORT 2010 Statement.** When a **clinical trial protocol** is being reported, the TIDieR checklist should be used in conjunction with the SPIRIT statement as an extension of **Item 11 of the SPIRIT 2013 Statement** (see www.spirit-statement.org). For alternate study designs, TIDieR can be used in conjunction with the appropriate checklist for that study design (see www.equator-network.org).

For peer review only - http://bmjopen.bmj.com/site/about/guidelines.xhtml

Appendix 3A: Patient interview topic guide

REST patient topic guide

Topic guide to be used flexibly. Interviewer will use probes and follow-up questions where appropriate.

Introduction: Discussion of how the interview will be recorded, issues of confidentiality, anonymisation. **Aim of the study:** To understand patient's perspectives of participating in the study, recruitment, and intervention experiences. **Verbal consent procedure**: Consent for interview, **Any questions?**

Introduction / icebreaker

- Thank you for taking part in the interview. To start us off, could you tell me a little about your **knee pain in the lead up towards your surgery** and the **impact that it has had on your sleep?** PROMPT: What problems have you had? Effect on sleep quality/quantity? Impact on daily activities/wellbeing?
- Have you **taken part in any research studies before?** PROMPT: What? When? What was it like taking part?

Design and conduct of the trial (all participants)

- I'd like to talk to you about **when you first heard** about the REST study. Can you tell me how you found out about it?
- What were your **first thoughts** about taking part?
- Can you tell me ***why*** **you decided to take part**? PROMPT: Did you discuss your decision with anyone else, for example friends/family member?
- Did you have **any initial concerns** about being involved in the REST study? PROMPT: Could you tell me about them? What helped you to resolve/overcome these concerns?
- Do you still have **any concerns now**, about either the sleep intervention or your participation in the study itself? PROMPT: Expectations versus reality.
- After you expressed your interest in taking part in the REST study, one of the team would have **given** you **a call to discuss the study** with you in more detail. What was having that conversation like for you? PROMPT: Can you recall what information they told you?
- Overall, **what do you think about the information that you have received about the study**: (i) during the initial telephone call, (ii) in the information booklet. PROMPT: Was there enough information / right kind of information? Was it understandable? Was anything missing?
- **How do you feel about being put randomly into a group** to receive either the sleep treatment or usual care? PROMPT: How acceptable do you feel this is?
- **What do you think about the questionnaires** that you have completed, about your knee pain and sleep? PROMPT: Amount of questions, have the questions been relevant, any sections difficult to complete?

Experiences of the sleep intervention (for intervention group only)

- Now I'd like to talk to you about your experiences of the sleep appointment with [NAME] and the advice you were given during it. Overall, **how did you find the sleep appointment**?
- **What did you think about having a telephone or remote / video appointment**? (If relevant) PROMPT: What was it like for you? Pros and cons, did you have any concerns/problems, what device did you use, familiarity with video conferencing, any support needed (e.g., from family member/friend) with setting it up?
- **How did you find the length of the appointment?** PROMPT: Too long/too short. Too rushed or okay. Enough time to ask questions? Did appointment run to time?

- What **advice did the practitioner give** to you about your sleep during the appointment? PROMPT: What was most/least helpful? What do you think about the way in which they gave this information to you? Was it understandable?
- How did you find **talking about/opening up about your sleep** (problems) with the practitioner?
- Which **sleep treatment option did you choose**? PROMPT: CBT-I, relaxation, mindfulness
- **How involved** did you **feel in choosing** this option? PROMPT: How did the practitioner support you in making this decision? Were you given enough information to help you understand the different options?
- Before taking part in the REST study, did you have any **experience or knowledge of CBT, relaxation or mindfulness** techniques? PROMPT: How do you feel this has influenced your experience in the study?
- **What do you think about the sleep treatment so far**? PROMPT: How are you finding doing it, barriers to engagement, facilitating factors (e.g., partner support), what have you enjoyed/not enjoyed, are you finding it helpful, digital and non-digital options (including ease of use and if any support needed to access digital options), (if appropriate) changing experience over time.
- Did you **make any other changes to your sleep** after the appointment? PROMPT: Sleep aids, apps, sleep hygiene.
- How did you find the **4-week follow up phone call** with the practitioner? PROMPT: What was useful/not useful about it. As a result of the phone call, did you make any changes? If appropriate, use participant's intervention uptake questionnaire to guide questioning.
- Overall, **do you feel the appointment and treatment option has had an impact on your sleep**? PROMPT: What impact has it had? What has had the most impact (e.g. chatting to a professional, increasing knowledge/understanding, engaging with the techniques)?
- **Do you think the sleep appointment and treatment option** you chose has had an **impact on your knee pain**? PROMPT: What impact has it had?
- Would you have liked **any further information or additional support** about your sleep or the treatment you chose? PROMPT: When/how would you have liked to receive this?

For usual care group only

- **Since being recruited** into the REST study, has your **sleep changed**? PROMPT: how has it changed, why, when?
- Have you **tried anything to improve your sleep**? PROMPT: what have you tried, how helpful has it been, when did you try this (i.e. prior to or during the REST study)?
- **Since being recruited** into the REST study, has your **knee pain changed**? PROMPT: how has it changed?

Conclusion (all participants)

- Thinking now about your whole experience of taking part in the REST study, **how could we have improved the way in which the study was organised and run**?
- Do you feel that there are **any ways in which your sleep appointment,** or the **follow up phone call, could have been improved**?
- Is there **anything else** you would like to add/talk about that we haven't covered already?
- **Thank participant for their time**. If appropriate, **signpost** participant **to the 'Useful Contacts' sheet.**
- Ask participant if they would like to receive a **brief report** containing the **key findings** from the interview study.

For peer review only

Appendix 3B: ESP intervention training interview topic guide

REST ESP topic guide – intervention training

Topic guide to be used flexibly. Interviewer will use probes and follow-up questions where appropriate.

Introduction: Discussion of how the interview will be recorded, issues of confidentiality, anonymisation.
Aim of the study: To understand experiences of the training session.
Consent procedure: Check written consent, complete consent form if not already done.

Participant information

- **Year of qualification/Years in practice**
- **Role** at the hospital
- **Experience** of participating in **other trials or research** (PROMPT: any experience with sleep interventions, personal experience)

Background information and general sleep education

- I would like to start by asking you about your **experience/overall impression** of the remote training session. **What did you think about the session in general?** (PROMPT: What did you like? What did you not like? What did you enjoy/not enjoy? One long session vs two shorter over two days?)
- Did you **prepare** in any way for the session? (PROMPT: background reading)
- Thinking now about the **information** that you were given **on sleep** and **why sleep is important**:
  - **How understandable** was it?
  - **How useful** did you find it?
- Is there any **additional background information** that you would have **liked to have received** during the training?
- **How confident are you feeling now** about **talking about the background information** with the trial participants? (PROMPT: if not, what additional support / information would you like? How would you like to receive this?)

Appointment delivery

- How do you **plan to structure** your **sessions**? (PROMPT: Recommended timings)

Assessment

- What is your **understanding of the assessment process**?
- Do you **feel clear / confident** about **how to carry out the assessment**? (PROMPT: Using the assessment tool)
- **How do you feel** about **eliciting this information** from the participant?
- What did you think about the **assessment role-play exercise**? (PROMPT: What did you learn from it? How helpful/useful did you find it? Any suggestions about how it could have been done differently? Did you find time to practice the role play exercise after the training?)
- What do you think will be the **main challenges with the assessment process**?

For peer review only

Intervention delivery

- **What did you think** about the **sleep hygiene and education information**? (PROMPT: What did you like/What didn't you like?)
- Is it **clear how to tailor advice** to each participant? (PROMPT: Using the assessment table)
- **What did you think** about the **information** you received **on the specific sleep interventions** you will be recommending?
- **How will you choose** which sleep **intervention** is **most appropriate** for a participant?
- What is your **understanding of the behavioural contract**? (PROMPT: Confident using it, how to choose which areas to highlight, purpose of contract, setting SMART goals)
- What do you think will be **the main challenges with** the **intervention delivery**?

Conclusion

- **How are you feeling about** your **first appointment**? (PROMPT: Do you feel prepared? Is there anything that you will do between now and then to feel more prepared? Is there anything that you are still not sure about/want to know?)
- Is there **any information that you would have liked** in the training that **wasn't provided**?
- Was there **any information** in the training, which you felt was **not useful / needed**?
- Do you have any **suggestions on how to improve the training**?
- Overall, what did you find **most useful** about the training?
- What do you **think about the training manual**? Any **suggestions for improvement**?
- What were **the advantages and disadvantages** of doing the **training over Zoom**?
- Is there **anything else** that you would like to add, or anything you wish to talk about that we haven't covered already?
- **Thank you** for talking to me/your time/**when will be in contact again**.

END

*** Ask ESP about possibility of observing some of their intervention appointments with patients who have <u>not</u> consented to take part in an interview – in order to learn more about the process/patient experience***

For peer review only

Appendix 3C: ESP intervention delivery interview topic guide

REST ESP topic guide – intervention delivery

***Introduction**: Discussion of how the interview will be recorded, issues of confidentiality, anonymisation.*
***Aim of the study**: To understand experiences of delivering the intervention and any additional training needs.*
***Verbal consent procedure**: Reaffirm consent for interview (written consent already given).*

Preparation for delivery

When we first spoke, you told me that before your first intervention appointment, you planned to do [XX]. Did you do this? Did you do anything else to prepare for the appointments? How did undertaking these activities help you to feel more prepared/confident?

Intervention appointment

- **How many sessions** delivered/mode of delivery and **overall experience of delivering** the **sessions: main challenges - for them & patients** (e.g., pragmatic challenges - timing/contacting patients/accessing Zoom/meeting remotely) - & **how overcome**
- **How have you found** undertaking the **assessment process and challenges**? (e.g., Know what questions to ask, eliciting the right information, following the assessment table, drawing out info from patients about sleep issues, patient engagement)
- **How** do you **go about choosing** which **sleep intervention** is best for a participant and any **challenges**? (e.g., selecting most appropriate intervention; shared decision making; patients choosing alternative option)
- **Experience of agreeing the behavioural contract**? (How do you decide on what to include, do participants engage, do you think it is helpful)
- How has the way in which **you deliver the intervention appointment changed over time**? (Refinements made. Increased confidence over time?)
- Impact of mode of delivery on patient engagement/disclosure
- What kind of **questions** have **participants asked** you during their assessment appointments? (Have you felt that you have had the appropriate knowledge/skills to address their questions?)
- **What outcomes would define a successful appointment** – for you and for the participant?

Sleep intervention set up and referral procedure

- **Experience of setting up the interventions** (e.g. clear what to do/what information needed) **& challenges** (e.g. free Headspace trial already used? how was this managed/what did they recommend)
- **Sleepstation referral process** (Any challenges, how long has it taken)
- **Information and support participants want** about **getting started with the sleep interventions**? (Able to give patients the support/info they needed? Paper versions of the documents requested?)

Follow-up phone call

- **How have you found** doing the **4-week follow-up phone calls**? (Challenges getting hold of patients, any

Conclusion

- **Additional training or information needs**? (How/When/Why)
- Thoughts on how **training itself, manual** and **documents could be improved**? (i.e. changes needed)
- **Recommendations for refinements** needed to improve way in which the intervention is delivered? (What do you think has worked well? What hasn't worked so well?)
- How has being part of REST/your REST role **benefited you either personally or professionally**?

***Explore confidence around setting the SMART goals

***Do they feel they understand the theory behind the different interventions and are they able to communicate it to patients?

***Do they feel that they have a good understanding of what SleepStation involves for a patient and are they able to/do they communicate this to patients?

***Have they kept a reflective diary?

For peer review only

Appendix 4: Participant outcomes and health economic data

Comparison of SCI between patients eligible at screening and randomized trial participants

Characteristic	In trial, N = 57[1]	Not in trial, N = 201[1]	p-value[2]
**SCI_Score**	13 (8, 17)	17 (10, 26)	0.004
Unknown	0	49	
[1]Median (IQR)			
[2]Wilcoxon rank sum test			

Pain outcomes at 3 months postoperative (Oxford Knee Score)

Characteristic	Intervention, N = 6[1]	95% CI[2]	Control, N = 9[1]	95% CI[2]
**On-going pain (OKS<14)**				
No on-going pain	5 (83%)	36%, 99%	8 (100%)	60%, 100%
On-going pain	1 (17%)	0.88%, 64%	0 (0%)	0.00%, 40%
Unknown	0		1	
[1]n (%)				
[2]CI = Confidence Interval				

Neuropathic pain outcomes at 3 months postoperative

	**Baseline**		**Pre-operative**		**Post-operative**	
**Characteristic**	Intervention, N = 28[1]	Control, N = 29[1]	Intervention, N = 15[1]	Control, N = 25[1]	Intervention, N = 6[1]	Control, N = 9[1]
**PainDETECT score**	18 (11, 21)	13 (9, 19)	11 (10, 17)	14 (10, 24)	7 (6, 12)	10 (6, 16)
	4	4	6	5	2	3
**PainDETECT category**						
Ambiguous	6 (25%)	5 (20%)	3 (33%)	5 (25%)	0 (0%)	1 (17%)
Neuropathic likely	9 (38%)	8 (32%)	1 (11%)	7 (35%)	1 (25%)	1 (17%)
Nociceptive	9 (38%)	12 (48%)	5 (56%)	8 (40%)	3 (75%)	4 (67%)
Unknown	4	4	6	5	2	3
[1]Median (IQR); n (%)						

EQ-5D-5L and ICECAP scores

Measure	Baseline			Pre-operation			Post-operation		
	Intervention (SD)	Usual care (SD)	Response	Intervention (SD)	Usual care (SD)	Response	Intervention (SD)	Usual care (SD)	Response
EQ-5D-5L	**0.34** (0.30)	**0.47** (0.21)	57/57	**0.35** (0.22)	**0.40** (0.23)	40/57	**0.63** (0.24)	**0.69** (0.13)	14/27*
ICECAP	**0.71** (0.26)	**0.78** (0.14)	56/57	**0.71** (0.23)	**0.80** (0.14)	40/57	**0.83** (0.25)	**0.91** (0.021)	15/27*

*Only collected for those who had TKR within trial.

Intervention costs

Treatment Cost per person	Mean	Max	Min
Staff	£103.73	£214.50	£33.00
NHS Treatment cost[§]	30.72	£61.45	£12.29
Societal treatment cost[¥]	£6.59	£19.78	£0
NHS Total (per person)	£134.45	£275.95	£45.29
Societal Total (per person)	£141.04	£295.73	£45.29

[§] Mean - 50% discount given to NHS, Max -no discount, Min - 80% discount offered to NHS.
[¥] Mean -1/3 of participants paid for Headspace and Calm app subscriptions, Max all pay for subscriptions, Min- no one pays for subscription.
*https://www.nice.org.uk/process/pmg36/chapter/economic-evaluation

For peer review only

Appendix 5: Demographics of interviewed participants

Pseudonym	Gender	Age at interview	Intervention or Usual Care	Mode of intervention delivery	Chosen sleep intervention
Florence	Woman	90	Intervention	Telephone	Relaxation
George	Man	64	Usual	N/A	N/A
Joyce	Woman	73	Usual	N/A	N/A
Gail	Woman	64	Usual	N/A	N/A
Charles	Man	79	Intervention	Video	CBT-I
Patricia	Woman	69	Intervention	Video	CBT-I
Gloria	Woman	73	Intervention	Video	Mindfulness
Ruth	Woman	72	Intervention	Video	CBT-I
Arthur	Man	72	Usual	N/A	N/A
Edward	Man	69	Intervention	Telephone	Mindfulness
Jerry	Man	64	Intervention	Video	Relaxation
Steven	Man	58	Usual	N/A	N/A
Rose	Woman	68	Intervention	Video	Mindfulness then CBT-I

For peer review only

Appendix 6: Qualitative themes and quotations

Theme	Subtheme	Quotations
**3. Explore engagement with the REST intervention and adherence to the agreed sleep plan**	Practitioner manner and communication style	*Well I think I was speaking to a professional and when I feel that, that gives me a little confidence in talking to her […] and I found that with [the practitioner] she was really very patient and in discussing her ideas […] she was very encouraging actually …[Florence]*<br>*It's been good. Just talk about things and get it into the open, don't bottle everything up [...] it suits me fine. [Jerry]*<br><br>*Well she was quite firm I felt in telling me about relaxing and how I would relax and she really wanted me to make up my mind then and there and tell her what I was planning to do and that made me feel more positive about it [...] she made me discuss it and having done that I was able to make my own mind up… she was suggesting breathing exercises as well […] there was no insistence at all from her, but on the other hand she was very firm in her suggestions [yes] which made me feel that I must do those things [Florence]*
**1. Evaluate intervention acceptability to patients and health professionals**	Patient acceptability: delivery mode	*I'm using Zoom with family etcetera, so I've used it for over a year now and I'm happy with it […] I'm confident using it [Patricia]*<br><br>*I'm not good on this Wi-Fi stuff, I'm really quite awful on it and my poor son was trying to train me [...] I don't know how to cope or how to use anything with Wi-Fi, I find it very irksome, I really do, very overwhelmingly worrying, I just worry about it. [Florence]*<br><br>*I think this sort of appointment you don't need to be face-to-face. […] There's no point doing face-to-face. Yes, it would be a 50-mile round trip for me to come to you […] It's fine seeing each other like this to be honest. [...] Travel time, parking, petrol. It's saving a fair bit of money from my point of view. [Patricia]*<br><br>*I don't see a disadvantage. Sitting in my own home rather than going to a hospital where I could pick up more diseases or COVID again. [Gloria]*
	Patient acceptability: appointment length and structure	*There was somebody there who could give me suggestions on the way whilst waiting for my operation, do you know what I mean? It's almost as though she was giving me something to do and think about as I approached the operation … [Florence]*

For peer review only

		The phone call took place not at the right time. But that was fine by me because I wasn't doing anything else. [Patricia]<br><br>*I'm not pleased, I've taken time off work to do this, but I was happy to take time off work to do it but not to be able to do it... [Ruth]*
	Practitioner acceptability: mode of delivery	*I'd be the first to admit that my IT is not as good as it should. I don't do much in the way of IT. [...] I'm not the slickest at getting it all up on the computer. [...] I find the IT thing a bit of a challenge sometimes [ESP03].*<br><br>*They've all been there ready and waiting and a few times, I've been late finishing the morning clinic because I normally do them on a Monday afternoon. They've all been ready and waiting at their phone or their computer. [ESP03]*
	Practitioner acceptability: intervention handbook and paperwork	*The form was very easy to follow for all the questions [ESP01]*<br><br>*The actual booklet that you're given yes, that's easy. I think maybe I probably need to introduce it a little bit better and what the study is, but then the actual questions are easy to follow, and then it follows onto the SMART goals and things, yes. [ESP01]*<br><br>*I think overall fine, I think sometimes you do feel like you're duplicating quite a lot I think, and when I was actually working through paperwork too quickly and not really sitting down and really going in depth about what the question's asking. Sometimes it feels like, well see above, kind of thing. Maybe that's because I'm classically the kind of person that will write everything down in the first box [RE 4-week call paperwork] [ESP04]*
**6. Evaluate the acceptability of randomisation**		*I understand that that's the only way you can gauge whether what you're doing is of any benefit if it's all randomised. You're not picking out a group of people that are better than another group of people in terms of their symptoms. [Joyce]*<br><br>*I would have particularly liked it if I was one of the people that was offered sort of help with sleep and so on, because the ideal for me would be to find an alternative to running through the highs and lows of my life at three o'clock in the morning would be good. So I was a bit disappointed. [Arthur]*
**9. Collect data on patient-reported outcomes measures to assess data**		*Things affect you differently at different times [...] some days, if you're feeling really well, I think you fill it in through rose-tinted glasses. That's the only way I can describe it. If you were having a really good day*

For peer review only

**completion rates and inform the selection of the primary outcome measure and sample size for a full trial**		*and things were going well, you'd fill it in a little bit differently … [Joyce]*

For peer review only

For peer review only

Appendix 1: CONSORT 2010 checklist of information to include when reporting a pilot or feasibility randomised trial in a journal

Item	Description	Reported on line number
Title	Identification of study as randomised pilot or feasibility trial	1
Authors *	Contact details for the corresponding author	9
Trial design	Description of pilot trial design (eg, parallel, cluster)	90
Methods		
Participants	Eligibility criteria for participants and the settings where the pilot trial was conducted	103
Interventions	Interventions intended for each group	122
Objective	Specific objectives of the pilot trial	160
Outcome	Prespecified assessment or measurement to address the pilot trial objectives	169, Table 1
Randomisation	How participants were allocated to interventions	118
Blinding (masking)	Whether or not participants, care givers, and those assessing the outcomes were blinded to group assignment	119
Results		
Numbers randomised	Number of participants screened and randomised to each group for the pilot trial objectives	228
Recruitment	Trial status	N/A
Numbers analysed	Number of participants analysed in each group for the pilot objectives	324, 368
Outcome	Results for the pilot objectives, including any expressions of uncertainty	Objective 1: 228<br>2: 250<br>3: 256<br>4: 271<br>5: 300<br>6: 309<br>7: 315<br>8: 342<br>9: 366
Harms	Important adverse events or side effects	292
Conclusions	General interpretation of the results of pilot trial and their implications for the future definitive trial	448
Trial registration	Registration number for pilot trial and name of trial register	92
Funding	Source of funding for pilot trial	456

Citation: Eldridge SM, Chan CL, Campbell MJ, Bond CM, Hopewell S, Thabane L, et al. CONSORT 2010 statement: extension to randomised pilot and feasibility trials. BMJ. 2016;355.

For peer review only - http://bmjopen.bmj.com/site/about/guidelines.xhtml

Appendix 1: CONSORT 2010 checklist of information to include when reporting a pilot or feasibility trial

Section/Topic	Item No	Checklist item	Reported on page No
**Title and abstract**			
	1a	Identification as a pilot or feasibility randomised trial in the title	1
	1b	Structured summary of pilot trial design, methods, results, and conclusions (for specific guidance see CONSORT abstract extension for pilot trials)	2
**Introduction**			
Background and objectives	2a	Scientific background and explanation of rationale for future definitive trial, and reasons for randomised pilot trial	3
	2b	Specific objectives or research questions for pilot trial	5, Table 1
**Methods**			
Trial design	3a	Description of pilot trial design (such as parallel, factorial) including allocation ratio	3
	3b	Important changes to methods after pilot trial commencement (such as eligibility criteria), with reasons	N/A
Participants	4a	Eligibility criteria for participants	4
	4b	Settings and locations where the data were collected	3, 4
	4c	How participants were identified and consented	4
Interventions	5	The interventions for each group with sufficient details to allow replication, including how and when they were actually administered	4
Outcomes	6a	Completely defined prespecified assessments or measurements to address each pilot trial objective specified in 2b, including how and when they were assessed	5
	6b	Any changes to pilot trial assessments or measurements after the pilot trial commenced, with reasons	N/A
	6c	If applicable, prespecified criteria used to judge whether, or how, to proceed with future definitive trial	5, 6, Table 1
Sample size	7a	Rationale for numbers in the pilot trial	6
	7b	When applicable, explanation of any interim analyses and stopping guidelines	N/A
Randomisation:			
Sequence generation	8a	Method used to generate the random allocation sequence	4
	8b	Type of randomisation(s); details of any restriction (such as blocking and block size)	4
Allocation concealment mechanism	9	Mechanism used to implement the random allocation sequence (such as sequentially numbered containers), describing any steps taken to conceal the sequence until interventions were assigned	4
Implementation	10	Who generated the random allocation sequence, who enrolled participants, and who assigned participants to interventions	4
Blinding	11a	If done, who was blinded after assignment to interventions (for example, participants, care providers, those assessing outcomes) and how	4
	11b	If relevant, description of the similarity of interventions	N/A
Statistical methods	12	Methods used to address each pilot trial objective whether qualitative or quantitative	6

For peer review only

**Results**			
Participant flow (a diagram is strongly recommended)	13a	For each group, the numbers of participants who were approached and/or assessed for eligibility, randomly assigned, received intended treatment, and were assessed for each objective	6, 7
	13b	For each group, losses and exclusions after randomisation, together with reasons	7, 10, Figure 1
Recruitment	14a	Dates defining the periods of recruitment and follow-up	
	14b	Why the pilot trial ended or was stopped	N/A
Baseline data	15	A table showing baseline demographic and clinical characteristics for each group	Table 2
Numbers analysed	16	For each objective, number of participants (denominator) included in each analysis. If relevant, these numbers should be by randomised group	6-10, Table 4
Outcomes and estimation	17	For each objective, results including expressions of uncertainty (such as 95% confidence interval) for any estimates. If relevant, these results should be by randomised group	10, Table 3, 4
Ancillary analyses	18	Results of any other analyses performed that could be used to inform the future definitive trial	
Harms	19	All important harms or unintended effects in each group (for specific guidance see CONSORT for harms)	8
	19a	If relevant, other important unintended consequences	N/A
**Discussion**			
Limitations	20	Pilot trial limitations, addressing sources of potential bias and remaining uncertainty about feasibility	10
Generalisability	21	Generalisability (applicability) of pilot trial methods and findings to future definitive trial and other studies	10-12
Interpretation	22	Interpretation consistent with pilot trial objectives and findings, balancing potential benefits and harms, and considering other relevant evidence	10-12
	22a	Implications for progression from pilot to future definitive trial, including any proposed amendments	10-12
**Other information**			
Registration	23	Registration number for pilot trial and name of trial registry	3
Protocol	24	Where the pilot trial protocol can be accessed, if available	N/A
Funding	25	Sources of funding and other support (such as supply of drugs), role of funders	13
	26	Ethical approval or approval by research review committee, confirmed with reference number	13

Citation: Eldridge SM, Chan CL, Campbell MJ, Bond CM, Hopewell S, Thabane L, et al. CONSORT 2010 statement: extension to randomised pilot and feasibility trials. BMJ. 2016;355.

For peer review only - http://bmjopen.bmj.com/site/about/guidelines.xhtml

Appendix 2: The TIDieR (Template for Intervention Description and Replication) Checklist

TIDieR Template for Intervention Description and Replication

The TIDieR (Template for Intervention Description and Replication) Checklist*:

Information to include when describing an intervention and the location of the information

REST: A pre-operative tailored sleep intervention for patients undergoing total knee replacement: feasibility study for a randomised controlled trial

Item number	Item	Where located ** Primary paper (page or appendix number)	Other † (details)
	**BRIEF NAME**		
1.	Provide the name or a phrase that describes the intervention.	page 4	______
	**WHY**		
2.	Describe any rationale, theory, or goal of the elements essential to the intervention.	page 3	______
	**WHAT**		
3.	Materials: Describe any physical or informational materials used in the intervention, including those provided to participants or used in intervention delivery or in training of intervention providers. Provide information on where the materials can be accessed (e.g. online appendix, URL).	page 4-5	______
4.	Procedures: Describe each of the procedures, activities, and/or processes used in the intervention, including any enabling or support activities.	page 4-5	______
	**WHO PROVIDED**		
5.	For each category of intervention provider (e.g. psychologist, nursing assistant), describe their expertise, background and any specific training given.	page 4-5	______
	**HOW**		
6.	Describe the modes of delivery (e.g. face-to-face or by some other mechanism, such as internet or telephone) of the intervention and whether it was provided individually or in a group.	page 4-5	______
	**WHERE**		

For peer review only

7.	Describe the type(s) of location(s) where the intervention occurred, including any necessary infrastructure or relevant features.	page 4-5	
	**WHEN and HOW MUCH**		
8.	Describe the number of times the intervention was delivered and over what period of time including the number of sessions, their schedule, and their duration, intensity or dose.	page 4-5	
	**TAILORING**		
9.	If the intervention was planned to be personalised, titrated or adapted, then describe what, why, when, and how.	page 4-5	
	**MODIFICATIONS**		
10.‡	If the intervention was modified during the course of the study, describe the changes (what, why, when, and how).	N/A	
	**HOW WELL**		
11.	Planned: If intervention adherence or fidelity was assessed, describe how and by whom, and if any strategies were used to maintain or improve fidelity, describe them.	page 8-9	
12.‡	Actual: If intervention adherence or fidelity was assessed, describe the extent to which the intervention was delivered as planned.	page 8-9	

** **Authors** - use N/A if an item is not applicable for the intervention being described. **Reviewers** – use '?' if information about the element is not reported/not sufficiently reported.

† If the information is not provided in the primary paper, give details of where this information is available. This may include locations such as a published protocol or other published papers (provide citation details) or a website (provide the URL).

‡ If completing the TIDieR checklist for a protocol, these items are not relevant to the protocol and cannot be described until the study is complete.

* We strongly recommend using this checklist in conjunction with the TIDieR guide (see *BMJ* 2014;348:g1687) which contains an explanation and elaboration for each item.

* The focus of TIDieR is on reporting details of the intervention elements (and where relevant, comparison elements) of a study. Other elements and methodological features of studies are covered by other reporting statements and checklists and have not been duplicated as part of the TIDieR checklist. When a **randomised trial** is being reported, the TIDieR checklist should be used in conjunction with the CONSORT statement (see www.consort-statement.org) as an extension of **Item 5 of the CONSORT 2010 Statement.** When a **clinical trial protocol** is being reported, the TIDieR checklist should be used in conjunction with the SPIRIT statement as an extension of **Item 11 of the SPIRIT 2013 Statement** (see www.spirit-statement.org). For alternate study designs, TIDieR can be used in conjunction with the appropriate checklist for that study design (see www.equator-network.org).

For peer review only

Appendix 3A: Patient interview topic guide

REST patient topic guide

Topic guide to be used flexibly. Interviewer will use probes and follow-up questions where appropriate.

Introduction: Discussion of how the interview will be recorded, issues of confidentiality, anonymisation. **Aim of the study:** To understand patient's perspectives of participating in the study, recruitment, and intervention experiences. **Verbal consent procedure**: Consent for interview, **Any questions?**

Introduction / icebreaker

- Thank you for taking part in the interview. To start us off, could you tell me a little about your **knee pain in the lead up towards your surgery** and the **impact that it has had on your sleep?** PROMPT: What problems have you had? Effect on sleep quality/quantity? Impact on daily activities/wellbeing?
- Have you **taken part in any research studies before?** PROMPT: What? When? What was it like taking part?

Design and conduct of the trial (all participants)

- I'd like to talk to you about **when you first heard** about the REST study. Can you tell me how you found out about it?
- What were your **first thoughts** about taking part?
- Can you tell me ***why* you decided to take part**? PROMPT: Did you discuss your decision with anyone else, for example friends/family member?
- Did you have **any initial concerns** about being involved in the REST study? PROMPT: Could you tell me about them? What helped you to resolve/overcome these concerns?
- Do you still have **any concerns now**, about either the sleep intervention or your participation in the study itself? PROMPT: Expectations versus reality.
- After you expressed your interest in taking part in the REST study, one of the team would have **given** you **a call to discuss the study** with you in more detail. What was having that conversation like for you? PROMPT: Can you recall what information they told you?
- Overall, **what do you think about the information that you have received about the study**: (i) during the initial telephone call, (ii) in the information booklet. PROMPT: Was there enough information / right kind of information? Was it understandable? Was anything missing?
- **How do you feel about being put randomly into a group** to receive either the sleep treatment or usual care? PROMPT: How acceptable do you feel this is?
- **What do you think about the questionnaires** that you have completed, about your knee pain and sleep? PROMPT: Amount of questions, have the questions been relevant, any sections difficult to complete?

Experiences of the sleep intervention (for intervention group only)

- Now I'd like to talk to you about your experiences of the sleep appointment with [NAME] and the advice you were given during it. Overall, **how did you find the sleep appointment**?
- **What did you think about having a telephone or remote / video appointment**? (If relevant) PROMPT: What was it like for you? Pros and cons, did you have any concerns/problems, what device did you use, familiarity with video conferencing, any support needed (e.g., from family member/friend) with setting it up?
- **How did you find the length of the appointment?** PROMPT: Too long/too short. Too rushed or okay. Enough time to ask questions? Did appointment run to time?

For peer review only

- What **advice did the practitioner give** to you about your sleep during the appointment? PROMPT: What was most/least helpful? What do you think about the way in which they gave this information to you? Was it understandable?
- How did you find **talking about/opening up about your sleep** (problems) with the practitioner?
- Which **sleep treatment option did you choose**? PROMPT: CBT-I, relaxation, mindfulness
- **How involved** did you **feel in choosing** this option? PROMPT: How did the practitioner support you in making this decision? Were you given enough information to help you understand the different options?
- Before taking part in the REST study, did you have any **experience or knowledge of CBT, relaxation or mindfulness** techniques? PROMPT: How do you feel this has influenced your experience in the study?
- **What do you think about the sleep treatment so far**? PROMPT: How are you finding doing it, barriers to engagement, facilitating factors (e.g., partner support), what have you enjoyed/not enjoyed, are you finding it helpful, digital and non-digital options (including ease of use and if any support needed to access digital options), (if appropriate) changing experience over time.
- Did you **make any other changes to your sleep** after the appointment? PROMPT: Sleep aids, apps, sleep hygiene.
- How did you find the **4-week follow up phone call** with the practitioner? PROMPT: What was useful/not useful about it. As a result of the phone call, did you make any changes? If appropriate, use participant's intervention uptake questionnaire to guide questioning.
- Overall, **do you feel the appointment and treatment option has had an impact on your sleep**? PROMPT: What impact has it had? What has had the most impact (e.g. chatting to a professional, increasing knowledge/understanding, engaging with the techniques)?
- **Do you think the sleep appointment and treatment option** you chose has had an **impact on your knee pain**? PROMPT: What impact has it had?
- Would you have liked **any further information or additional support** about your sleep or the treatment you chose? PROMPT: When/how would you have liked to receive this?

For usual care group only

- **Since being recruited** into the REST study, has your **sleep changed**? PROMPT: how has it changed, why, when?
- Have you **tried anything to improve your sleep**? PROMPT: what have you tried, how helpful has it been, when did you try this (i.e. prior to or during the REST study)?
- **Since being recruited** into the REST study, has your **knee pain changed**? PROMPT: how has it changed?

Conclusion (all participants)

- Thinking now about your whole experience of taking part in the REST study, **how could we have improved the way in which the study was organised and run**?
- Do you feel that there are **any ways in which your sleep appointment,** or the **follow up phone call, could have been improved**?
- Is there **anything else** you would like to add/talk about that we haven't covered already?
- **Thank participant for their time**. If appropriate, **signpost** participant **to the 'Useful Contacts' sheet.**
- Ask participant if they would like to receive a **brief report** containing the **key findings** from the interview study.

For peer review only

Appendix 3B: ESP intervention training interview topic guide

REST ESP topic guide – intervention training

Topic guide to be used flexibly. Interviewer will use probes and follow-up questions where appropriate.

Introduction: Discussion of how the interview will be recorded, issues of confidentiality, anonymisation.
Aim of the study: To understand experiences of the training session.
Consent procedure: Check written consent, complete consent form if not already done.

Participant information

- **Year of qualification/Years in practice**
- **Role** at the hospital
- **Experience** of participating in **other trials or research** (PROMPT: any experience with sleep interventions, personal experience)

Background information and general sleep education

- I would like to start by asking you about your **experience/overall impression** of the remote training session. **What did you think about the session in general?** (PROMPT: What did you like? What did you not like? What did you enjoy/not enjoy? One long session vs two shorter over two days?)
- Did you **prepare** in any way for the session? (PROMPT: background reading)
- Thinking now about the **information** that you were given **on sleep** and **why sleep is important**:
  - **How understandable** was it?
  - **How useful** did you find it?
- Is there any **additional background information** that you would have **liked to have received** during the training?
- **How confident are you feeling now** about **talking about the background information** with the trial participants? (PROMPT: if not, what additional support / information would you like? How would you like to receive this?)

Appointment delivery

- How do you **plan to structure** your **sessions**? (PROMPT: Recommended timings)

Assessment

- What is your **understanding of the assessment process**?
- Do you **feel clear / confident** about **how to carry out the assessment**? (PROMPT: Using the assessment tool)
- **How do you feel** about **eliciting this information** from the participant?
- What did you think about the **assessment role-play exercise**? (PROMPT: What did you learn from it? How helpful/useful did you find it? Any suggestions about how it could have been done differently? Did you find time to practice the role play exercise after the training?)
- What do you think will be the **main challenges with the assessment process**?

For peer review only

Intervention delivery

- **What did you think** about the **sleep hygiene and education information**? (PROMPT: What did you like/What didn't you like?)
- Is it **clear how to tailor advice** to each participant? (PROMPT: Using the assessment table)
- **What did you think** about the **information** you received **on the specific sleep interventions** you will be recommending?
- **How will you choose** which sleep **intervention** is **most appropriate** for a participant?
- What is your **understanding of the behavioural contract**? (PROMPT: Confident using it, how to choose which areas to highlight, purpose of contract, setting SMART goals)
- What do you think will be **the main challenges with** the **intervention delivery**?

Conclusion

- **How are you feeling about** your **first appointment**? (PROMPT: Do you feel prepared? Is there anything that you will do between now and then to feel more prepared? Is there anything that you are still not sure about/want to know?)
- Is there **any information that you would have liked** in the training that **wasn't provided**?
- Was there **any information** in the training, which you felt was **not useful / needed**?
- Do you have any **suggestions on how to improve the training**?
- Overall, what did you find **most useful** about the training?
- What do you **think about the training manual**? Any **suggestions for improvement**?
- What were **the advantages and disadvantages** of doing the **training over Zoom**?
- Is there **anything else** that you would like to add, or anything you wish to talk about that we haven't covered already?
- **Thank you** for talking to me/your time/**when will be in contact again**.

END

*** Ask ESP about possibility of observing some of their intervention appointments with patients who have not consented to take part in an interview – in order to learn more about the process/patient experience***

For peer review only

Appendix 3C: ESP intervention delivery interview topic guide

REST ESP topic guide – intervention delivery

***Introduction**: Discussion of how the interview will be recorded, issues of confidentiality, anonymisation.*
***Aim of the study**: To understand experiences of delivering the intervention and any additional training needs.*
***Verbal consent procedure**: Reaffirm consent for interview (written consent already given).*

Preparation for delivery

When we first spoke, you told me that before your first intervention appointment, you planned to do [XX]. Did you do this? Did you do anything else to prepare for the appointments? How did undertaking these activities help you to feel more prepared/confident?

Intervention appointment

- **How many sessions** delivered/mode of delivery and **overall experience of delivering** the **sessions: main challenges - for them & patients** (e.g., pragmatic challenges - timing/contacting patients/accessing Zoom/meeting remotely) - & **how overcome**
- **How have you found** undertaking the **assessment process and challenges**? (e.g., Know what questions to ask, eliciting the right information, following the assessment table, drawing out info from patients about sleep issues, patient engagement)
- **How** do you **go about choosing** which **sleep intervention** is best for a participant and any **challenges**? (e.g., selecting most appropriate intervention; shared decision making; patients choosing alternative option)
- **Experience of agreeing the behavioural contract**? (How do you decide on what to include, do participants engage, do you think it is helpful)
- How has the way in which **you deliver the intervention appointment changed over time**? (Refinements made. Increased confidence over time?)
- Impact of mode of delivery on patient engagement/disclosure
- What kind of **questions** have **participants asked** you during their assessment appointments? (Have you felt that you have had the appropriate knowledge/skills to address their questions?)
- **What outcomes would define a successful appointment** – for you and for the participant?

Sleep intervention set up and referral procedure

- **Experience of setting up the interventions** (e.g. clear what to do/what information needed) **& challenges** (e.g. free Headspace trial already used? how was this managed/what did they recommend)
- **Sleepstation referral process** (Any challenges, how long has it taken)
- **Information and support participants want** about **getting started with the sleep interventions**? (Able to give patients the support/info they needed? Paper versions of the documents requested?)

Follow-up phone call

- **How have you found** doing the **4-week follow-up phone calls**? (Challenges getting hold of patients, any [illegible]

Conclusion

- **Additional training or information needs**? (How/When/Why)
- Thoughts on how **training itself, manual** and **documents could be improved**? (i.e. changes needed)
- **Recommendations for refinements** needed to improve way in which the intervention is delivered? (What do you think has worked well? What hasn't worked so well?)
- How has being part of REST/your REST role **benefited you either personally or professionally**?

***Explore confidence around setting the SMART goals

***Do they feel they understand the theory behind the different interventions and are they able to communicate it to patients?

***Do they feel that they have a good understanding of what SleepStation involves for a patient and are they able to/do they communicate this to patients?

***Have they kept a reflective diary?

For peer review only

Appendix 42: Participant outcomes and health economic data

Comparison of SCI between patients eligible at screening and randomized trial participants

Characteristic	In trial, N = 57[1]	Not in trial, N = 201[1]	p-value[2]
**SCI_Score**	13 (8, 17)	17 (10, 26)	0.004
Unknown	0	49	
[1]Median (IQR)			
[2]Wilcoxon rank sum test			

Pain outcomes at 3 months postoperative (Oxford Knee Score)

Characteristic	Intervention, N = 6[1]	95% CI[2]	Control, N = 9[1]	95% CI[2]
**On-going pain (OKS<14)**				
No on-going pain	5 (83%)	36%, 99%	8 (100%)	60%, 100%
On-going pain	1 (17%)	0.88%, 64%	0 (0%)	0.00%, 40%
Unknown	0		1	
[1]n (%)				
[2]CI = Confidence Interval				

Neuropathic pain outcomes at 3 months postoperative

	**Baseline**		**Pre-operative**		**Post-operative**	
**Characteristic**	Intervention, N = 28[1]	Control, N = 29[1]	Intervention, N = 15[1]	Control, N = 25[1]	Intervention, N = 6[1]	Control, N = 9[1]
**PainDETECT score**	18 (11, 21)	13 (9, 19)	11 (10, 17)	14 (10, 24)	7 (6, 12)	10 (6, 16)
	4	4	6	5	2	3
**PainDETECT category**						
Ambiguous	6 (25%)	5 (20%)	3 (33%)	5 (25%)	0 (0%)	1 (17%)
Neuropathic likely	9 (38%)	8 (32%)	1 (11%)	7 (35%)	1 (25%)	1 (17%)
Nociceptive	9 (38%)	12 (48%)	5 (56%)	8 (40%)	3 (75%)	4 (67%)
Unknown	4	4	6	5	2	3
[1]Median (IQR); n (%)						

EQ-5D-5L and ICECAP scores

Measure	Baseline			Pre-operation			Post-operation		
	Intervention (SD)	Usual care (SD)	Response	Intervention (SD)	Usual care (SD)	Response	Intervention (SD)	Usual care (SD)	Response
EQ-5D-5L	**0.34** (0.30)	**0.47** (0.21)	57/57	**0.35** (0.22)	**0.40** (0.23)	40/57	**0.63** (0.24)	**0.69** (0.13)	14/27*
ICECAP	**0.71** (0.26)	**0.78** (0.14)	56/57	**0.71** (0.23)	**0.80** (0.14)	40/57	**0.83** (0.25)	**0.91** (0.021)	15/27*

*Only collected for those who had TKR within trial.

Intervention costs

Treatment Cost per person	Mean	Max	Min
Staff	£103.73	£214.50	£33.00
NHS Treatment cost§	30.72	£61.45	£12.29
Societal treatment cost¥	£6.59	£19.78	£0
NHS Total (per person)	£134.45	£275.95	£45.29
Societal Total (per person)	£141.04	£295.73	£45.29

§ Mean - 50% discount given to NHS, Max -no discount, Min - 80% discount offered to NHS.
¥ Mean -1/3 of participants paid for Headspace and Calm app subscriptions, Max all pay for subscriptions, Min- no one pays for subscription.
*https://www.nice.org.uk/process/pmg36/chapter/economic-evaluation

For peer review only

Appendix 53: Demographics of interviewed participants

Formatted Table

Pseudonym	Gender	Age at interview	Intervention or Usual Care	Mode of intervention delivery	Chosen sleep intervention
Florence	Woman	90	Intervention	Telephone	Relaxation
George	Man	64	Usual	N/A	N/A
Joyce	Woman	73	Usual	N/A	N/A
Gail	Woman	64	Usual	N/A	N/A
Charles	Man	79	Intervention	Video	CBT-I
Patricia	Woman	69	Intervention	Video	CBT-I
Gloria	Woman	73	Intervention	Video	Mindfulness
Ruth	Woman	72	Intervention	Video	CBT-I
Arthur	Man	72	Usual	N/A	N/A
Edward	Man	69	Intervention	Telephone	Mindfulness
Jerry	Man	64	Intervention	Video	Relaxation
Steven	Man	58	Usual	N/A	N/A
Rose	Woman	68	Intervention	Video	Mindfulness then CBT-I

For peer review only

Appendix 46: Qualitative themes and quotations

Theme	Subtheme	Quotations
**3. Explore engagement with the REST intervention and adherence to the agreed sleep plan**	Practitioner manner and communication style	*Well I think I was speaking to a professional and when I feel that, that gives me a little confidence in talking to her […] and I found that with [the practitioner] she was really very patient and in discussing her ideas […] she was very encouraging actually …[Florence]*<br>*It's been good. Just talk about things and get it into the open, don't bottle everything up [...] it suits me fine. [Jerry]*<br><br>*Well she was quite firm I felt in telling me about relaxing and how I would relax and she really wanted me to make up my mind then and there and tell her what I was planning to do and that made me feel more positive about it [...] she made me discuss it and having done that I was able to make my own mind up… she was suggesting breathing exercises as well […] there was no insistence at all from her, but on the other hand she was very firm in her suggestions [yes] which made me feel that I must do those things [Florence]*
**1. Evaluate intervention acceptability to patients and health professionals**	Patient acceptability: delivery mode	*I'm using Zoom with family etcetera, so I've used it for over a year now and I'm happy with it […] I'm confident using it [Patricia]*<br><br>*I'm not good on this Wi-Fi stuff, I'm really quite awful on it and my poor son was trying to train me […] I don't know how to cope or how to use anything with Wi-Fi, I find it very irksome, I really do, very overwhelmingly worrying, I just worry about it. [Florence]*<br><br>*I think this sort of appointment you don't need to be face-to-face. [...] There's no point doing face-to-face. Yes, it would be a 50-mile round trip for me to come to you […] It's fine seeing each other like this to be honest. [...] Travel time, parking, petrol. It's saving a fair bit of money from my point of view. [Patricia]*<br><br>*I don't see a disadvantage. Sitting in my own home rather than going to a hospital where I could pick up more diseases or COVID again. [Gloria]*
	Patient acceptability: appointment length and structure	*There was somebody there who could give me suggestions on the way whilst waiting for my operation, do you know what I mean? It's almost as though she was giving me something to do and think about as I approached the operation … [Florence]*

For peer review only

		The phone call took place not at the right time. But that was fine by me because I wasn't doing anything else. [Patricia]<br><br>*I'm not pleased, I've taken time off work to do this, but I was happy to take time off work to do it but not to be able to do it… [Ruth]*
	Practitioner acceptability: mode of delivery	*I'd be the first to admit that my IT is not as good as it should. I don't do much in the way of IT. […] I'm not the slickest at getting it all up on the computer. […] I find the IT thing a bit of a challenge sometimes [ESP03].*<br><br>*They've all been there ready and waiting and a few times, I've been late finishing the morning clinic because I normally do them on a Monday afternoon. They've all been ready and waiting at their phone or their computer. [ESP03]*
	Practitioner acceptability: intervention handbook and paperwork	*The form was very easy to follow for all the questions [ESP01]*<br><br>*The actual booklet that you're given yes, that's easy. I think maybe I probably need to introduce it a little bit better and what the study is, but then the actual questions are easy to follow, and then it follows onto the SMART goals and things, yes. [ESP01]*<br><br>*I think overall fine, I think sometimes you do feel like you're duplicating quite a lot I think, and when I was actually working through paperwork too quickly and not really sitting down and really going in depth about what the question's asking. Sometimes it feels like, well see above, kind of thing. Maybe that's because I'm classically the kind of person that will write everything down in the first box [RE 4-week call paperwork] [ESP04]*
**6. Evaluate the acceptability of randomisation**		*I understand that that's the only way you can gauge whether what you're doing is of any benefit if it's all randomised. You're not picking out a group of people that are better than another group of people in terms of their symptoms. [Joyce]*<br><br>*I would have particularly liked it if I was one of the people that was offered sort of help with sleep and so on, because the ideal for me would be to find an alternative to running through the highs and lows of my life at three o'clock in the morning would be good. So I was a bit disappointed. [Arthur]*
**9. Collect data on patient-reported outcomes measures to assess data**		*Things affect you differently at different times […] some days, if you're feeling really well, I think you fill it in through rose-tinted glasses. That's the only way I can describe it. If you were having a really good day*

For peer review only

**completion rates and inform the selection of the primary outcome measure and sample size for a full trial**		*and things were going well, you'd fill it in a little bit differently … [Joyce]*

For peer review only

For peer review only

Formatted: Indent: First line: 0 cm

For peer review only

BMJ Open

REST: A pre-operative tailored sleep intervention for patients undergoing total knee replacement: feasibility study for a randomised controlled trial

Journal:	*BMJ Open*
Manuscript ID	bmjopen-2023-078785.R2
Article Type:	Original research
Date Submitted by the Author:	08-Feb-2024

Complete List of Authors:	Bertram, Wendy; University of Bristol Medical School, Musculoskeletal Research Unit; NIHR Bristol Biomedical Research Centre<br>Penfold, Chris; University of Bristol Medical School, Musculoskeletal Research Unit<br>Glynn, Joel; University of Bristol Medical School<br>Johnson, Emma; University of Bristol Medical School<br>Burston, Amanda; University of Bristol Medical School, Musculoskeletal Research Unit<br>Rayment, Dane; North Bristol NHS Trust<br>Howells, Nicholas; North Bristol NHS Trust<br>White, Simon; Cardiff and Vale University Health Board<br>Wylde, Vikki; University of Bristol Medical School, Musculoskeletal Research Unit; NIHR Bristol Biomedical Research Centre<br>Gooberman-Hill, Rachael; University of Bristol Medical School; NIHR Bristol Biomedical Research Centre<br>Blom, Ashley; The University of Sheffield<br>Whale, Katie; University of Bristol Medical School, Musculoskeletal Research Unit; NIHR Bristol Biomedical Research Centre

**Primary Subject Heading**:	Health services research
Secondary Subject Heading:	Qualitative research, Surgery
Keywords:	Knee < ORTHOPAEDIC & TRAUMA SURGERY, SLEEP MEDICINE, Feasibility Studies

SCHOLARONE™
Manuscripts

I, the Submitting Author has the right to grant and does grant on behalf of all authors of the Work (as defined in the below author licence), an exclusive licence and/or a non-exclusive licence for contributions from authors who are: i) UK Crown employees; ii) where BMJ has agreed a CC-BY licence shall apply, and/or iii) in accordance with the terms applicable for US Federal Government officers or employees acting as part of their official duties; on a worldwide, perpetual, irrevocable, royalty-free basis to BMJ Publishing Group Ltd ("BMJ") its licensees and where the relevant Journal is co-owned by BMJ to the co-owners of the Journal, to publish the Work in this journal and any other BMJ products and to exploit all rights, as set out in our licence.

The Submitting Author accepts and understands that any supply made under these terms is made by BMJ to the Submitting Author unless you are acting as an employee on behalf of your employer or a postgraduate student of an affiliated institution which is paying any applicable article publishing charge ("APC") for Open Access articles. Where the Submitting Author wishes to make the Work available on an Open Access basis (and intends to pay the relevant APC), the terms of reuse of such Open Access shall be governed by a Creative Commons licence – details of these licences and which Creative Commons licence will apply to this Work are set out in our licence referred to above.

Other than as permitted in any relevant BMJ Author's Self Archiving Policies, I confirm this Work has not been accepted for publication elsewhere, is not being considered for publication elsewhere and does not duplicate material already published. I confirm all authors consent to publication of this Work and authorise the granting of this licence.

For peer review only

Title

REST: A pre-operative tailored sleep intervention for patients undergoing total knee replacement: feasibility study for a randomised controlled trial

Target Journal: BMJ Open BMJ Open submission guidelines

Authors

Wendy Bertram[1,2], Chris Penfold[1], Joel Glynn[1], Emma Johnson[1], Amanda Burston[1], Dane Rayment[3], Nick Howells[3], Simon White[4], Vikki Wylde[1,2], Rachael Gooberman-Hill[1,2], Ashley Blom[5], Katie Whale[1,2].

Corresponding Author: Wendy Bertram, Wendy.Bertram@bristol.ac.uk

Affiliations

1. University of Bristol
2. NIHR Bristol Biomedical Research Centre, University Hospitals Bristol and Weston NHS Foundation Trust and University of Bristol
3. North Bristol NHS Trust
4. Cardiff and Vale University Health Board
5. University of Sheffield

For peer review only

Abstract

Objectives

To test the feasibility of a randomised controlled trial of a novel pre-operative tailored sleep intervention for patients undergoing total knee replacement.

Design

Feasibility two-arm two-centre randomised controlled trial using 1:1 randomisation with an embedded qualitative study.

Setting

Two NHS secondary care hospitals in England and Wales.

Participants

Pre-operative adult patients identified from total knee replacement waiting lists with disturbed sleep, defined as a score of 0-28 on the Sleep Condition Indicator questionnaire.

Intervention

The REST intervention is a pre-operative tailored sleep assessment and behavioural intervention package delivered by an Extended Scope Practitioner (ESP), with a follow-up phone call four-weeks post-intervention. All participants received usual care as provided by the participating NHS hospitals.

Outcome measures

The primary aim was to assess the feasibility of conducting a full trial. Patient reported outcomes were assessed at baseline, one-week pre-surgery, and 3-months post-surgery. Data collected to determine feasibility included the number of eligible patients, recruitment rates, and intervention adherence. Qualitative work explored the acceptability of the study processes and intervention delivery through interviews with ESPs and patients.

Results

Screening packs were posted to 378 patients and 57 patients were randomised. Of those randomised, 20 had surgery within the study timelines. An appointment was attended by 25/28 (89%) of participants randomised to the

For peer review only - http://bmjopen.bmj.com/site/about/guidelines.xhtml

intervention. Follow-up outcomes measures were completed by 40/57 (70%) of participants pre-surgery and 15/57 (26%) post-surgery. Where outcome measures were completed, data completion rates were 80% or higher for outcomes at all timepoints, apart from the painDETECT: 86% complete at baseline, 72% at pre-surgery and 67% post-surgery. Interviews indicated that most participants found the study processes and intervention acceptable.

Conclusions

This feasibility study has demonstrated that with some amendments to processes and design, an RCT to evaluate the clinical and cost-effectiveness of the REST intervention is feasible.

Trial Registration: ISRCTN14233189

Article Summary: Strengths and limitations of this study

- COVID-19 restrictions in place during delivery required study procedures to be redesigned to enable remote data collection.
- Data collection at the 3-month post-surgery timepoint was limited due to the volume of operations performed during the study.
- This study was undertaken in two National Health Services hospitals, which demonstrates that it is feasible to undertake a full trial in these settings, however the findings may not necessarily be generalisable to other settings.
- Embedded qualitative work provided important insight into final study design to support acceptability and participant engagement with a full trial.

For peer review only

Background

Over 100,000 total knee replacements (TKR) are performed yearly in the UK.[1, 2] The primary reason for surgery is severe chronic pain and functional limitation due to end-stage osteoarthritis. The aim of TKR is long-term pain relief and improved function. Outcomes after knee replacement surgery are good, and surgical complications are rare, however approximately 20% of patients report dissatisfaction due to ongoing pain and functional limitations[3, 4].

Sleep issues are a substantial problem for people awaiting joint replacement; 60-75% of people with osteoarthritis and 70% of patients awaiting joint replacement experience sleep problems, which increase with condition severity.[5-8] Patients report issues with sleep onset and maintenance, and middle of the night waking.[9]

Poor sleep causes worsening joint pain, depressive symptoms, lower physical activity, and increased risk of cardiovascular and pulmonary disease in patients with osteoarthritis.[10, 11] Poor sleep can negatively impact surgical recovery causing slower wound healing, impaired immune function, increased risk of infection, and longer hospital stays.[12-15] Surgical patients with pre-operative sleep disturbances are at greater risk of developing post-operative delirium and surgical complications.[16] Sleep is bidirectionally linked with pain with poor sleep increasing pain sensitivity and inflammatory markers associated with pain[15, 17]. Poor sleep before TKR is associated with increased acute and chronic post-surgical pain, increased analgesic use, reduced joint function and range of motion, lower satisfaction, and longer inpatient stays.[8, 18, 19]

Previous trials on sleep and joint replacement have predominantly focused on peri- and post-operative pharmacological interventions.[20-22] A recent systematic review identified that improved pre-operative sleep reduced pain levels and analgesic consumption after TKR.[23] Guidance from the National Institute for Health and Care Excellence (NICE) and European Alliance of Associations for Rheumatology (EULAR) advises avoidance of pharmacological therapy for long-term management of sleep issues and recommends behavioural approaches as first-line treatment.[24, 25] Non-pharmacological sleep interventions are potentially more sustainable and cost-effective, with lower risk of side-effects.[26] Our recent systematic review identified no RCTs evaluating a non-pharmacological intervention targeting sleep in patients waiting for TKR.[27]

This study aimed to evaluate the feasibility of conducting an RCT to evaluate the clinical and cost-effectiveness of REST, a non-pharmacological complex sleep intervention for patients undergoing TKR.

Methods

Study design

REST is a two-centred randomised controlled feasibility trial with 1:1 randomisation and an embedded qualitative study. Participants were recruited from two secondary care NHS hospitals in England and Wales. Ethics approval was obtained from the South West Cornwall & Plymouth Research Ethics Committee (20/SW/0189). The trial was prospectively registered (ISRCTN14233189). The CONSORT checklist is provided in appendix 1.

Patient and public involvement (PPI)

For peer review only - http://bmjopen.bmj.com/site/about/guidelines.xhtml

The study was developed in collaboration with a musculoskeletal PPI group working in partnership with the University of Bristol and North Bristol NHS Trust. The study benefited from the active involvement and contributions from a group of experienced patient partners. The group comprised five patients with lived experience of knee replacement. They met four times to co-design patient-facing study materials, monitor study progress, provide input into study process, and review the study results and dissemination plans. A patient partner was a member of the Steering committee.

Participant identification, recruitment and randomisation

Patients waiting for a primary TKR for osteoarthritis were identified from surgical waiting lists. Those likely to have surgery within three months were sent a pre-notification card by post followed by a screening questionnaire. The three-month preoperative timepoint was selected to allow sufficient time for intervention delivery and engagement to affect behaviour change.

Eligibility criteria were: adults on the TKR waiting list, experiencing disturbed sleep (defined as a score of 0-28 on the Sleep Condition Indicator (SCI) questionnaire, a validated screening tool for insomnia[28]), and access to a device with internet connection. Exclusion criteria were: diagnosed with or receiving treatment for a clinical sleep disorder, taking prescription medication to help with sleep, having taken part in an interventional sleep study in the past six months, and unable or unwilling to attend an intervention appointment, provide informed consent or complete questionnaires in English. Patients who returned an eligible screening questionnaire were telephoned for an eligibility assessment. Eligible patients were invited to a recruitment visit. Patients not eligible at screening were sent a thank you letter.

After written informed consent was provided and the baseline questionnaire completed, participants were randomly allocated to the intervention plus usual care or usual care alone. Randomisation was conducted on a 1:1 intervention:control basis by the co-ordinating centre using computer generated randomisation. Participants, practitioners, and research staff were not blinded.

Intervention

The REST intervention was developed following Medical Research Council guidance for complex intervention development.[29, 30] The TIDieR checklist is provided in the appendix 2. REST consists of an appointment with an Extended Scope Practitioner (ESP) delivered via videoconference or telephone three months before surgery. The one-hour appointment comprised a comprehensive sleep assessment to identify individual sleep issues and needs, and an assessment of sleep apnoea risk. Participants scoring high risk for sleep apnoea were referred to their GP in addition to the intervention. Participants were then provided with tailored sleep education and sleep hygiene advice. One of three existing evidence-based sleep interventions (ESIs) was recommended through a shared decision-making process: cognitive-behavioural therapy for insomnia (delivered via online platform Sleepstation), relaxation (delivered via the Calm app, workbook, or guided audio/video), and mindfulness (delivered via the Headspace app, workbook, or guided audio/video).

Participants were provided with a personalised sleep plan, which included SMART goals based on the sleep hygiene recommendations (for example reducing coffee intake, removing electronics from the bedroom, starting a bedtime routine / sleep schedule), a detailed overview of their chosen ESI, instructions for use and digital access (if applicable) and any materials. Participants received a follow-up telephone call four weeks after the appointment to

For peer review only - http://bmjopen.bmj.com/site/about/guidelines.xhtml

review progress and engagement with their sleep plan, calls lasted approximately 30-45 minutes. This included addressing any barriers experienced, review of the sleep goals, and adjustments to the sleep plan if needed.

All participants received usual care as provided by the participating NHS hospitals. Safety reporting was exclusively for adverse reactions directly attributable to the intervention.

Intervention delivery training

All practitioners took part in a one-day online intervention delivery training session. This covered the study background, evidence on the relationship between sleep and pain, an overview of each ESI, and practical guidance on delivery. The Chief Investigator communicated regularly with practitioners to provide further support and training if required. ESPs were provided with a detailed intervention manual, which provided guidance and proformas for conducting the sleep assessment, sleep hygiene and education advice, information on each ESI, participant sleep plan, and post-appointment tasks.

Intervention timing

REST was designed to be delivered 3-months pre-surgery. This timepoint was chosen to optimise the effect of the sleep interventions, because of the duration of the sleep interventions (Sleepstation is delivered over 6-8 weeks), and theories of behaviour change maintenance[31].

Intervention delivery fidelity

Non-participatory observations were conducted to assess the degree to which the intervention was delivered as intended as per the intervention manual. One clinic appointment and follow-up call were observed for each ESP. Observations were conducted independently by two members of the research team. Participants were asked to provide verbal consent for the researcher to be present during their clinic appointment.

Feasibility outcomes

Feasibility outcomes included recruitment rate, intervention uptake and adherence, outcome data completion, and intervention acceptability[32]. A full list of outcomes and measurements are outlined in Table 1.

Table 1: Feasibility outcomes

Objective(s)	Outcome	Measurement
1	Eligibility and recruitment rates	Number of patients invited, returning screening questionnaires, eligible, consented and randomised. Retention rates.
2	Intervention uptake	Number of participants who attend the clinic appointment
3	Intervention adherence	Open ended questions at 4-week follow-up telephone call:<br>• Changes made as a result of sleep hygiene and education advice<br>• Engagement in the assigned sleep intervention<br>• Any additional changes made to sleep or sleep routine
4 & 6	Participant interviews: acceptability of the	Semi-structured qualitative interviews with participants in the intervention group (n=20, 10 per site), and the control group (n=5).

For peer review only - http://bmjopen.bmj.com/site/about/guidelines.xhtml

	intervention and randomisation	
4 & 8	Extended scope practitioner interviews: acceptability of the intervention and training optimisation	Semi-structured qualitative interviews with ESPs (n=4) at two timepoints: 1) after completion of training and 2) after intervention delivery
5	Intervention delivery fidelity	Observation of one clinic appointment and follow-up call for each ESP to assess adherence and compliance.
7	Health economics data	Quality of life measures (EQ-5D-5L, ICECAP-A) and health care resource use (community and secondary care) as documented in the patient completed outcome measure booklets at one week pre-operative and three months post-operative.
8	Optimisation of intervention training	Non-participatory observations of the ESP training.<br>Semi-structured interviews with all ESPs (n=4) at two time points, after completion of training and after delivering the intervention.
9	Inform the primary outcome measure for a full trial	Quantitative data analysis, proportion of participants in ongoing pain in each treatment arm at three months after surgery.

For peer review only

Patient-reported outcomes

Patient-reported outcomes were assessed using paper questionnaires prior to randomisation (approximately 3 months pre-operative), one week prior to surgery and three months after surgery. Participants who did not have their operation by six-months post-randomisation completed pre-surgery outcomes.

Outcomes included joint pain (Oxford Knee Score (OKS)[33]), neuropathic pain (painDETECT[34]), sleep quality and beliefs about sleep (Sleep Condition Indicator (SCI[28]), Pittsburgh Sleep Quality Index (PSQI)[35]), mental wellbeing (Hospital Anxiety and Depression Scale (HADS)[36]), and general health and wellbeing (EQ-5D-5L[37],ICECAP-A[38]). Health resource use data included health care interactions in the community and secondary care, including medication use, and were collected in the three-month pre-operative and post-operative questionnaires only. Intervention participants completed sleep treatment engagement questions at the 3-month post-surgery timepoint.

Qualitative study

Embedded qualitative work explored the acceptability of the intervention and study processes. Interviews were conducted with participants in both arms and with ESPs delivering the intervention. Participants who expressed an interest at enrolment in being interviewed were sent an invitation letter, reply slip, and pre-paid envelope. Participants who returned the reply slip were contacted a researcher to discuss participation and arrange an interview for those interested. Informed consent was provided by participants before interview. ESPs were invited to participate in two interviews: one following the intervention training day and one after delivery of intervention appointments. Informed written or recorded verbal consent was provided by all ESPs.

Qualitative data collection

All interviews were conducted via videoconference or telephone depending on preference. Face-to-face interviews were not possible due to COVID-19 restrictions. Participant interviews were guided by semi-structured topic guides (Appendix 3) covering design and conduct of the trial (all participants), experiences of the intervention and views on impact (intervention group), and changes to made to sleep (all participants). ESP interviews at both timepoints explored acceptability of training and intervention delivery.

Progression criteria

Progression criteria for demonstrating the feasibility of a RCT were proposed as ≥60 patients randomised (75% of target) and 75% uptake of the intervention. Uptake was defined as the number of participants who attended an intervention appointment. Criteria for progression based on acceptability were:

- All participants: expressed comfort with study processes including recruitment, randomisation, outcome measures and follow-up.
- Intervention participants: level of engagement with clinic processes, adherence to and engagement with the intervention.

Sample size

The target sample was 80 participants (40 intervention, 40 usual care) to estimate 75% randomisation rate (RCT progression criteria) with 95% confidence interval from 65% to 85%, and to estimate 75% intervention uptake with 95% confidence interval from 60% to 90%.

Statistical analyses

For peer review only - http://bmjopen.bmj.com/site/about/guidelines.xhtml

Baseline characteristics of each group were tabulated using means and standard deviations for normally distributed data, medians and interquartile ranges for non-normally distributed data, and percentages and counts for categorical data. Patient-reported outcome measures were summarised descriptively. The proportion of people without complete responses for the outcome questionnaires were reported at each timepoint with commentary on any patterns of missing data between timepoints. Outcome data tables are provided in appendix 4.

Economic analyses

The feasibility of collecting data for an economic evaluation alongside a full trial was assessed, including intervention costs for appointments and tailored intervention. Economic analysis tables are provided in appendix 4.

Qualitative analysis

Data collection and analysis were conducted in parallel after the first three interviews. Audio files were transcribed, then transcripts were anonymised and imported into the qualitative software package NVivo10. Participant and ESP data were analysed separately using Framework Analysis, a thematic approach that enables structured comparison and contrast of data across cases.[39] Data were organised using the topic guide as a starting framework. Five transcripts were independently double coded and discussed within the team to offer further insight into interpretation and to enhance rigour through different approaches and knowledge.[40] All participants were assigned pseudonyms to ensure anonymity. Participant demographics and supporting quotes are included in appendices 5 and 6.

Results

1. Eligibility and recruitment rates

Between March and December 2021, 378 patients were invited to take part in screening. Of these, 258 (68%) returned completed screening questionnaires: 146 were willing to take part and met screening eligibility criteria, 58 declined to take part and 54 were not eligible. Reasons for ineligibility included: SCI score ≥29 (28/54, 52%), having a sleep disorder or taking medication to help with sleep (18/54, 33%), operation dates allocated in the near future or surgery being postponed (4/54, 7%), questionnaire returned after study closure (3/54, 6%), and no internet access (1/54, 2%).

Telephone calls were made to the 146 patients who returned an eligible screening questionnaire: 8 were not contactable, 32 were ineligible, 47 declined to take part and 59 consented to take part. Reasons for ineligibility at telephone screening included operation dates allocated in the near future or surgery being postponed (13/32, 41%), having a sleep disorder or taking medication to help with sleep (9/32, 28%), no internet access (7/32, 22%), already had surgery (2/32, 6%), and did not speak English (1/32, 3%). Most did not give a reason for declining taking part. Where given, common reasons for declining were time commitments or personal circumstances (17/47, 36%) and did not feel they had a sleep problem or did not think treatment would help (6/47, 13%).

Two participants withdrew prior to randomisation; therefore 57 participants were randomised.

A CONSORT diagram outlining participant flow is provided in Figure 1. Baseline participant characteristics are provided in Table 2. Patients who were eligible but did not participate had higher (better) preoperative sleep as measured using the SCI than randomised participants (appendices).

For peer review only - http://bmjopen.bmj.com/site/about/guidelines.xhtml

Table 2: Characteristics of study participants at baseline

Characteristic	N = 57[1]
**Gender**	
Man	23 (40%)
Woman	34 (60%)
**Ethnicity (cleaned)**	
Non white/non British	1 (1.8%)
Other/not answered	4 (7.0%)
White/British	52 (91%)
**Do you consume any alcohol?**	
No	26 (46%)
Yes	30 (54%)
Unknown	1
**Alcohol units/week (excl. non-drinkers)**	9 (3, 14)
Non-drinker/unknown	27
**Do you consume any coffee?**	
No	16 (28%)
Yes	41 (72%)
**Cups of coffee/week (excl. non-drinkers)**	12 (7, 20)
Non-drinker/unknown	16
**Smoking status**	
Current	2 (3.5%)
Former	24 (42%)
Never	31 (54%)
**Employment status**	
Employed	11 (19%)
Other	2 (3.5%)
Retired	44 (77%)
**Marital Status**	
Divorced	9 (16%)

For peer review only - http://bmjopen.bmj.com/site/about/guidelines.xhtml

Characteristic	N = 57[1]
Married/partner	36 (63%)
Single	2 (3.5%)
Widowed	10 (18%)
**Other conditions**	
No	18 (34%)
Yes (please state)	35 (66%)
Unknown	4
**Other condition(s): Other joint replacement**	
Yes	4 (100%)
Unknown	53
**Other condition(s): Pain in other joints**	
Yes	4 (100%)
Unknown	53
**Other condition(s): Arthritis (any)**	
Yes	29 (100%)
Unknown	28
**Other condition(s): Injury**	
Yes	3 (100%)
Unknown	54

[1]n (%)

2. Attendance at the intervention clinic appointment

Of the 28 participants assigned to the intervention group, 3 withdrew before receiving the intervention due to operation dates being allocated in the near future (10.7%), 25 attended the clinic appointment (89.3%), and 15 completed the 4-week follow-up call (53.6%).

3. Engagement with the REST intervention and adherence to the agreed sleep plan

Qualitative study: participant interviews

38 trial participants were invited to take part in an interview (N=18 Bristol/20 Cardiff; 22 intervention, 16 usual care). Sixteen expressions of interest were received, and 13 participants were interviewed (N= 10 Bristol/3 Cardiff; N= 8 intervention/5 usual care. Ethnicity was reported as White British (n=7), English (3), Welsh (n=2), and White (n=1).

For peer review only - http://bmjopen.bmj.com/site/about/guidelines.xhtml

Marital status was reported as married/partner (n=8), widowed (n=2), single (n=2), and divorced (n=1). Further participant characteristics are described in Appendix 5.

Participants were willing to discuss their sleep issues with the ESPs. Factors influencing readiness to engage in a conversation with the practitioner included a belief that they were meeting with a knowledgeable and skilled professional. The practitioner's manner and communication style helped to create a safe space that enabled participants to feel both at ease and comfortable to open up about their experiences. Participants who recalled the shared decision-making process of choosing a sleep intervention said that they felt involved and informed.

4. Intervention acceptability

Patient acceptability

COVID-19 restrictions at the time of delivery required all recruitment and intervention appointments to be conducted remotely by videoconference or telephone. Participants understood the need for remote appointments, but confidence and familiarity with this approach varied. For many participants confidence in using video calls had grown during the pandemic. One participant struggled with confidence and familiarity with online appointments and needed additional support. Remote delivery was generally seen as acceptable. Participants highlighted benefits of removed travel and cost, and reduced risk of COVID-19 and other infections.

Appointment length and structure were considered appropriate. Participants felt they had enough time to ask questions, discuss what was being asked of them, and to address concerns. The time to attend the appointment was seen as worthwhile, as it gave access a practitioner who provided the chance to talk about their problems and a focus during their wait for surgery.

Due to clinic delays and competing demands of the practitioners, some appointments started later than planned. Although some participants were accepting of this, one participant reported feeling frustrated and angry at the inconvenience caused.

No adverse events were reported.

Practitioner acceptability

ESP acceptability of intervention delivery was high. ESPs were able to deliver most appointments using videoconference which supported better communication with participants, however one ESP expressed a preference for telephone appointments as this required less set up and had greater flexibility. Some technical issues were raised due to ESPs using different NHS computers for appointments depending on their schedule. This caused problems with webcam connectivity and added additional time to appointment set up. Overall paperwork was straightforward to complete with the questions and proformas clear and easy to use. Some aspects of the assessment were viewed as repetitive and could be shortened to give more time for discussion with participants.

5. Intervention delivery

Intervention fidelity assessments included observation of at least one clinic appointment and one follow-up appointment for each practitioner. Practitioners fully or partially met all areas of adherence (fidelity to the intervention as described in the manual) and compliance (proficiency of delivery) during the intervention

For peer review only - http://bmjopen.bmj.com/site/about/guidelines.xhtml

appointment. Three areas for improvement in training and delivery were identified: educating the participant about sleep and TKR, setting SMART sleep goals, and shared-decision-making discussions around recommended sleep interventions.

6. Acceptability of randomisation

Qualitative interviews with participants found that most participants found randomisation acceptable and understood the need for this design. Some expressed disappointment on receiving usual care. Disappointment stemmed from their desire to benefit from the intervention because of struggles with sleep.

7. Feasibility and acceptability of collecting health economic data

Quality of life measures

EQ-5D-5L and ICECAP mean scores and standard deviations with response rates at each of the time point are presented in the appendices. EQ-5D-5L utility scores were calculated using Hernández Alava et al. 2020 method as recommended by NICE PMG36.[41] No evidence was found of ceiling of floor effects for either quality of life measure, and the measures seem to be responsive to quality of life changes in this population.

Resource use

Responses to the bespoke resource use questionnaire were 40/57 (70%) pre-operatively and 14/27 (52%) post-operatively. Changes and clarification of questions will be made based on responses to individual questions. For example, more options are needed for physiotherapy appointments as many responses were selected “other”. Most resource use and cost fell on NHS services including GP, outpatient, and physiotherapy appointments. Overall, the estimated cost (NHS perspective, excluding intervention costs) was £507 and £688 for intervention and usual care arms respectively. However, no interpretation of this difference can be made given the small number of participants in this feasibility study.

Intervention costs

Given we do not know the value or mechanism of payments from NHS to Sleepstation for use of the app, nor the number of patients purchasing app subscriptions to Calm and Headspace, we costed the intervention based on several assumptions (appendices). We have generated a minimum, mean, and maximum expected intervention cost. The mean cost of the intervention was estimated at £134.45 per person (£141.04 including patient out of pocket costs) based on the mean clinic, preparation, and post-clinic time captured in the feasibility study, an NHS cost of £147 per patient for the use of Sleepstation (based on a 50% discount on publicly advertised cost), and 1/3 of those using Calm or Headspace upgrading to paid subscriptions for additional access. The max estimated at £295.73 and minimum cost £45.29.

8. Optimisation of the intervention delivery training package

Four ESPs took part in an interview at timepoint one (post-training) and three at time-point two (post-delivery).

Training was delivered as a one-day online course. Practitioners spoke positively about their experience of the training, praising the organisation and focus. Practitioners appreciated receiving information about the rationale behind the intervention. Some felt the level of detail around sleep science could be reduced, but still found this interesting. They were given sufficient time to ask questions, however the remote format made this slightly harder

For peer review only - http://bmjopen.bmj.com/site/about/guidelines.xhtml

compared to face-to-face. Following experience of intervention delivery, ESPs reported challenges in setting SMART goals including what areas to focus on and the level of detail needed. They suggested more training and knowledge of each ESI would be beneficial and facilitate better shared decision-making discussions with participants. A small number of participants attending appointments felt they did not have a sleep issue. This made it challenging for ESPs to follow the intervention handbook.

Areas for improving practitioner training were identified as:

- Increased time for role-play and practical exercises
- Additional information on sleep interventions to increase understanding and familiarity
- Further training and practical exercises on setting SMART goals
- Advice and guidance on how to support participants who do not believe they have a sleep issue or are not motivated to make changes
- Additional supervision meetings throughout intervention delivery to provide further support, answer questions, and address challenges

9. Data completion rates, selection of the primary outcome measure and sample size for a full trial

Data completion rates

Data completion rates are provided in Table 3. All participants completed the baseline OKS and EQ5D-5L. Completion rates were consistently lower for the PSQI (86%-93%) at baseline and pre-operative timepoints. The painDETECT questionnaire had the lowest completion rates at each timepoint, ranging from 60-86%.

Table 3: Data completion rates

	Baseline		Pre-operative		Post-operative	
Characteristic	Intervention, N = 28[1]	Control, N = 29[1]	Intervention, N = 15[1]	Control, N = 25[1]	Intervention, N = 6[1]	Control, N = 9[1]
**OKS**						
Complete	28 (100%)	29 (100%)	13 (87%)	25 (100%)	6 (100%)	8 (89%)
Missing			2 (13%)	0 (0%)	0 (0%)	1 (11%)
**PainDETECT**						
Complete	24 (86%)	25 (86%)	9 (60%)	20 (80%)	4 (67%)	6 (67%)
Missing	4 (14%)	4 (14%)	6 (40%)	5 (20%)	2 (33%)	3 (33%)
**SCI**						
Complete	27 (96%)	27 (93%)	15 (100%)	24 (96%)	6 (100%)	9 (100%)
Missing	1 (3.6%)	2 (6.9%)	0 (0%)	1 (4.0%)		
**HADS**						
Complete	27 (96%)	29 (100%)	12 (80%)	25 (100%)	6 (100%)	9 (100%)

For peer review only - http://bmjopen.bmj.com/site/about/guidelines.xhtml

Missing	1 (3.6%)	0 (0%)	3 (20%)	0 (0%)		
**PSQI**						
Complete	24 (86%)	27 (93%)	13 (87%)	21 (84%)	6 (100%)	9 (100%)
Missing	4 (14%)	2 (6.9%)	2 (13%)	4 (16%)		
**EQ5D-5L**						
Complete	28 (100%)	29 (100%)	15 (100%)	25 (100%)	6 (100%)	8 (89%)
Missing					0 (0%)	1 (11%)
**ICECAP**						
Complete	25 (89%)	29 (100%)	15 (100%)	25 (100%)	6 (100%)	9 (100%)
Missing	3 (11%)	0 (0%)				
[1]n (%)						

Qualitative interviews demonstrated that questionnaire completion was acceptable overall.

Patient-reported outcome measures

Baseline, pre-operative, and post-operative outcome measures are presented Table 4. The purpose of this study was to evaluate the feasibility of conducting an RCT, therefore statistical tests to compare outcomes between treatment arms were not performed.

For peer review only

For peer review only - http://bmjopen.bmj.com/site/about/guidelines.xhtml

Table 4: Patient reported outcome measures

	Baseline		Pre-operative		Post-operative	
	Intervention, N = 28[1]	Usual Care, N = 29[1]	Intervention, N = 15[1]	Usual Care, N = 25[1]	Intervention, N = 6[1]	Usual Care, N = 9[1]
**OKS pain subscale**	8.5 (6.0, 10.0)	9.0 (6.0, 11.0)	7.0 (6.0, 10.0)	8.0 (5.0, 12.0)	13.50 (11.50, 15.50)	14.50 (12.50, 16.00)
**OKS function subscale**	7.5 (3.8, 12.0)	9.0 (7.0, 12.0)	7.0 (5.0, 9.8)	8.0 (5.0, 11.0)	21.5 (19.5, 22.8)	22.5 (21.0, 24.0)
**OKS total score**	16 (12, 21)	18 (13, 22)	15 (11, 19)	16 (9, 22)	34 (32, 40)	36 (35, 39)
**PainDetect score**	18 (11, 21)	13 (9, 19)	11 (10, 17)	14 (10, 24)	7 (6, 12)	10 (6, 16)
**PainDetect score(categorised)**						
Ambiguous	6 (25%)	5 (20%)	3 (33%)	5 (25%)	0 (0%)	1 (17%)
Neuropathic likely	9 (38%)	8 (32%)	1 (11%)	7 (35%)	1 (25%)	1 (17%)
Nociceptive	9 (38%)	12 (48%)	5 (56%)	8 (40%)	3 (75%)	4 (67%)
**Sleep conditions indicator**	11 (8, 14)	13 (10, 16)	14.0 (11.0, 21.0)	15.0 (9.5, 18.0)	22 (16, 24)	24 (18, 27)
**HADS score**	13 (10, 22)	16 (12, 19)	12.5 (9.0, 19.0)	14.0 (9.0, 20.0)	9.0 (4.2, 13.0)	8.0 (6.0, 14.0)
**HADS score (categorised)**						
Abnormal	19 (70%)	22 (76%)	7 (58%)	17 (68%)	3 (50%)	3 (33%)
Borderline abnormal	5 (19%)	5 (17%)	3 (25%)	6 (24%)	0 (0%)	2 (22%)
Normal	3 (11%)	2 (6.9%)	2 (17%)	2 (8.0%)	3 (50%)	4 (44%)
**ICECAP**	0.85 (0.66, 0.92)	0.84 (0.70, 0.89)	0.84 (0.55, 0.90)	0.84 (0.67, 0.91)	0.92 (0.87, 0.98)	0.91 (0.89, 0.92)
**EQ-5D**	0.33 (0.16, 0.57)	0.54 (0.30, 0.70)	0.39 (0.22, 0.54)	0.45 (0.22, 0.60)	0.67 (0.60, 0.73)	0.76 (0.73, 0.77)
**PSQI global score**	12.0 (8.8, 14.2)	11.0 (8.0, 13.5)	8.0 (6.0, 11.0)	11.0 (7.0, 13.0)	9.5 (5.2, 11.5)	7.0 (5.0, 9.0)

[1]Median (IQR); n (%)

Participants randomised to the intervention group reported an improvement in average Pittsburgh Sleep Quality Index score from 12.0 (95% CI 8.8, 14.2) at baseline to 8.0 (95% CI 6, 11) at the end of the intervention (12 weeks after randomisation), compared to no change in the usual care group (baseline score of 11 (95% CI 8, 13.5) and 12-week score of 11 (95% CI 7, 13)).

A proposed primary outcome was pain after surgery as measured by the OKS pain component[42]. The target timing for randomisation was three months pre-operative. The mean number of days from randomisation to operation was 118 days, with 35% (n=20) of participants having surgery during the six-month participation window. Outside of the participation window, a further 18 participants were allocated an operation date. The remaining 21 had not been allocated a surgery date at study closure.

Discussion

This feasibility study has demonstrated that the REST intervention is acceptable to patients and clinicians. With modifications, a full trial is feasible. Criteria for progression to a full trial are ≥60 patients randomised (75% of target) and 75% uptake of the intervention. More than 75% of participants allocated the intervention attended the clinic appointment (89%, n = 25/29). We randomised 57 patients during a period of COVID-19 restrictions when many studies were unable to recruit. In addition, removing the need to screen patients who are three months prior to surgery would facilitate increased recruitment.

Strengths

Despite COVID-19 restrictions at the time of the study, screening and recruitment procedures were successful and 57 participants were randomised. Close working with waiting list staff and surgeons was essential to understanding which patients were most likely to be allocated a surgery date in three months. Once identified, the return rate for screening questionnaires was 68%. Evidence-based methods to increase the return of postal questionnaires were used, including pre-notification cards and non-monetary incentives (individually wrapped tea bags).

Most participants randomised to the intervention group attended an appointment (89%, n = 25/29) and engaged with treatment. Remote delivery of the intervention was viewed positively by participants. Those who had a video call who appreciated being able to see the practitioner, welcoming the human connection and chance to build rapport.

Limitations

Although intervention uptake and engagement with treatment was good, inequalities in access to the internet and electronic devices are an issue in studies that use remote delivery. Some participants experienced delays in obtaining appointment times, which varied by practitioner and site; this may be solved by centralised intervention delivery and offering options such as telephone delivery for those without internet access.

Patients who were eligible at screening but who chose not to take part in the study had better preoperative sleep as measured using the SCI than randomised participants. A common reason for not taking part was not having a sleep

For peer review only - http://bmjopen.bmj.com/site/about/guidelines.xhtml

For peer review only

problem or feeling that treatment would not help. This indicates the SCI eligibility score cutoff would benefit from being lowered.

Study delivery was redesigned to be conducted entirely remotely to meet COVID-19 restrictions, which also influenced the volume of knee replacement operations performed, affecting the number of participants undergoing surgery within the study.

Generally, NHS operation dates cannot be reliably predicted three months in advance, therefore identifying patients at this timepoint proved challenging. Completion of the primary outcome at three months post-surgery was also difficult because many participants did not have their operation within the study timelines. In addition, variations in length of time from randomisation to three months post-surgery would result in high heterogeneity. To address this, the primary outcome assessment for a full trial should not be the proposed outcome of pain three months post-operative, but sleep quality at 14 weeks post-randomisation timepoint for generalisability.

Modifications

There are several key areas to adapt and improve for a future full trial. These include changes to the clinician training programme, including more detailed training on existing sleep interventions and setting SMART goals, streamlined delivery of the intervention by provision of an online portal, and lowering to the screening cut-off for the SCI score.

A review of equality, diversity, and inclusion strategies will ensure a full trial supports inclusivity and engagement from a wide range of communities.

Conclusions

We have demonstrated that a full RCT is feasible based on the predefined progression criteria and have identified areas for improvement to optimise trial design. Recruitment is achievable, engagement with and adherence to the intervention is high and, importantly, the intervention is acceptable to patients and clinicians.

For peer review only

For peer review only - http://bmjopen.bmj.com/site/about/guidelines.xhtml

List of figures and tables

Figure 1: CONSORT participant flow diagram

Table 1: Feasibility outcomes

Table 2: Characteristics of study participants at baseline

Table 3: Data completion rates

Table 4: Patient reported outcome measures

Appendices

Appendix 1: CONSORT 2010 checklist of information to include when reporting a pilot or feasibility randomised trial in a journal

Appendix 2: The TIDieR (Template for Intervention Description and Replication) Checklist

Appendix 3A: Patient interview topic guide

Appendix 3B: ESP intervention training interview topic guide

Appendix 3C: ESP intervention delivery interview topic guide

Appendix 4: Participant outcomes and health economic data

Appendix 5: Demographics of interviewed participants

Appendix 6: Qualitative themes and quotations

Footnotes

Funding

This study is funded by the National Institute for Health and Care Research (NIHR) (Research for Patient Benefit; grant reference number NIHR201036). The research team acknowledges the support of the NIHR, through the Clinical Research Network. This work was also supported by the NIHR Biomedical Research Centre at University Hospitals Bristol and Weston NHS Foundation Trust and the University of Bristol. The views expressed are those of the authors and not necessarily those of the NIHR or the Department of Health and Social Care.

Competing interests: None declared.

Author contributions

KW was Chief Investigator. KW, RGH, VW, CP, AB, AWB, DR, NH, SW and JG were co-applicants on the grant application to NIHR. All authors were involved in the design, delivery and interpretation of the study. WB was the Trial Manager. JG was responsible for the economic analysis. CP was responsible for the statistical analysis. EJ and KW were responsible for the qualitative analysis. WB, KW, CP and JG have access and/or verified the underlying data. WB drafted the manuscript; all authors revised if for important content and approve the final manuscript.

For peer review only

Data sharing

Participants were asked on the consent form if they were willing for their information to be shared anonymously with other researchers to support other research in the future. Anonymised data will be stored on the University of Bristol Research Data Storage Facility (https://data.bris.ac.uk) and will be shared via the University of Bristol Research Data Repository within six months of the publication of the study results. Access to the data will be restricted to ensure that data is only made available to bona fide researchers after a Data Access Agreement has been signed by an institutional signatory.

Ethics

Patient consent for publication: Not applicable

Ethics approval

This study received a favourable opinion from the South West – Cornwall & Plymouth Research Ethics Committee, reference 20/SW/0189, and approval from the Health Research Authority and Health and Care Research Wales (IRAS 289761). Participants gave informed consent to participate in the study before taking part.

Acknowledgements

We would like to thank our patient partners for their help and input, and the research team members and health care professionals who delivered the study including: Gemma Munkenbeck, Leigh Morrison, Paolo Buscemi, Christine Hobson, Giles Head, Lucy Young, Jessica Falatoori, Mat Williams, Chris Dobson and Mark Brand.

The research team acknowledges the support of the NIHR, through the Clinical Research Network. This work was also supported by the NIHR Biomedical Research Centre at University Hospitals Bristol and Weston NHS Foundation Trust and the University of Bristol. The views expressed are those of the authors and not necessarily those of the NIHR or the Department of Health and Social Care.

For peer review only - http://bmjopen.bmj.com/site/about/guidelines.xhtml

References

1. Registry, N.J., *National Joint Registry 15th Annual Report 2018.*
2. Project, S.A., *Annual Report 2018*. 2018, NHS National Services Scotland.
3. Gunaratne, R., et al., *Patient Dissatisfaction Following Total Knee Arthroplasty: A Systematic Review of the Literature.* J Arthroplasty, 2017. **32**(12): p. 3854-3860.
4. Beswick, A.D., et al., *What proportion of patients report long-term pain after total hip or knee replacement for osteoarthritis? A systematic review of prospective studies in unselected patients.* BMJ Open, 2012. **2**(1): p. e000435.
5. Hawker, G.A., et al., *The multidimensionality of sleep quality and its relationship to fatigue in older adults with painful osteoarthritis.* Osteoarthritis Cartilage, 2010. **18**(11): p. 1365-71.
6. Wilcox, S., et al., *Factors related to sleep disturbance in older adults experiencing knee pain or knee pain with radiographic evidence of knee osteoarthritis.* J Am Geriatr Soc, 2000. **48**(10): p. 1241-51.
7. Manning, B.T., et al., *Prospective Assessment of Sleep Quality Before and After Primary Total Joint Replacement.* Orthopedics, 2017. **40**(4): p. e636-e640.
8. Bjurstrom, M.F., et al., *Preoperative sleep quality and adverse pain outcomes after total hip arthroplasty.* Eur J Pain, 2021. **25**(7): p. 1482-1492.
9. Smith, T.O., J.R. Dainty, and A.J. MacGregor, *Changes in social isolation and loneliness following total hip and knee arthroplasty: longitudinal analysis of the English Longitudinal Study of Ageing (ELSA) cohort.* Osteoarthritis Cartilage, 2017. **25**(9): p. 1414-1419.
10. Dai, Z., et al., *Sleep Quality Is Related to Worsening Knee Pain in Those with Widespread Pain: The Multicenter Osteoarthritis Study.* J Rheumatol, 2020. **47**(7): p. 1019-1025.
11. Song, J., et al., *Sleep Disturbance Trajectories in Osteoarthritis.* J Clin Rheumatol, 2021. **27**(8): p. e440-e445.
12. Kjolhede, P., et al., *The impact of quality of sleep on recovery from fast-track abdominal hysterectomy.* J Clin Sleep Med, 2012. **8**(4): p. 395-402.
13. Kamdar, B.B., D.M. Needham, and N.A. Collop, *Sleep deprivation in critical illness: its role in physical and psychological recovery.* J Intensive Care Med, 2012. **27**(2): p. 97-111.
14. Hoyle, N.P., et al., *Circadian actin dynamics drive rhythmic fibroblast mobilization during wound healing.* Sci Transl Med, 2017. **9**(415).
15. Wright, K.P., Jr., et al., *Influence of sleep deprivation and circadian misalignment on cortisol, inflammatory markers, and cytokine balance.* Brain Behav Immun, 2015. **47**: p. 24-34.
16. Fadayomi, A.B., et al., *A Systematic Review and Meta-Analysis Examining the Impact of Sleep Disturbance on Postoperative Delirium.* Crit Care Med, 2018. **46**(12): p. e1204-e1212.
17. Roehrs, T.A., et al., *Pain sensitivity and recovery from mild chronic sleep loss.* Sleep, 2012. **35**(12): p. 1667-72.
18. Van Meirhaeghe, J.P., et al., *Improvement in Sleep Patterns After Hip and Knee Arthroplasty: A Prospective Study in 780 Patients.* J Arthroplasty, 2021. **36**(2): p. 442-448.
19. Ding, Z., et al., *Preoperative High Sleep Quality Predicts Further Decrease in Length of Stay after Total Joint Arthroplasty under Enhanced Recovery Short-stay Program: Experience in 604 Patients from a Single Team.* Orthop Surg, 2022. **14**(9): p. 1989-1997.
20. Gong, L., Z. Wang, and D. Fan, *Sleep Quality Effects Recovery After Total Knee Arthroplasty (TKA)--A Randomized, Double-Blind, Controlled Study.* J Arthroplasty, 2015. **30**(11): p. 1897-901.
21. Kirksey, M.A., et al., *Impact of Melatonin on Sleep and Pain After Total Knee Arthroplasty Under Regional Anesthesia With Sedation: A Double-Blind, Randomized, Placebo-Controlled Pilot Study.* J Arthroplasty, 2015. **30**(12): p. 2370-5.
22. Krenk, L., P. Jennum, and H. Kehlet, *Postoperative sleep disturbances after zolpidem treatment in fast-track hip and knee replacement.* J Clin Sleep Med, 2014. **10**(3): p. 321-6.
23. Shen, S.P., et al., *Improved Perioperative Sleep Quality or Quantity Reduces Pain after Total Hip or Knee Arthroplasty: A Systematic Review and Meta-Analysis.* Orthop Surg, 2021. **13**(4): p. 1389-1397.
24. Excellence, N.I.f.H.C.a., *Insomnia: Scenario: Managing long-term insomnia (more than 3 months duration).* 2022.
25. Geenen, R., et al., *EULAR recommendations for the health professional's approach to pain management in inflammatory arthritis and osteoarthritis.* Ann Rheum Dis, 2018. **77**(6): p. 797-807.
26. Koffel, E., et al., *Improving pain and sleep in middle-aged and older adults: the promise of behavioral sleep interventions.* Pain, 2019. **160**(3): p. 529-534.

For peer review only - http://bmjopen.bmj.com/site/about/guidelines.xhtml

27. Whale, K., et al., *The effectiveness of non-pharmacological sleep interventions for people with chronic pain: a systematic review and meta-analysis.* BMC Musculoskeletal Disorders, 2022. **In Press**.

28. Espie, C.A., et al., *The Sleep Condition Indicator: reference values derived from a sample of 200 000 adults.* J Sleep Res, 2018. **27**(3): p. e12643.

29. Craig, P., et al., *Developing and evaluating complex interventions: the new Medical Research Council guidance.* Int J Nurs Stud, 2013. **50**(5): p. 587-92.

30. Whale, K. and R. Gooberman-Hill, *Development of a novel intervention to improve sleep and pain in patients undergoing total knee replacement.* Trials, 2022. **23**(1): p. 625.

31. Kwasnicka, D., et al., *Theoretical explanations for maintenance of behaviour change: a systematic review of behaviour theories.* Health Psychol Rev, 2016. **10**(3): p. 277-96.

32. Orsmond, G.I. and E.S. Cohn, *The Distinctive Features of a Feasibility Study: Objectives and Guiding Questions.* OTJR (Thorofare N J), 2015. **35**(3): p. 169-77.

33. Dawson, J., et al., *Questionnaire on the perceptions of patients about total knee replacement.* J Bone Joint Surg Br, 1998. **80**(1): p. 63-9.

34. Freynhagen, R., et al., *painDETECT: a new screening questionnaire to identify neuropathic components in patients with back pain.* Curr Med Res Opin, 2006. **22**(10): p. 1911-20.

35. Buysse, D.J., et al., *The Pittsburgh Sleep Quality Index: a new instrument for psychiatric practice and research.* Psychiatry Res, 1989. **28**(2): p. 193-213.

36. Zigmond, A.S. and R.P. Snaith, *The hospital anxiety and depression scale.* Acta Psychiatr Scand, 1983. **67**(6): p. 361-70.

37. Herdman, M., et al., *Development and preliminary testing of the new five-level version of EQ-5D (EQ-5D-5L).* Qual Life Res, 2011. **20**(10): p. 1727-36.

38. Al-Janabi, H., T.N. Flynn, and J. Coast, *Development of a self-report measure of capability wellbeing for adults: the ICECAP-A.* Qual Life Res, 2012. **21**(1): p. 167-76.

39. Gale, N.K., et al., *Using the framework method for the analysis of qualitative data in multi-disciplinary health research.* BMC Med Res Methodol, 2013. **13**: p. 117.

40. Tracy, S.J., *Qualitative Quality: Eight "Big-Tent" Criteria for Excellent Qualitative Research.* Qualitative Inquiry, 2010. **16**(10): p. 837-851.

41. Hernandez Alava, M., S. Pudney, and A. Wailoo, *The EQ-5D-5L Value Set for England: Findings of a Quality Assurance Program.* Value Health, 2020. **23**(5): p. 642-648.

42. Pinedo-Villanueva, R., et al., *Identifying individuals with chronic pain after knee replacement: a population-cohort, cluster-analysis of Oxford knee scores in 128,145 patients from the English National Health Service.* BMC Musculoskelet Disord, 2018. **19**(1): p. 354.

For peer review only

Figure 1: CONSORT participant flow diagram

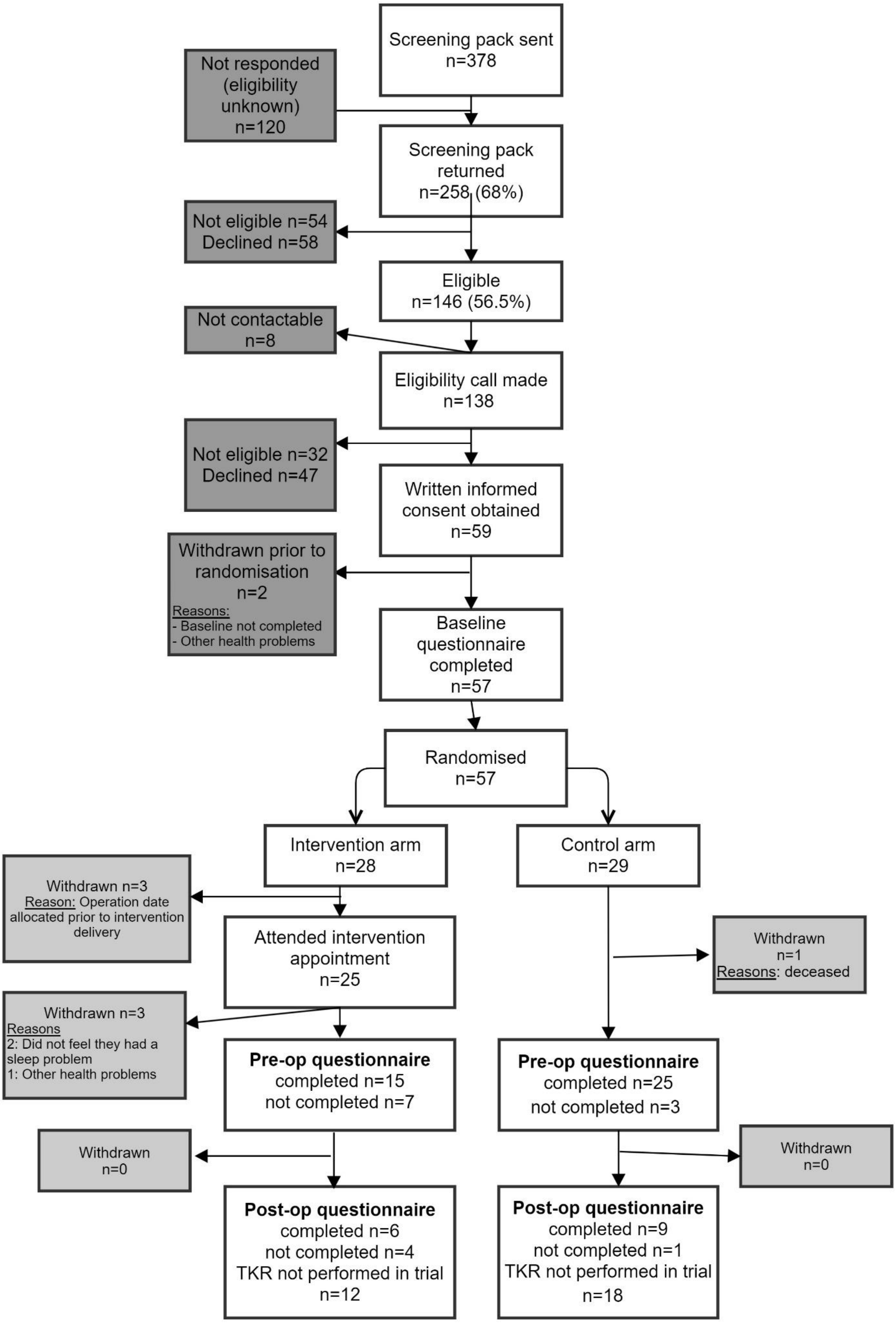

Appendix 1: CONSORT 2010 checklist of information to include when reporting a pilot or feasibility randomised trial in a journal

Item	Description	Reported on line number
Title	Identification of study as randomised pilot or feasibility trial	1
Authors *	Contact details for the corresponding author	9
Trial design	Description of pilot trial design (eg, parallel, cluster)	90
Methods		
Participants	Eligibility criteria for participants and the settings where the pilot trial was conducted	103
Interventions	Interventions intended for each group	122
Objective	Specific objectives of the pilot trial	160
Outcome	Prespecified assessment or measurement to address the pilot trial objectives	169, Table 1
Randomisation	How participants were allocated to interventions	118
Blinding (masking)	Whether or not participants, care givers, and those assessing the outcomes were blinded to group assignment	119
Results		
Numbers randomised	Number of participants screened and randomised to each group for the pilot trial objectives	228
Recruitment	Trial status	N/A
Numbers analysed	Number of participants analysed in each group for the pilot objectives	324, 368
Outcome	Results for the pilot objectives, including any expressions of uncertainty	Objective 1: 228<br>2: 250<br>3: 256<br>4: 271<br>5: 300<br>6: 309<br>7: 315<br>8: 342<br>9: 366
Harms	Important adverse events or side effects	292
Conclusions	General interpretation of the results of pilot trial and their implications for the future definitive trial	448
Trial registration	Registration number for pilot trial and name of trial register	92
Funding	Source of funding for pilot trial	456

Citation: Eldridge SM, Chan CL, Campbell MJ, Bond CM, Hopewell S, Thabane L, et al. CONSORT 2010 statement: extension to randomised pilot and feasibility trials. BMJ. 2016;355.

For peer review only - http://bmjopen.bmj.com/site/about/guidelines.xhtml

Appendix 1: CONSORT 2010 checklist of information to include when reporting a pilot or feasibility trial

Section/Topic	Item No	Checklist item
**Title and abstract**		
	1a	Identification as a pilot or feasibility randomised trial in the title
	1b	Structured summary of pilot trial design, methods, results, and conclusions (for specifi CONSORT abstract extension for pilot trials)
**Introduction**		
Background and objectives	2a	Scientific background and explanation of rationale for future definitive trial, and reasor trial
	2b	Specific objectives or research questions for pilot trial
**Methods**		
Trial design	3a	Description of pilot trial design (such as parallel, factorial) including allocation ratio
	3b	Important changes to methods after pilot trial commencement (such as eligibility criter
Participants	4a	Eligibility criteria for participants
	4b	Settings and locations where the data were collected
	4c	How participants were identified and consented
Interventions	5	The interventions for each group with sufficient details to allow replication, including h actually administered
Outcomes	6a	Completely defined prespecified assessments or measurements to address each pilot 2b, including how and when they were assessed
	6b	Any changes to pilot trial assessments or measurements after the pilot trial commence
	6c	If applicable, prespecified criteria used to judge whether, or how, to proceed with futur
Sample size	7a	Rationale for numbers in the pilot trial
	7b	When applicable, explanation of any interim analyses and stopping guidelines
Randomisation:		
Sequence generation	8a	Method used to generate the random allocation sequence
	8b	Type of randomisation(s); details of any restriction (such as blocking and block size)
Allocation concealment mechanism	9	Mechanism used to implement the random allocation sequence (such as sequentially describing any steps taken to conceal the sequence until interventions were assigned
Implementation	10	Who generated the random allocation sequence, who enrolled participants, and who a interventions
Blinding	11a	If done, who was blinded after assignment to interventions (for example, participants, assessing outcomes) and how
	11b	If relevant, description of the similarity of interventions
Statistical methods	12	Methods used to address each pilot trial objective whether qualitative or quantitative

For peer review only

For peer review only - http://bmjopen.bmj.com/site/about/guidelines.xhtml

**Results**		
Participant flow (a diagram is strongly recommended)	13a	For each group, the numbers of participants who were approached and/or assessed f assigned, received intended treatment, and were assessed for each objective
	13b	For each group, losses and exclusions after randomisation, together with reasons
Recruitment	14a	Dates defining the periods of recruitment and follow-up
	14b	Why the pilot trial ended or was stopped
Baseline data	15	A table showing baseline demographic and clinical characteristics for each group
Numbers analysed	16	For each objective, number of participants (denominator) included in each analysis. If should be by randomised group
Outcomes and estimation	17	For each objective, results including expressions of uncertainty (such as 95% confide estimates. If relevant, these results should be by randomised group
Ancillary analyses	18	Results of any other analyses performed that could be used to inform the future defini
Harms	19	All important harms or unintended effects in each group (for specific guidance see CONS
	19a	If relevant, other important unintended consequences
**Discussion**		
Limitations	20	Pilot trial limitations, addressing sources of potential bias and remaining uncertainty a
Generalisability	21	Generalisability (applicability) of pilot trial methods and findings to future definitive tria
Interpretation	22	Interpretation consistent with pilot trial objectives and findings, balancing potential be considering other relevant evidence
	22a	Implications for progression from pilot to future definitive trial, including any proposed
**Other information**		
Registration	23	Registration number for pilot trial and name of trial registry
Protocol	24	Where the pilot trial protocol can be accessed, if available
Funding	25	Sources of funding and other support (such as supply of drugs), role of funders
	26	Ethical approval or approval by research review committee, confirmed with reference

Citation: Eldridge SM, Chan CL, Campbell MJ, Bond CM, Hopewell S, Thabane L, et al. CONSORT 2010 statement: extension to randomised pilot and feasibility trials. BMJ. 2016;355.

For peer review only - http://bmjopen.bmj.com/site/about/guidelines.xhtml

Appendix 2: The TIDieR (Template for Intervention Description and Replication) Checklist

TIDieR
Template for Intervention Description and Replication

The TIDieR (Template for Intervention Description and Replication) Checklist*:

Information to include when describing an intervention and the location of the information

REST: A pre-operative tailored sleep intervention for patients undergoing total knee replacement: feasibility study for a randomised controlled trial

Item number	Item	Where located ** Primary paper (page or appendix number)	Other † (details)
	**BRIEF NAME**		
**1.**	Provide the name or a phrase that describes the intervention.	__page 4__	______________
	**WHY**		
**2.**	Describe any rationale, theory, or goal of the elements essential to the intervention.	__page 3_____	______________
	**WHAT**		
**3.**	Materials: Describe any physical or informational materials used in the intervention, including those provided to participants or used in intervention delivery or in training of intervention providers. Provide information on where the materials can be accessed (e.g. online appendix, URL).	__page 4-5__	______________
**4.**	Procedures: Describe each of the procedures, activities, and/or processes used in the intervention, including any enabling or support activities.	__page 4-5___	______________
	**WHO PROVIDED**		
**5.**	For each category of intervention provider (e.g. psychologist, nursing assistant), describe their expertise, background and any specific training given.	__ page 4-5__	______________
	**HOW**		
**6.**	Describe the modes of delivery (e.g. face-to-face or by some other mechanism, such as internet or telephone) of the intervention and whether it was provided individually or in a group.	__ page 4-5__	______________
	**WHERE**		

For peer review only

**7.**	Describe the type(s) of location(s) where the intervention occurred, including any necessary infrastructure or relevant features.	___ page 4-5__	______________
	**WHEN and HOW MUCH**		
**8.**	Describe the number of times the intervention was delivered and over what period of time including the number of sessions, their schedule, and their duration, intensity or dose.	__ page 4-5__	______________
	**TAILORING**		
**9.**	If the intervention was planned to be personalised, titrated or adapted, then describe what, why, when, and how.	___ page 4-5__	______________
	**MODIFICATIONS**		
**10.‡**	If the intervention was modified during the course of the study, describe the changes (what, why, when, and how).	___ N/A____	______________
	**HOW WELL**		
**11.**	Planned: If intervention adherence or fidelity was assessed, describe how and by whom, and if any strategies were used to maintain or improve fidelity, describe them.	___page 8-9__	______________
**12.‡**	Actual: If intervention adherence or fidelity was assessed, describe the extent to which the intervention was delivered as planned.	___page 8-9__	______________

** **Authors** - use N/A if an item is not applicable for the intervention being described. **Reviewers** – use '?' if information about the element is not reported/not sufficiently reported.

† If the information is not provided in the primary paper, give details of where this information is available. This may include locations such as a published protocol or other published papers (provide citation details) or a website (provide the URL).

‡ If completing the TIDieR checklist for a protocol, these items are not relevant to the protocol and cannot be described until the study is complete.

* We strongly recommend using this checklist in conjunction with the TIDieR guide (see *BMJ* 2014;348:g1687) which contains an explanation and elaboration for each item.

* The focus of TIDieR is on reporting details of the intervention elements (and where relevant, comparison elements) of a study. Other elements and methodological features of studies are covered by other reporting statements and checklists and have not been duplicated as part of the TIDieR checklist. When a **randomised trial** is being reported, the TIDieR checklist should be used in conjunction with the CONSORT statement (see www.consort-statement.org) as an extension of **Item 5 of the CONSORT 2010 Statement.** When a **clinical trial protocol** is being reported, the TIDieR checklist should be used in conjunction with the SPIRIT statement as an extension of **Item 11 of the SPIRIT 2013 Statement** (see www.spirit-statement.org). For alternate study designs, TIDieR can be used in conjunction with the appropriate checklist for that study design (see www.equator-network.org).

For peer review only

Appendix 3A: Patient interview topic guide

REST patient topic guide

Topic guide to be used flexibly. Interviewer will use probes and follow-up questions where appropriate.

Introduction: Discussion of how the interview will be recorded, issues of confidentiality, anonymisation. **Aim of the study:** To understand patient's perspectives of participating in the study, recruitment, and intervention experiences. **Verbal consent procedure**: Consent for interview, **Any questions?**

Introduction / icebreaker

- Thank you for taking part in the interview. To start us off, could you tell me a little about your **knee pain in the lead up towards your surgery** and the **impact that it has had on your sleep?** PROMPT: What problems have you had? Effect on sleep quality/quantity? Impact on daily activities/wellbeing?
- Have you **taken part in any research studies before?** PROMPT: What? When? What was it like taking part?

Design and conduct of the trial (all participants)

- I'd like to talk to you about **when you first heard** about the REST study. Can you tell me how you found out about it?
- What were your **first thoughts** about taking part?
- Can you tell me ***why* you decided to take part**? PROMPT: Did you discuss your decision with anyone else, for example friends/family member?
- Did you have **any initial concerns** about being involved in the REST study? PROMPT: Could you tell me about them? What helped you to resolve/overcome these concerns?
- Do you still have **any concerns now**, about either the sleep intervention or your participation in the study itself? PROMPT: Expectations versus reality.
- After you expressed your interest in taking part in the REST study, one of the team would have **given** you **a call to discuss the study** with you in more detail. What was having that conversation like for you? PROMPT: Can you recall what information they told you?
- Overall, **what do you think about the information that you have received about the study**: (i) during the initial telephone call, (ii) in the information booklet. PROMPT: Was there enough information / right kind of information? Was it understandable? Was anything missing?
- **How do you feel about being put randomly into a group** to receive either the sleep treatment or usual care? PROMPT: How acceptable do you feel this is?
- **What do you think about the questionnaires** that you have completed, about your knee pain and sleep? PROMPT: Amount of questions, have the questions been relevant, any sections difficult to complete?

Experiences of the sleep intervention (for intervention group only)

- Now I'd like to talk to you about your experiences of the sleep appointment with [NAME] and the advice you were given during it. Overall, **how did you find the sleep appointment**?
- **What did you think about having a telephone or remote / video appointment**? (If relevant) PROMPT: What was it like for you? Pros and cons, did you have any concerns/problems, what device did you use, familiarity with video conferencing, any support needed (e.g., from family member/friend) with setting it up?
- **How did you find the length of the appointment?** PROMPT: Too long/too short. Too rushed or okay. Enough time to ask questions? Did appointment run to time?

- What **advice did the practitioner give** to you about your sleep during the appointment? PROMPT: What was most/least helpful? What do you think about the way in which they gave this information to you? Was it understandable?
- How did you find **talking about/opening up about your sleep** (problems) with the practitioner?
- Which **sleep treatment option did you choose**? PROMPT: CBT-I, relaxation, mindfulness
- **How involved** did you **feel in choosing** this option? PROMPT: How did the practitioner support you in making this decision? Were you given enough information to help you understand the different options?
- Before taking part in the REST study, did you have any **experience or knowledge of CBT, relaxation or mindfulness** techniques? PROMPT: How do you feel this has influenced your experience in the study?
- **What do you think about the sleep treatment so far**? PROMPT: How are you finding doing it, barriers to engagement, facilitating factors (e.g., partner support), what have you enjoyed/not enjoyed, are you finding it helpful, digital and non-digital options (including ease of use and if any support needed to access digital options), (if appropriate) changing experience over time.
- Did you **make any other changes to your sleep** after the appointment? PROMPT: Sleep aids, apps, sleep hygiene.
- How did you find the **4-week follow up phone call** with the practitioner? PROMPT: What was useful/not useful about it. As a result of the phone call, did you make any changes? If appropriate, use participant's intervention uptake questionnaire to guide questioning.
- Overall, **do you feel the appointment and treatment option has had an impact on your sleep**? PROMPT: What impact has it had? What has had the most impact (e.g. chatting to a professional, increasing knowledge/understanding, engaging with the techniques)?
- **Do you think the sleep appointment and treatment option** you chose has had an **impact on your knee pain**? PROMPT: What impact has it had?
- Would you have liked **any further information or additional support** about your sleep or the treatment you chose? PROMPT: When/how would you have liked to receive this?

For <u>usual care group</u> only

- **Since being recruited** into the REST study, has your **sleep changed**? PROMPT: how has it changed, why, when?
- Have you **tried anything to improve your sleep**? PROMPT: what have you tried, how helpful has it been, when did you try this (i.e. prior to or during the REST study)?
- **Since being recruited** into the REST study, has your **knee pain changed**? PROMPT: how has it changed?

Conclusion (<u>all participants</u>)

- Thinking now about your whole experience of taking part in the REST study, **how could we have improved the way in which the study was organised and run**?
- Do you feel that there are **any ways in which your sleep appointment,** or the **follow up phone call, could have been improved**?
- Is there **anything else** you would like to add/talk about that we haven't covered already?
- **Thank participant for their time**. If appropriate, **signpost** participant **to the 'Useful Contacts' sheet.**
- Ask participant if they would like to receive a **brief report** containing the **key findings** from the interview study.

For peer review only

Appendix 3B: ESP intervention training interview topic guide

REST ESP topic guide – intervention training

Topic guide to be used flexibly. Interviewer will use probes and follow-up questions where appropriate.

Introduction: Discussion of how the interview will be recorded, issues of confidentiality, anonymisation.
Aim of the study: To understand experiences of the training session.
Consent procedure: Check written consent, complete consent form if not already done.

Participant information

- **Year of qualification/Years in practice**
- **Role** at the hospital
- **Experience** of participating in **other trials or research** (PROMPT: any experience with sleep interventions, personal experience)

Background information and general sleep education

- I would like to start by asking you about your **experience/overall impression** of the remote training session. **What did you think about the session in general?** (PROMPT: What did you like? What did you not like? What did you enjoy/not enjoy? One long session vs two shorter over two days?)
- Did you **prepare** in any way for the session? (PROMPT: background reading)
- Thinking now about the **information** that you were given **on sleep** and **why sleep is important**:
  - **How understandable** was it?
  - **How useful** did you find it?
- Is there any **additional background information** that you would have **liked to have received** during the training?
- **How confident are you feeling now** about **talking about the background information** with the trial participants? (PROMPT: if not, what additional support / information would you like? How would you like to receive this?)

Appointment delivery

- How do you **plan to structure** your **sessions**? (PROMPT: Recommended timings)

Assessment

- What is your **understanding of the assessment process**?
- Do you **feel clear / confident** about **how to carry out the assessment**? (PROMPT: Using the assessment tool)
- **How do you feel** about **eliciting this information** from the participant?
- What did you think about the **assessment role-play exercise**? (PROMPT: What did you learn from it? How helpful/useful did you find it? Any suggestions about how it could have been done differently? Did you find time to practice the role play exercise after the training?)
- What do you think will be the **main challenges with the assessment process**?

For peer review only

Intervention delivery

- **What did you think** about the **sleep hygiene and education information**? (PROMPT: What did you like/What didn't you like?)
- Is it **clear how to tailor advice** to each participant? (PROMPT: Using the assessment table)
- **What did you think** about the **information** you received **on the specific sleep interventions** you will be recommending?
- **How will you choose** which sleep **intervention** is **most appropriate** for a participant?
- What is your **understanding of the behavioural contract**? (PROMPT: Confident using it, how to choose which areas to highlight, purpose of contract, setting SMART goals)
- What do you think will be **the main challenges with** the **intervention delivery**?

Conclusion

- **How are you feeling about** your **first appointment**? (PROMPT: Do you feel prepared? Is there anything that you will do between now and then to feel more prepared? Is there anything that you are still not sure about/want to know?)
- Is there **any information that you would have liked** in the training that **wasn't provided**?
- Was there **any information** in the training, which you felt was **not useful / needed**?
- Do you have any **suggestions on how to improve the training**?
- Overall, what did you find **most useful** about the training?
- What do you **think about the training manual**? Any **suggestions for improvement**?
- What were **the advantages and disadvantages** of doing the **training over Zoom**?
- Is there **anything else** that you would like to add, or anything you wish to talk about that we haven't covered already?
- **Thank you** for talking to me/your time/**when will be in contact again**.

END

*** Ask ESP about possibility of observing some of their intervention appointments with patients who have not consented to take part in an interview – in order to learn more about the process/patient experience***

For peer review only

Appendix 3C: ESP intervention delivery interview topic guide

REST ESP topic guide – intervention delivery

***Introduction**: Discussion of how the interview will be recorded, issues of confidentiality, anonymisation.*

***Aim of the study**: To understand experiences of delivering the intervention and any additional training needs.*

***Verbal consent procedure**: Reaffirm consent for interview (written consent already given).*

Preparation for delivery

When we first spoke, you told me that before your first intervention appointment, you planned to do [XX]. Did you do this? Did you do anything else to prepare for the appointments? How did undertaking these activities help you to feel more prepared/confident?

Intervention appointment

- **How many sessions** delivered/mode of delivery and **overall experience of delivering** the **sessions: main challenges - for them & patients** (e.g., pragmatic challenges - timing/contacting patients/accessing Zoom/meeting remotely) - & **how overcome**
- **How have you found** undertaking the **assessment process and challenges**? (e.g., Know what questions to ask, eliciting the right information, following the assessment table, drawing out info from patients about sleep issues, patient engagement)
- **How** do you **go about choosing** which **sleep intervention** is best for a participant and any **challenges**? (e.g., selecting most appropriate intervention; shared decision making; patients choosing alternative option)
- **Experience of agreeing the behavioural contract**? (How do you decide on what to include, do participants engage, do you think it is helpful)
- How has the way in which **you deliver the intervention appointment changed over time**? (Refinements made. Increased confidence over time?)
- Impact of mode of delivery on patient engagement/disclosure
- What kind of **questions** have **participants asked** you during their assessment appointments? (Have you felt that you have had the appropriate knowledge/skills to address their questions?)
- **What outcomes would define a successful appointment** – for you and for the participant?

Sleep intervention set up and referral procedure

- **Experience of setting up the interventions** (e.g. clear what to do/what information needed) **& challenges** (e.g. free Headspace trial already used? how was this managed/what did they recommend)
- **Sleepstation referral process** (Any challenges, how long has it taken)
- **Information and support participants want** about **getting started with the sleep interventions**? (Able to give patients the support/info they needed? Paper versions of the documents requested?)

Follow-up phone call

- **How have you found** doing the **4-week follow-up phone calls**? (Challenges getting hold of patients, any

Conclusion

- **Additional training or information needs**? (How/When/Why)
- Thoughts on how **training itself, manual** and **documents could be improved**? (i.e. changes needed)
- **Recommendations for refinements** needed to improve way in which the intervention is delivered? (What do you think has worked well? What hasn't worked so well?)
- How has being part of REST/your REST role **benefited you either personally or professionally**?

***Explore confidence around setting the SMART goals

***Do they feel they understand the theory behind the different interventions and are they able to communicate it to patients?

***Do they feel that they have a good understanding of what SleepStation involves for a patient and are they able to/do they communicate this to patients?

***Have they kept a reflective diary?

For peer review only

Appendix 4: Participant outcomes and health economic data

Comparison of SCI between patients eligible at screening and randomized trial participants

Characteristic	In trial, N = 57[1]	Not in trial, N = 201[1]	p-value[2]
**SCI_Score**	13 (8, 17)	17 (10, 26)	0.004
Unknown	0	49	
[1]Median (IQR)			
[2]Wilcoxon rank sum test			

Pain outcomes at 3 months postoperative (Oxford Knee Score)

Characteristic	Intervention, N = 6[1]	95% CI[2]	Control, N = 9[1]	95% CI[2]
**On-going pain (OKS<14)**				
No on-going pain	5 (83%)	36%, 99%	8 (100%)	60%, 100%
On-going pain	1 (17%)	0.88%, 64%	0 (0%)	0.00%, 40%
Unknown	0		1	
[1]n (%)				
[2]CI = Confidence Interval				

Neuropathic pain outcomes at 3 months postoperative

	**Baseline**		**Pre-operative**		**Post-operative**	
**Characteristic**	Intervention, N = 28[1]	Control, N = 29[1]	Intervention, N = 15[1]	Control, N = 25[1]	Intervention, N = 6[1]	Control, N = 9[1]
**PainDETECT score**	18 (11, 21)	13 (9, 19)	11 (10, 17)	14 (10, 24)	7 (6, 12)	10 (6, 16)
	4	4	6	5	2	3
**PainDETECT category**						
Ambiguous	6 (25%)	5 (20%)	3 (33%)	5 (25%)	0 (0%)	1 (17%)
Neuropathic likely	9 (38%)	8 (32%)	1 (11%)	7 (35%)	1 (25%)	1 (17%)
Nociceptive	9 (38%)	12 (48%)	5 (56%)	8 (40%)	3 (75%)	4 (67%)
Unknown	4	4	6	5	2	3
[1]Median (IQR); n (%)						

EQ-5D-5L and ICECAP scores

Measure	Baseline			Pre-operation			Post-operation		
	Intervention (SD)	Usual care (SD)	Response	Intervention (SD)	Usual care (SD)	Response	Intervention (SD)	Usual care (SD)	Response
EQ-5D-5L	**0.34** (0.30)	**0.47** (0.21)	57/57	**0.35** (0.22)	**0.40** (0.23)	40/57	**0.63** (0.24)	**0.69** (0.13)	14/27*
ICECAP	**0.71** (0.26)	**0.78** (0.14)	56/57	**0.71** (0.23)	**0.80** (0.14)	40/57	**0.83** (0.25)	**0.91** (0.021)	15/27*

*Only collected for those who had TKR within trial.

Intervention costs

**Treatment Cost per person**	**Mean**	**Max**	**Min**
Staff	£103.73	£214.50	£33.00
NHS Treatment cost[§]	30.72	£61.45	£12.29
Societal treatment cost[¥]	£6.59	£19.78	£0
NHS Total (per person)	£134.45	£275.95	£45.29
Societal Total (per person)	£141.04	£295.73	£45.29

[§] Mean - 50% discount given to NHS, Max -no discount, Min - 80% discount offered to NHS.
[¥] Mean -1/3 of participants paid for Headspace and Calm app subscriptions, Max all pay for subscriptions, Min- no one pays for subscription.
*https://www.nice.org.uk/process/pmg36/chapter/economic-evaluation

For peer review only

Appendix 5: Demographics of interviewed participants

Pseudonym	Gender	Age at interview	Intervention or Usual Care	Mode of intervention delivery	Chosen sleep intervention
Florence	Woman	90	Intervention	Telephone	Relaxation
George	Man	64	Usual	N/A	N/A
Joyce	Woman	73	Usual	N/A	N/A
Gail	Woman	64	Usual	N/A	N/A
Charles	Man	79	Intervention	Video	CBT-I
Patricia	Woman	69	Intervention	Video	CBT-I
Gloria	Woman	73	Intervention	Video	Mindfulness
Ruth	Woman	72	Intervention	Video	CBT-I
Arthur	Man	72	Usual	N/A	N/A
Edward	Man	69	Intervention	Telephone	Mindfulness
Jerry	Man	64	Intervention	Video	Relaxation
Steven	Man	58	Usual	N/A	N/A
Rose	Woman	68	Intervention	Video	Mindfulness then CBT-I

For peer review only

Appendix 6: Qualitative themes and quotations

Theme	Subtheme	Quotations
**3. Explore engagement with the REST intervention and adherence to the agreed sleep plan**	Practitioner manner and communication style	*Well I think I was speaking to a professional and when I feel that, that gives me a little confidence in talking to her […] and I found that with [the practitioner] she was really very patient and in discussing her ideas […] she was very encouraging actually …[Florence]*<br>*It's been good. Just talk about things and get it into the open, don't bottle everything up [...] it suits me fine. [Jerry]*<br><br>*Well she was quite firm I felt in telling me about relaxing and how I would relax and she really wanted me to make up my mind then and there and tell her what I was planning to do and that made me feel more positive about it [...] she made me discuss it and having done that I was able to make my own mind up… she was suggesting breathing exercises as well […] there was no insistence at all from her, but on the other hand she was very firm in her suggestions [yes] which made me feel that I must do those things [Florence]*
**1. Evaluate intervention acceptability to patients and health professionals**	Patient acceptability: delivery mode	*I'm using Zoom with family etcetera, so I've used it for over a year now and I'm happy with it […] I'm confident using it [Patricia]*<br><br>*I'm not good on this Wi-Fi stuff, I'm really quite awful on it and my poor son was trying to train me [...] I don't know how to cope or how to use anything with Wi-Fi, I find it very irksome, I really do, very overwhelmingly worrying, I just worry about it. [Florence]*<br><br>*I think this sort of appointment you don't need to be face-to-face. [...] There's no point doing face-to-face. Yes, it would be a 50-mile round trip for me to come to you […] It's fine seeing each other like this to be honest. [...] Travel time, parking, petrol. It's saving a fair bit of money from my point of view. [Patricia]*<br><br>*I don't see a disadvantage. Sitting in my own home rather than going to a hospital where I could pick up more diseases or COVID again. [Gloria]*
	Patient acceptability: appointment length and structure	*There was somebody there who could give me suggestions on the way whilst waiting for my operation, do you know what I mean? It's almost as though she was giving me something to do and think about as I approached the operation … [Florence]*

For peer review only

		The phone call took place not at the right time. But that was fine by me because I wasn't doing anything else. [Patricia]<br><br>*I'm not pleased, I've taken time off work to do this, but I was happy to take time off work to do it but not to be able to do it… [Ruth]*
	Practitioner acceptability: mode of delivery	*I'd be the first to admit that my IT is not as good as it should. I don't do much in the way of IT. […] I'm not the slickest at getting it all up on the computer. […] I find the IT thing a bit of a challenge sometimes [ESP03].*<br><br>*They've all been there ready and waiting and a few times, I've been late finishing the morning clinic because I normally do them on a Monday afternoon. They've all been ready and waiting at their phone or their computer. [ESP03]*
	Practitioner acceptability: intervention handbook and paperwork	*The form was very easy to follow for all the questions [ESP01]*<br><br>*The actual booklet that you're given yes, that's easy. I think maybe I probably need to introduce it a little bit better and what the study is, but then the actual questions are easy to follow, and then it follows onto the SMART goals and things, yes. [ESP01]*<br><br>*I think overall fine, I think sometimes you do feel like you're duplicating quite a lot I think, and when I was actually working through paperwork too quickly and not really sitting down and really going in depth about what the question's asking. Sometimes it feels like, well see above, kind of thing. Maybe that's because I'm classically the kind of person that will write everything down in the first box [RE 4-week call paperwork] [ESP04]*
**6. Evaluate the acceptability of randomisation**		*I understand that that's the only way you can gauge whether what you're doing is of any benefit if it's all randomised. You're not picking out a group of people that are better than another group of people in terms of their symptoms. [Joyce]*<br><br>*I would have particularly liked it if I was one of the people that was offered sort of help with sleep and so on, because the ideal for me would be to find an alternative to running through the highs and lows of my life at three o'clock in the morning would be good. So I was a bit disappointed. [Arthur]*
**9. Collect data on patient-reported outcomes measures to assess data**		*Things affect you differently at different times […] some days, if you're feeling really well, I think you fill it in through rose-tinted glasses. That's the only way I can describe it. If you were having a really good day*

For peer review only

**completion rates and inform the selection of the primary outcome measure and sample size for a full trial**		*and things were going well, you'd fill it in a little bit differently … [Joyce]*

For peer review only

For peer review only
